# A stochastic, physiology-based digital twin model of hemostasis and oxygenation in trauma resuscitation

**DOI:** 10.21203/rs.3.rs-10273801/v1

**Published:** 2026-07-08

**Authors:** Casey Vieni, Anika Iftekharuddin, Kevin Ward, Roland Pittman, Andrew P. Norgan, Matthew D. Neal, Darrell Triulzi, Phillip Spinella, Jason Sperry, Mark Yazer, Jansen Seheult

**Affiliations:** 1. New York University, Grossman School of Medicine, New York, NY; 2. Department of Laboratory Medicine and Pathology, Mayo Clinic, Rochester, MN; 3. Loma Linda School of Medicine, Loma Linda University, California; 4. Weil Institute for Critical Care Research and Innovation, University of Michigan, Michigan; 5. School of Medicine Physiology and Biophysics, Virginia Commonwealth University; 6. Trauma and Transfusion Medicine Center, Department of Surgery, University of Pittsburgh, Pennsylvania; 7. Department of Pathology, University of Pittsburgh, Pennsylvania

## Abstract

Hemorrhagic shock is a common emergency that accounts for more than 10% of global mortality and up to 40% of trauma-related mortality. The Advanced Trauma Life Support (ATLS) guidelines outline resuscitation strategies in patients with massive hemorrhage based on clinical trial and observational data. Early intervention with fluids and/or blood products is recommended during resuscitation as key to maintaining vascular patency, stabilizing a patient’s blood pressure, maintaining tissue oxygenation, and limiting shock. From a transfusion perspective, there is wide heterogeneity in the quality of blood products due to donor and manufacturing variation. The optimal transfusion strategy for massive hemorrhage remains unclear. *In silico* models of resuscitation may offer a means to evaluate the effectiveness and safety of various resuscitation protocols. Here we describe a stochastic multicompartment model of fluid balance and resuscitation that includes (i) cardiovascular hemodynamics, (ii) body fluid compartments and capillary solute exchange, (iii) the ability to alter hemorrhage, resuscitation, and hemostasis parameters, and (iv) tissue oxygenation and capillary-alveolar gas exchange. Building upon deterministic frameworks, this stochastic model more faithfully reflects the clinical heterogeneity of bleeding patients at a level I trauma center. This allows for a proof of concept *in silico* trial comparing crystalloids, conventional component therapy (CCT), i.e. red cell, platelet and plasma components, to cold-stored low titer group O whole blood (LTOWB). In an *in silico* cohort of ATLS class III (>30% and ≤40% blood volume lost) hemorrhage, LTOWB resuscitation reduced the time spent in the critical hemostatic window (platelet count <50×10^9^/L, INR≥2, hemoglobin (Hgb) <8 g/dL and fibrinogen <150 mg/dL) compared with CCT (90.59 *vs*. 147.62 minutes; p=0.04), with no difference in predicted event-free survival (t = 240 minutes; cardiac < 1.5, fluid overload > 10% or SBP > 150% of the starting SBP, or Hgb < 3.0 g/dL). In a separate cohort of ATLS class IV (>40% blood volume lost) hemorrhage, LTOWB yielded a higher predicted event-free survival (74%) versus the CCT arm (69%, p < 0.01). We demonstrate how this *in silico* platform can function as a digital twin for hemorrhagic trauma enabling precision transfusion strategies, and in parallel, an operational twin to support blood banks to forecast blood product demand and inform massive transfusion protocols and clinical trial design.

## Introduction

Hemorrhagic shock remains a leading cause of avoidable death worldwide and is responsible for over 10% of global mortality and up to 40% of trauma-related deaths, with nearly one-third of these occurring within the first four hours post-injury^1–7^. Although military resuscitation guidelines^8,9^ advocate for blood product based resuscitation strategies without crystalloids, Advanced Trauma Life Support (ATLS) guidelines only recently moved towards advocating blood product resuscitation instead of crystalloid infusion as an initial resuscitation step^10–13^. Strong emerging evidence now supports early transfusion of blood products in Class III/IV hemorrhage (>30% blood volume lost) to improve outcomes, in line with recent clinical trial and observational data^14–17^. However, key questions remain around the indications for transfusion, which tend to be based on simple physiological readings especially in the prehospital phase of resuscitation, as well as the choice, order, and rate of blood product infusion, and how additive solutions influence hemostasis and oxygenation during resuscitation^18,19^. While clinical trials remain the gold standard for evaluating resuscitation strategies, ethical constraints, financial burden, and substantial barriers to patient recruitment, including time sensitive enrollment, heterogeneous injury patterns, and limited eligible populations, often render these studies impractical for evaluating precision transfusion strategies^20^.

Volume resuscitation is key to maintaining vascular patency, cardiac output, and oxygen delivery in the setting of hemorrhagic shock^21^. Crystalloid fluids are limited by their lack of oxygen-carrying capacity since they do not have red blood cells (RBCs) or hemoglobin, and lack of hemostatic support in the form of clotting factors; they also lead to metabolic derangements because of their chemical composition and pH^22–25^. In contrast, blood products contain cellular and/or protein constituents that are essential for oxygen delivery and hemostasis: RBCs enhance oxygen delivery and promote platelet margination, platelets are essential for primary hemostasis, and plasma improves hemostasis and endothelial function^26–28^. However, component therapy introduces considerable volumes of non-therapeutic additive solutions used for anticoagulation and RBC preservation^29^. These limitations, along with logistical simplicity, have prompted renewed interest in cold-stored low titer group O whole blood (LTOWB) for civilian trauma resuscitation. The biologic rationale for the preference for LTOWB to resuscitate patients with hemorrhagic shock is based on it being a more potent product when compared to combining a balanced number of red cell, plasma and platelet units, so-called 1:1:1 resuscitation. The hemoglobin, plasma coagulation factors and platelet concentration is higher in LTOWB compared to balanced components since it has approximately a third of additive solution volume relative to balanced conventional component therapy (CCT). LTOWB is also preferred due to the simplicity and speed of providing balanced resuscitation in one unit especially when venous access is limited. Data from retrospective and observational studies suggest improved early hemostasis and potential survival benefit from LTOWB compared to CCT in injured civilian patients to include a meta-analysis of approximately 60,000 patients^30–33^. However, results are mixed, and high-quality randomized controlled trials are ongoing^17,34–38^.

Key unanswered questions in hemorrhagic trauma resuscitation remain: (1) the optimal prehospital crystalloid resuscitation volume, if any, in trauma patients at risk for hemorrhagic shock^39^; (2) which trauma patients derive the greatest benefit from WB versus CCT, particularly in the early post-injury phase^33,40–42^; (3) what is the minimum number of units of LTOWB that will improve outcomes during the initial resuscitation^43–45^; and in particular (4) when a transition from WB to targeted component therapy (goal-directed component transfusion) is warranted for individualized correction of coagulopathy, anemia, or thrombocytopenia^46^. Addressing these challenges requires a mechanistic understanding of dynamic physiological responses to trauma and transfusion, an area where computational modeling may offer critical insights^47^.

Another critical challenge in trauma resuscitation lies in the shortcomings of standard vital signs or hemodynamic measures, such as heart rate (HR), mean arterial pressure (MAP), and central venous pressure (CVP), to reflect tissue-level hypoxia^48^. Decreased organ perfusion in hypovolemic shock leads to impaired oxygen delivery, the buildup of oxygen debt and unmetabolized metabolic products (such as lactic acid), and cellular injury collectively known as shock^49^. If the reduction in oxygen supply and level of incurred oxygen debt is severe or prolonged, cells can become irreversibly damaged and are incapable of resuming normal energy metabolism even with a return of adequate oxygen delivery (${\text{DO}}_{2}$)^50^. As all cells are not irreversibly damaged at once, a critical goal of resuscitation is to anticipate and prevent the transition from viability to irreversibility for the greatest number of cells. However, hemodynamic measures are not adequate predictors of a hypovolemic patient’s ability to be resuscitated^51–54^, as these variables are modified by compensatory mechanisms and may not reflect the extent of cellular injury. Oxygen debt (often used interchangeably with oxygen deficit) occurs when ${\text{DO}}_{2}$ falls below a critical level and cannot meet the demands for oxygen consumption (${\text{VO}}_{2}$)^53,55^. Considerable experimental and clinical evidence suggests that oxygen debt and the change in blood lactic acid levels may reflect the effectiveness of partial volume infusion at achieving resuscitation without predisposing to organ failure^54,56–60^. However, lactate measurements are obtained infrequently during resuscitation, limiting their ability to capture evolving physiological states in real time and are not commonly available in the prehospital setting^61^. Moreover, lactate is a relatively crude and global indicator of oxygen debt that may not reliably reflect changing tissue-level hypoxia in injured patients^62^. As a result, clinical decision-making during hemorrhagic shock often proceeds with limited insight into the adequacy of oxygen delivery and cellular metabolism. Computational modeling offers a powerful and direct approach to understanding tissue hypoxia and accumulation of oxygen debt using a systems-level evaluation of resuscitation strategies under controlled and reproducible conditions.

A growing number of mathematical models have been developed to simulate cardiovascular responses to hemorrhagic shock, ranging from classic Windkessel-type electrical analogs to multi-compartmental models incorporating baroreflex control, fluid shifts, and oxygen transport. A recent review identified seven representative models of hemorrhage and resuscitation physiology, each highlighting tradeoffs between physiological fidelity, model complexity, and clinical applicability^63^. While some models aim for comprehensive physiological representation, such as the Guyton-Coleman framework^64^, others focus on tractable simulations of hemodynamic or reflexive responses using simplified circulatory compartments^65,66^. Notably, most existing models are deterministic in design, simulating a single trajectory without capturing the variability seen across real-world trauma patients^67–72^. Our own previously published deterministic model demonstrated the dilutional effects of conventional component therapy versus LTOWB in a prototypical trauma patient^19^, but could not account for patient-level and blood component-level heterogeneity in determining coagulopathy, body fluid shifts or transfusion response, limiting this model’s generalization.

To address these limitations, we have developed a unified, stochastic multicompartment computational model of the resuscitation and outcomes for a patient with severe bleeding from trauma that integrates cardiovascular hemodynamics, body fluid compartments and capillary solute exchange, hemostasis, tissue oxygenation, and capillary-alveolar gas exchange. Building upon prior deterministic frameworks, our model incorporates inter-patient variability in blood volume, cardiac output, trauma severity, and transfused product composition to more faithfully reflect clinical heterogeneity. As a proof of concept, we simulate an *in silico* clinical trial comparing LTOWB to CCT in ATLS Class III (>30% and ≤40% blood volume loss; see methods for further details) and IV (>40% blood volume loss) patients. This platform functions as a **digital twin** for hemorrhagic trauma, enabling precision transfusion strategies tailored to patient physiology and injury severity, and establishes a foundation for future incorporation of AI into physiology-constrained digital twin systems in trauma and major bleeding. In parallel, A goal directed therapy module was implemented whereby labs are assessed “in real time” with transfusion decisions based on a patient’s vital signs and laboratory data to balance organ perfusion and tissue oxygenation, while minimizing rebleeding risk. This framework can serve as an **operational twin** to support blood banks and transfusion services by helping forecast blood product demand, inform the optimum contents of massive transfusion protocol (MTP) packets, and optimize inventory management under variable trauma loads. Ultimately, this model enables mechanistic exploration of resuscitation strategies under physiologic stress and supports the development of precision trial designs that target the right resuscitation approach to the right patient, at the right time.

## Results

### Cardiovascular hemodynamic, capillary solute exchange and hemostatic modules

**Figure 1:**
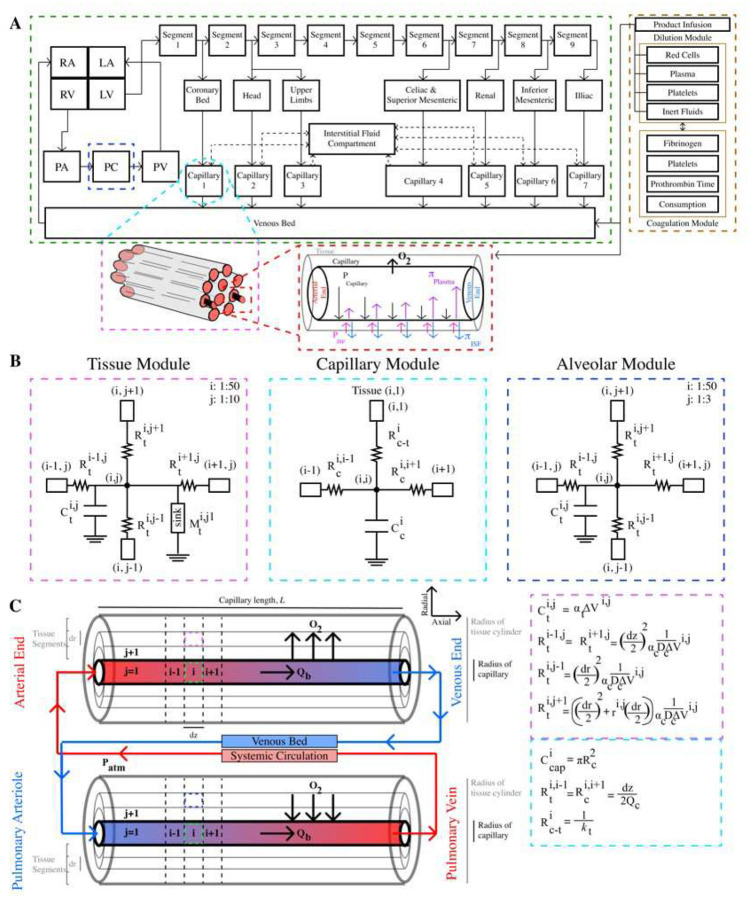
Schematic representation of the multicompartment model of hemostasis and oxygenation. (A) Overall schematic of our multicompartment model consisting of a cardiovascular and body compartment module (green box), a resuscitation and infusion module (orange), a solute/solvent capillary transfer module (red box), and a tissue oxygenation (magenta) and pulmonary capillary module (blue box). (B) The oxygenation modules were modeled as RC electrical circuits at the tissue, capillary, and alveolus. (C) A schematic representation of the connection between the capillary/tissue and capillary/alveolar segments of the oxygenation module with the key equations for calculating the tissue or capillary resistance and capacitance.

**Figure 2:**
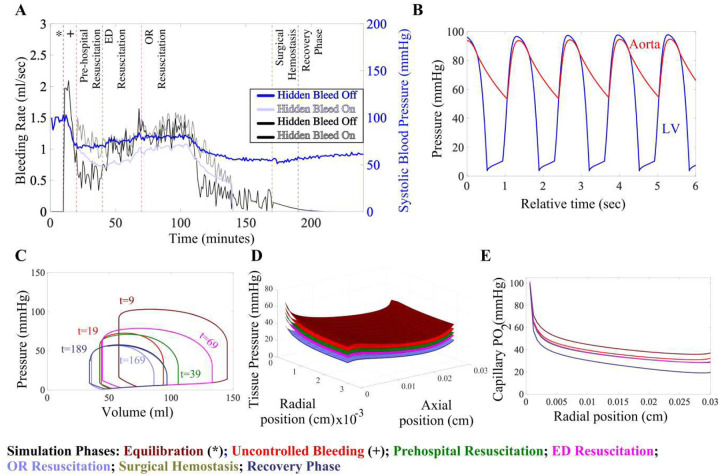
Illustrative example of a simulation of a single deterministic patient in the comprehensive model. (A) Changes in bleeding rate and systolic blood pressure (SBP) over time in this study’s multicompartment dynamic model of a massively bleeding adult trauma patient. This simulation consisted of 6 phases: an initial equilibration phase (minute 1–10; maroon, *), an initial injury phase (minute 11–20; red, +); a prehospital resuscitation phase (minute 21–40; green), an emergency department (ED) resuscitation phase (minute 41–70; pink), an operating (OR) resuscitation phase (minute 71–170; light blue) during which surgical hemostasis is achieved (minute 170, dark green), and a recovery phase (minute 191–250; indigo) with a vertical dashed line indicating the transition between phases. 1L of crystalloid fluid was administered in the pre-hospital resuscitation phase and whole blood (WB) was administered during the hospital resuscitation phases. A light blue and gray line reflect the SBP and bleeding rate respectively, for this patient with the “hidden bleed” parameter turned on. (B) The cardiovascular module simulates hemodynamics in the left ventricle (blue) and the aorta (red). (C) Volume contraction is observed in pressure volume curves of the left ventricle at the end of equilibration (maroon) versus ten minutes after bleeding begins (red) with partial volume recovery at the start of the ED phase (pink). (D) Decreased tissue oxygenation and (E) capillary ${\text{PO}}_{2}$ is observed following trauma compared to equilibration.

A detailed overview of the integrated model is presented in the Online Methods section. Briefly, previously published deterministic models of trauma resuscitation with cardiovascular, fluid exchange, and oxygenation modules were adapted and combined into a multicompartment dynamic model of a massively bleeding adult trauma patient. This model incorporates a cardiovascular hemodynamic module, a capillary solute exchange module, a hemostatic module, tissue and alveolar capillary oxygenation modules, and a resuscitation module (Figure 1). Deterministic simulations reproduced previously reported outputs for cardiovascular hemodynamics (Figure 2B–C) and tissue oxygenation (Figure 2D–E), as well as hematologic indices and dilutional coagulopathy following traumatic injury and resuscitation with WB or CCT (Figure 2A). The simulation was structured into six sequential phases: equilibration, injury, prehospital resuscitation (1 L crystalloid fluid [0.9% NaCl or Lactated Ringers/lactate buffered solution^73^]), emergency department (ED) transfusion, surgical intervention (OR), and recovery. As systolic blood pressure (SBP) rose during resuscitation, bleeding increased until hemostasis was achieved at 160 minutes (Figure 2A).

Integration of prior model components enabled detailed simulation of cardiovascular parameters across multiple time points during resuscitation. For instance, left ventricular (LV) and aortic pressures (Figure 2B) were continuously modeled throughout the simulation, while chamber-specific pressure-volume (PV) loops captured progressive reductions (e.g. 10 minutes after the onset of bleeding) and expansions (e.g. at the conclusion of the ED phase) in filling volumes during key phases (Figure 2C). In parallel, the tissue oxygenation module revealed the spatial distribution in oxygen pressure across the capillary following injury, with marked decreases observed between the equilibration phase and post-injury (Figure 2D). During equilibration, steady state oxygen partial pressure decreased from approximately 101 mmHg to approximately 60 mmHg within the first ~6 capillary segments along the capillary length, then declined slowly thereafter to a nadir of 35.9 mmHg. Oxygen extraction across the capillary axial length was higher post-injury in the uncontrolled bleeding and ED resuscitation phases, reaching a nadir of 25.3 mmHg (Figure 2E).

To support these dynamic outputs, several enhancements to the cardiovascular model were implemented and are described in detail in the Methods section (Figure S1). Briefly, a time-varying heart rate (HR; Figure S1C) was incorporated, which necessitated corresponding adjustments to time-dependent chamber elastance functions (Figure S1A). These changes modulated chamber-specific pressure and volume dynamics across the cardiac cycle and led to expected decreases in diastolic filling at higher heart rates (Figure S1D). Additional modifications to the equations governing chamber pressure generation ensured physiologic consistency between systolic and diastolic phases (see Methods), successfully replicating expected waveform morphology (Figure S1B).

A “hidden bleed” parameter was incorporated to simulate difficult-to-control hemorrhage, such as retroperitoneal or thoracic bleeds (see Methods, Figure S2, and Table S2). A “consumptive coagulopathy” parameter was also incorporated to reflect the subset of patients who develop early consumptive coagulopathy and hyperfibrinolysis (see Methods, Figure S3, and Table S2).

### Resuscitation module

Multiple resuscitation strategies were implemented to compare the effectiveness of different transfusion approaches (Figure S4). To replicate the results in our previous work^19^, resuscitations with 18 units of LTOWB versus 18 units of CCT were simulated, the latter structured as three massive transfusion protocol (MTP) packets with a 6:6:1 ratio of RBCs, plasma, and platelets (Figure S4 and S5). Outcome measures included hemoglobin (Hgb), plasma volume (PV), interstitial fluid (ISF) volume, international normalized ratio (INR), platelet count and fibrinogen concentration (Figure S5). LTOWB resuscitation resulted in higher Hgb and fibrinogen levels, more stable platelet count and lower INR during the ER and OR resuscitation phases compared to CCT (Figure S5B, E–G). In contrast, simulations using CCT demonstrated greater plasma and ISF volumes (Figure S5C–D). LTOWB reduced the time spent in critical sub-hemostatic intervals, defined as INR ≥1.5, fibrinogen <150 mg/dL^74^, and/ or platelet count <50 × 10^9^/L, and yielded a higher hemoglobin nadir compared to CCT (Table S1).

### Tissue and alveolar capillary oxygenation module

Tissue hypoperfusion and hypoxemia were modeled using an expanded capillary–tissue oxygenation module, which operated throughout the simulation to track changes induced by trauma and resuscitation. To overcome limitations in prior implementations that assumed a fixed oxygen concentration at the capillary inlet, an alveolar-capillary module was introduced to simulate oxygen diffusion into the pulmonary capillary (Figure 1). In this module, alveolar ${\text{PO}}_{2}$ was held constant at 104 mmHg, enabling dynamic adjustment of alveolar capillary oxygenation based on hemoglobin concentration and blood flow rate (Figure S6).

**Figure 3:**
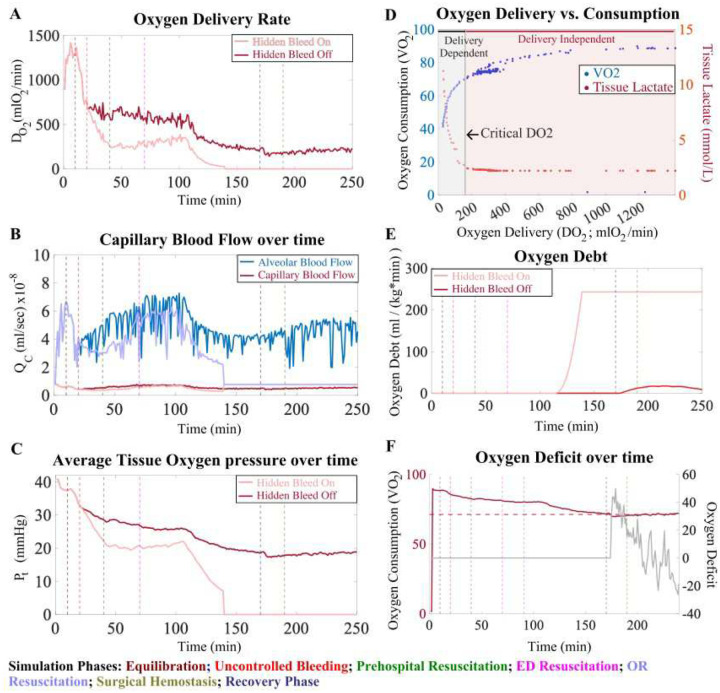
Illustrative example of tissue oxygenation in a single deterministic patient in the comprehensive model. (A) The oxygen delivery rate over time, (B) the capillary blood flow (${\text{Q}}_{\text{C}}$) over time, and (C) the average tissue ${\text{O}}_{2}$ pressure (${\text{P}}_{\text{t}}$) over time in this study’s multicompartment dynamic model of a massively bleeding adult trauma patient. (D) As oxygen delivery (${\text{DO}}_{2}$) acutely decreases, oxygen extraction/consumption (${\text{VO}}_{2}$; blue) decreases beyond a critical ${\text{DO}}_{2}$ threshold (gray), and ${\text{VO}}_{2}$ becomes delivery dependent and lactate levels (red) begin to increase. (E) As oxygen consumption becomes delivery dependent, an oxygen debt occurs leading to an increase in unmetabolized metabolic acids. (F) As oxygen decreases below the critical ${\text{DO}}_{2}$, oxygen deficit acutely increases. Then, as ${\text{DO}}_{2}$ increases above the critical ${\text{DO}}_{2}$, oxygen debt stops accumulating as oxygen deficit returns to ≤ 0. In figures measuring variables over time, the vertical lines correspond to the end of the equilibration phase (maroon), the end of physiologic uncontrolled bleeding at which point emergency medical services (EMS) arrive (red), the end of prehospital resuscitation (green), the end of emergency department (ED) resuscitation and the start of operating room (OR) resuscitation (pink), surgical hemostasis (dark blue), and the start of the recovery phase (dark green). Where indicated the pale pink and the dark red curves correspond to a single determinist patient with the “hidden bleed” parameter turned on or off, respectively.

In deterministic simulations, oxygen delivery (${\text{DO}}_{2}$) was highest during the equilibration phase ($\sim 1400\;\text{mL}\;{\text{O}}_{2}/\text{min}$) and decreased to $\sim 600\;\text{mL}\;{\text{O}}_{2}/\text{min}$ approximately 10 minutes post-injury, then either remained stable in the absence of a “hidden bleed” or decreased to $\sim 250\;\text{mL}\;{\text{O}}_{2}/\text{min}$ when hidden bleeding was present (Figure 3A). Tissue capillary blood flow remained relatively stable across the simulation (Figure 3B). The decrease in average tissue oxygen pressure was more pronounced in the setting of a hidden bleed, falling to ~22 mmHg by the end of the pre-hospital resuscitation phase (Figure 3C).

An additional module was implemented to track oxygen consumption (${\text{VO}}_{2}$) and to compare it against ${\text{DO}}_{2}$ in real time. When ${\text{DO}}_{2}$ fell below a critical threshold, oxygen consumption became supply-dependent, and oxygen debt accumulated (for example: Figure 3D–E). Acute reductions in ${\text{DO}}_{2}$ below the critical threshold led to increases in tissue lactate levels (Figure 3D) allowing real time monitoring of lactate throughout the simulation. Oxygen debt accumulation started at 160 minutes in the absence of a hidden bleed (Figure 3E) and when hematocrit recovered after surgical hemostasis was achieved, oxygen deficit declined with resolution of the accumulated oxygen debt (Figure 3E–F).

### Stochastic implementation of resuscitation model

A diverse cohort of trauma patients representative of those encountered at a level I trauma center was simulated. Distributions of randomized parameters are shown in Figure S7, with full parameter derivations provided in Tables S3–4 and the Methods section. Patient characteristics including weight, height, and age were randomized based on U.S. census data, with sex randomized based on distributions of patients who received RBC or LTOWB within four hours of arrival to the trauma center from the Trauma Quality Improvement Program (TQIP) database^75^. These variables were used to derive individualized estimates of initial systolic blood pressure (SBP) and blood volume. ATLS class assignments were drawn from reported population distributions in trauma registries, with Class III (>30% and ≤40% blood volume loss) and IV (>40% blood volume loss) patients representing the majority of cases^76^.

The “hidden bleed” parameter was scaled to approximate observed mortality rates for each ATLS class (Figure S8A–C)^77–79^. To simplify initial implementation, all patients were assumed to have sustained similar injury mechanisms, i.e., variations in mechanism such as blunt versus penetrating trauma were not modeled. Patients were assumed otherwise healthy at baseline, and non-hemorrhagic forms of shock (e.g., distributive shock from spinal injury) were excluded. Approximately 25% of ATLS Class IV (>40% blood volume loss) and 5% of Class III (>30% and ≤40% blood volume loss) patients were randomly assigned higher consumption rates of plasma, fibrinogen, and platelets (“consumptive coagulopathy” parameter), consistent with reported TIC incidence and associated laboratory criteria (e.g., INR ≥1.3–1.5, Figure S8D–F)^80–84^.

Patient mortality was defined by a calculated cardiac index (CI) falling below 1.5 L/min/m^2^^85,86^ or a hemoglobin value below a critical threshold of 3.0 g/dL^87^. Patients were classified as fluid overloaded if their percent fluid overload score exceeded 10% or if their SBP rose to 150% of the baseline value^88^ (Figure S9).

### In silico resuscitation strategy comparison

**Figure 4:**
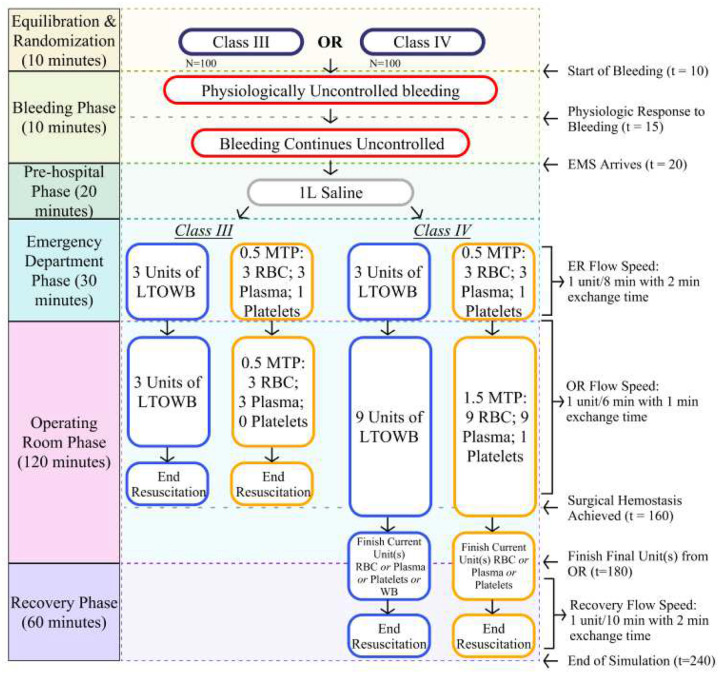
Schematic representation of the *in silico* trial comparing resuscitation with low titer group O whole blood (LTOWB) vs. conventional component therapy (CCT) using the stochastic multicompartment model. This simulation consisted of 6 phases: an initial equilibration phase (minute: 1–10) during which patients were assigned to either ATLS class III (>30% and ≤40% blood volume lost) or class IV (>40% blood volume lost). Then an initial injury phase (minute: 11–20) during which bleeding first occurs at a defined rate (physiologically uncontrolled, minute: 11–15) before becoming unrestrained (physiologically controlled, minute: 15–20). Then EMS arrives and gives the patient 1 L of saline (minute: 20–40). Upon arrival at the emergency department (ED) (minute: 41–70) a patient is then assigned to the LTOWB or CCT arms and receives 3 units of blood products at a rate of 1 unit per 8 minutes, with a 2 minute exchange time between units. Blood products are continued in the operating room (OR) phase (minute: 71–190) with a faster infusion rate of 1unit/6min with 1 minute exchange time. ATLS class III patients continue to receive 3 more units (or 0.5 MTP packets) of blood products for a total of 6 units, while ATLS class IV patients receive 9 more units (or 1.5 MTP packets) for a total of 12 units. Surgical hemostasis is then achieved in the OR phase (minute: 170), and the patient transitions to the recovery phase (minute 191–240; indigo) where any remaining blood products are completed at a rate of 1 unit per 12 minutes.

**Figure 5:**
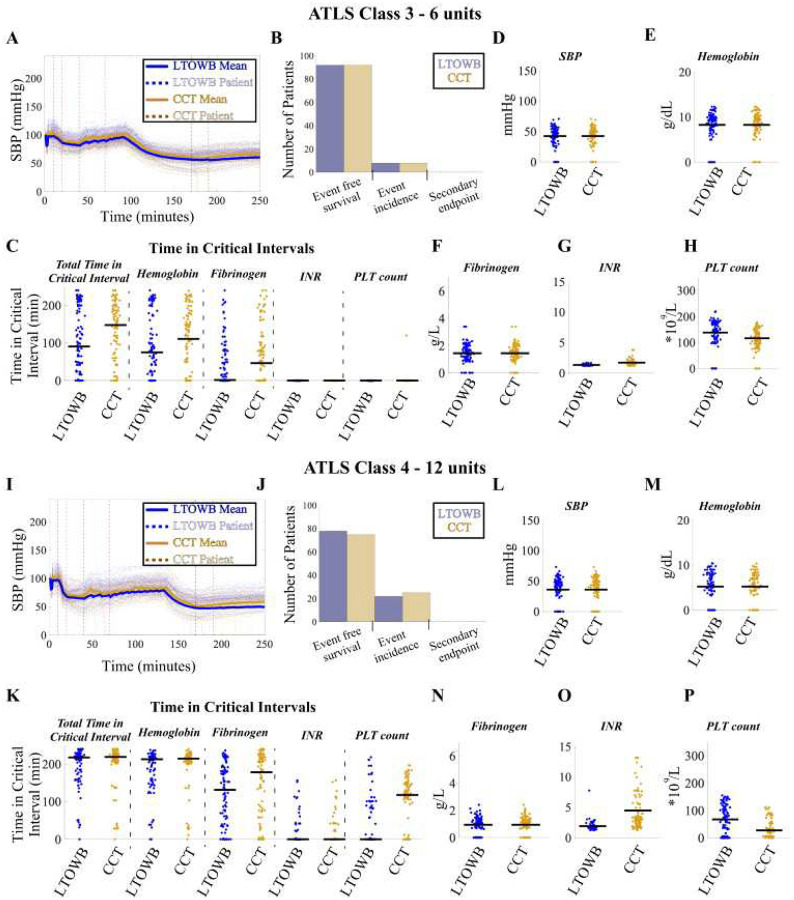
Illustrative example of a simulation of stochastic patients comparing low titer group O whole blood (LTOWB; blue) resuscitation to conventional component therapy (CCT; orange) resuscitation. (A) Systolic blood pressure (SBP) over time for 100 ATLS class III (>30% and ≤40% blood volume lost) patients who received 6 units of LTOWB or CCT. (B) At the end of the simulation ~90% of the patients survived in both the LTOWB and CCT arms. (C) Patients in the LTOWB arm vs. CCT arm spent less time in critical intervals for INR ≥1.5, fibrinogen level <150 mg/dL, platelet (PLT) count <50 × 10^9^/L, and Hemoglobin < 8g/dL over the course of the simulation (p<0.001 based on median regression for each panel). The end of simulation values for (D) SBP, (E) Hemoglobin, (F) Fibrinogen, (G) INR, and (H) PLT between treatment arms. (I). SBP over time for 100 ATLS class IV (>40% blood volume lost) patients who received 12 units of LTOWB or CCT. (J) At the end of the simulation, 74% of patients in the LTOWB arm reached event-free survival the simulation vs. 68% of patients in the CCT arm. (K) Patients in the LTOWB arm vs. CCT arm spent less time in critical intervals for INR ≥1.5, fibrinogen level <150 mg/dL, PLT count <50 × 10^9^/L, and Hemoglobin < 8g/dL. End of simulation values for (L) SBP, (M) Hemoglobin, (N) Fibrinogen, (O) INR, and (P) PLT count between treatment arms.

A proof-of-concept *in silico* resuscitation trial was conducted using 100 simulated ATLS Class III (>30% and ≤40% blood volume lost) and 100 simulated ATLS Class IV (>40% blood volume lost) patients randomized to receive either LTOWB or CCT over 240 minutes (Figure 4, Appendix S4) with equal infusion rates in both arms (see Methods). While rapid infusers can achieve higher flow rates and infuse a unit within ≈1–2 minutes^89,90^, infusion rates were set at specific rates depending on the phase (ex. 1 unit in 6 minutes in the OR phase) and the overall resuscitation strategy was designed to be modifiable depending on the user’s need. Class III patients received a total of 6 units of LTOWB or one CCT MTP (6 RBCs, 6 plasma units, and 1 dose of platelets), while Class IV patients received 12 units of WB or two CCT MTPs. Survival, laboratory parameters, and cumulative exposure to critical sub-hemostatic thresholds were logged throughout the simulation (Figure 5; Table S1).

In the ATLS Class III cohort, event-free survival to the end of the simulation was 88% for LTOWB-treated patients and 87% for CCT-treated patients (p = 1.0, Figure 5B). In the Class IV cohort, event-free survival to the end of the simulation was lower in the CCT arm (74% for LTOWB-treated patients and 68% for CCT-treated patients, p < 0.01, Figure 5J). Time to event was not significantly different between LTOWB or CCT-treated patients in either Class III or Class IV patients (p = 0.68 & p = 0.30 respectively). The total incidence of adverse events, including fluid overload, low cardiac index, or critical anemia, was modestly lower in the LTOWB arm across both cohorts (Table S1).

Time spent in critical sub-hemostatic intervals was analyzed as a function of treatment arm. For Class IV patients, LTOWB resuscitation reduced the cumulative time spent below Hgb <8 g/dL, fibrinogen <150 mg/dL platelet count <50×10^9^/L, and INR ≥1.5 compared to CCT (Figure 5K). Median total time spent in any critical interval was lower in the LTOWB group (p < 0.001, median regression; Table S1). This pattern was also observed in Class III patients, although the magnitude of the difference was smaller (Table S1).

End-of-simulation laboratory values did not differ significantly between groups. SBP, hemoglobin, fibrinogen, INR, and platelet counts were comparable at the final timepoint for both Class III (Figure 5D–H) and Class IV (Figure 5L–P) patients. This indicates that both resuscitation strategies ultimately achieved similar hemodynamic and hematologic endpoints by the end of the simulated protocol. However, temporal dynamics of hemostatic recovery, as reflected by critical interval duration, differed between the arms.

### Goal Directed Therapy

**Figure 6:**
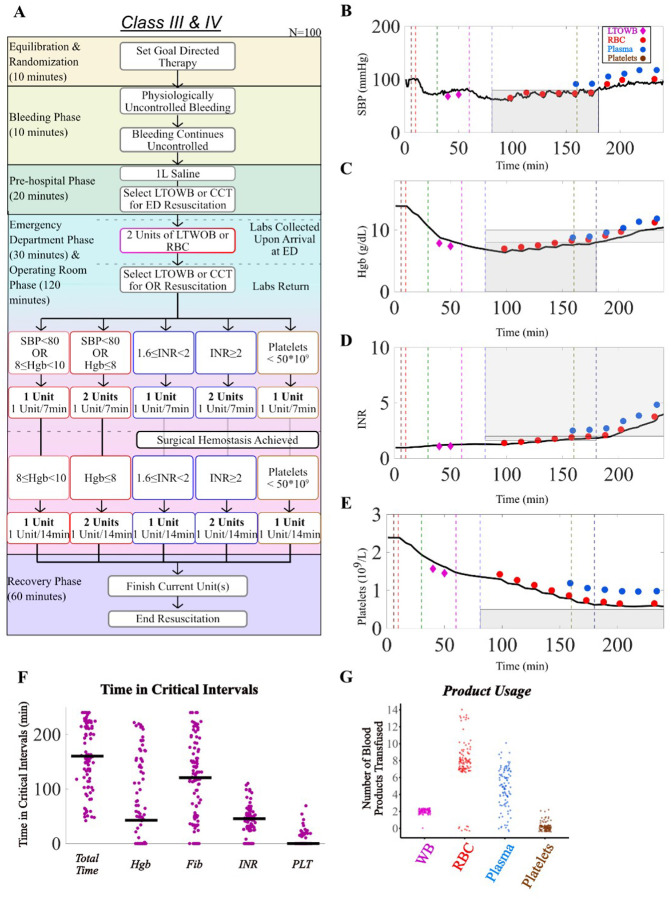
Illustrative example of the modular Goal Directed Therapy (GDT) module. (A) Schematic representation of the GDT resuscitation where a patient receives two units low titer group O whole blood (WB) upon presentation to the emergency department (ED). Then, the collection of laboratory samples is simulated and result 15 minutes after collection. Resuscitation then proceeds with one unit of red blood cells (RBC) depending on the average systolic blood pressure (SBP) or hemoglobin (Hgb) value, one unit of plasma depending on the INR, and a unit of platelets depending on the average platelet value. Surgical hemostasis is achieved in the OR phase (minute: 170) with additional units of RBC given in the recovery phase as the SBP recovers. (B-E) Example (B) SBP, (C) Hemoglobin, (D) INR, and (E) Platelets of a patient in the GDT module. (F) Time in critical intervals for 100 stochastic patients for Hemoglobin < 8g/dL, INR ≥1.5, fibrinogen level <150 mg/dL, and PLT count <50 × 10^9^/L, (G) Total number of blood products utilized for stochastic patients.

Goal directed therapy (GDT) uses continuous hemodynamic and hemostatic assessment (vital signs and laboratory data) of a patient to balance organ perfusion and tissue oxygenation, while minimizing rebleeding risk^10,91^. A GDT module was implemented to approximate real-time clinical decision-making and maintain fidelity with real-world trauma resuscitation practices (Figure 6A). Upon arrival to the ED, two units of LTOWB are administered, and simulated specimens are drawn immediately for laboratory testing. Laboratory results (hemoglobin, fibrinogen, INR, platelet count) return after a simulated 15 minute-turnaround, at which point resuscitation is guided by predefined laboratory thresholds^92^. RBC, plasma, or platelets are administered as needed according to predefined parameters depending on if bleeding is brisk or uncontrollable or controlled after surgical hemostasis is achieved (Figure 6B–E). *In silico*, this resuscitation strategy resulted in comparable time spent within the critical laboratory intervals and event-free survival relative to CCT-guided resuscitation (Table S1, S2; Figure 6F) while reducing overall blood product utilization (Figure 6G).

### Digital Twin Emulation of the Prehospital Air Medical Plasma (PAMPer) Trial

**Figure 7:**
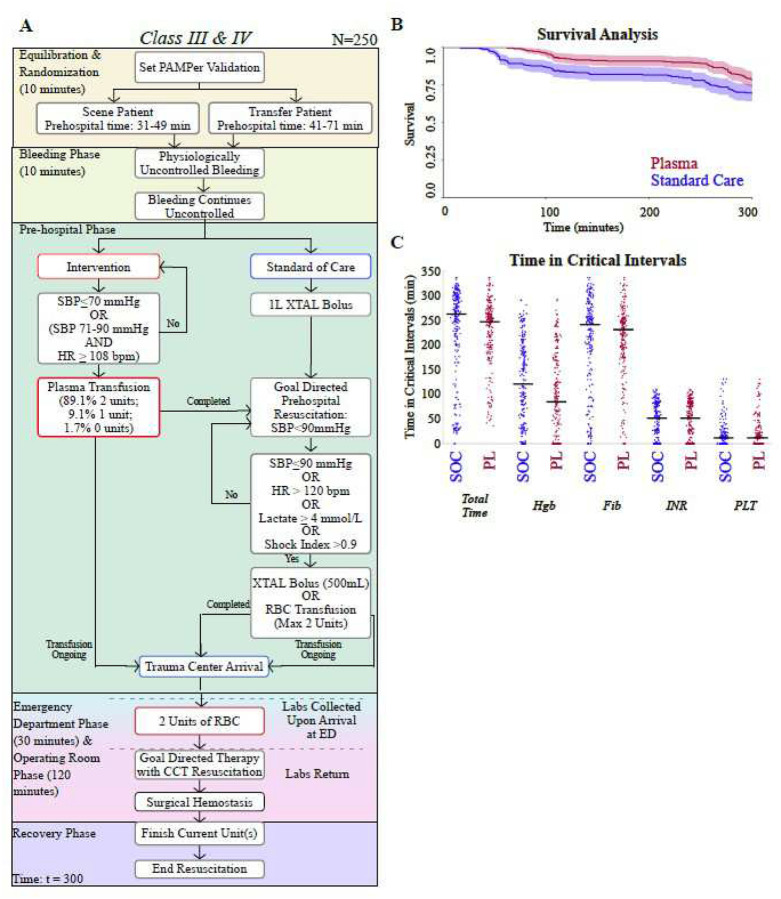
Results for emulation of the Prehospital Air Medical Plasma (PAMPer) trial with 250 simulated patients. (A) Schematic representation of the PAMPer module inclusion/exclusion criteria, along with goal directed therapy (GDT) resuscitation in the pre-hospital and hospital setting. GDT in the pre-hospital setting consisted of simulated clinical decision before transfusing a red blood cell (RBC) unit or infusing a crystalloid (XTAL) bolus. Following transfusion of 2 units of RBC units upon presenting to the emergency department (ED), GDT in the hospital setting consisted of simulated laboratory evaluation, followed by resuscitation with conventional components (CCT) based on a patient’s systolic blood pressure (SBP), hemoglobin (Hgb), INR, or platelet count. Surgical hemostasis is achieved in the OR phase (100 minutes after transitioning to the OR) with additional units of RBC given in the recovery phase as the SBP recovers. Simulation results were truncated at minute 300 regardless of amount of time spent in the recovery phase. (B) Kaplan-Meier survival curves for simulated patients in the standard of care (blue; SOC) arm or the plasma intervention arm (red; PL) over the first 300 minutes of the simulation. (C) Time in critical intervals for 250 stochastic patients in the SOC or PL arm for Hemoglobin < 8g/dL, INR ≥1.5, fibrinogen level <150 mg/dL, and PLT count <50 × 10^9^/L.

To demonstrate the applicability of the model as a physiology-based digital twin in a clinical trial setting, we recreated key design elements of the Prehospital Air Medical Plasma (PAMPer) trial (Figure 7), which evaluated prehospital plasma resuscitation compared with standard care (not including plasma administration) in severely injured patients. The PAMPer trial reported early separation of survival curves between treatment arms within the first three hours of injury in favor of prehospital plasma resuscitation.^17^ A dedicated module was implemented to approximate trial inclusion and exclusion criteria and simulate prehospital decision making (Figure 7A). In this framework, 250 simulated patients received either 1L crystalloid fluid or were evaluated for 2 units of plasma administration, followed by GDT with additional crystalloid or RBC transfusion (Figure S10). *In silico,* early separation between treatment arms was observed, qualitatively consistent with the temporal patterns reported in the PAMPer trial (Cox proportional hazards model: HR: 0.64, 95% CI: 0.45–0.90; log-rank chi-square test: χ^2^ = 6.5, p = 0.01; Figure 7B). The intervention (prehospital plasma) arm demonstrated reduced cumulative time within critical laboratory intervals (p ≤ 0.01, median regression; Figure 7C).

## Discussion

This study presents a unified, stochastic, multicompartment in silico model of trauma resuscitation that integrates cardiovascular dynamics, fluid balance, hemostasis, and tissue oxygenation into a single simulation platform. By extending prior deterministic frameworks and incorporating patient-level stochastic variability, this model serves as both a digital twin, capable of simulating precision transfusion therapy at the patient level, and an operational twin that can inform systems-level decisions such as blood product inventory management and massive transfusion protocol design.

Earlier models of hemorrhagic shock that focused narrowly on baroreflex regulation^93–96^, pressure-volume relationships^97^, solute flux^68,98^, or baroreflex-cardiac coupling and ${\text{DO}}_{2}/{\text{VO}}_{2}$ balance^66,72^ lacked fully integrated representations of capillary-alveolar exchange, pulsatile cardiac function, dynamic tissue oxygenation, hemorrhage and resuscitation, and coagulopathy progression. In this model, these elements are unified and further extended to reflect clinical heterogeneity through stochastic assignment of patient characteristics, blood product composition, injury severity, and coagulopathy burden, in order to provide a high-resolution, high-fidelity simulation of trauma physiology across multiple organ systems. Then, a goal-directed, protocol-driven approach can be taken with specific hemodynamic targets to optimize tissue perfusion during the critical early phase of resuscitation to prevent the progression of shock and subsequent organ dysfunction^99^.

A key strength of this model lies in its modular integration of physiology: pressure-volume relationships within cardiac chambers evolve based on time-varying elastance and heart rate; solute and fluid movement across compartments is governed by oncotic and hydrostatic gradients; and oxygen delivery is dynamically recalculated as a function of hemoglobin, hemoglobin oxygen saturation, and perfusion. This allowed the modeling of key physiologic transitions, such as the development of oxygen debt, coagulopathy (endogenous, consumptive and dilutional, see Methods), and resuscitative reversal or death, under a range of resuscitation scenarios. The inclusion of an alveolar module permits physiologic oxygenation at the pulmonary interface, while the tissue module enables mapping of oxygen tension across vascular space and time. These capabilities are critical to understanding the nuanced impact of blood product composition and timing on metabolic recovery.

The stochastic implementation adds a key dimension to the simulation environment. By introducing inter-patient variability in age, sex, body size, bleeding rate, trauma class, baseline hematologic values, and blood product characteristics, the model approximates the spectrum of trauma patients seen in a level I trauma center. Similarly, mechanistic models of human physiology can support clinically grounded AI by encoding biological constraints, latent state dynamics, and causal relationships that are difficult to learn from data alone. This allows for *in silico* clinical trials that test hypotheses under tightly controlled conditions across heterogeneous simulated populations and provides a mechanistic framework for future physiology-constrained AI digital twin systems in trauma and major bleeding. In engineering domains, such as autonomous driving^100^ and aerospace^101^, physics-informed digital twins constrain learning algorithms within physically plausible limits. This enables hybrid models that combine mechanistic simulation with machine learning to support patient-specific inference, counterfactual simulation, and clinical decision support, while maintaining interpretability and biological plausibility.

While we designed an *in silico* trial comparing LTOWB *versus* CCT in the hospital setting, the simulation could easily be modified to compare LTOWB *versus* CCT in pre-hospital settings^19^, different simulation lengths, varying numbers of blood products or infusion rates, and/or different compositions MTP. In this study, a simulated trial comparing WB versus CCT revealed differences in cumulative exposure to critical physiologic thresholds, particularly in ATLS Class IV patients (>40% blood volume lost). LTOWB recipients spent less time with hemoglobin <8 g/dL, fibrinogen <150 mg/dL, INR ≥1.5, and platelets <50×10^9^/L, despite equivalent end-of-simulation values across groups. This finding is difficult to capture in traditional clinical trials, where real-time measurement of dynamic physiologic states is often not feasible.

Our trial-simulation framework can be applied to support real-world clinical trial design. For instance, it can be used to refine inclusion and exclusion criteria, identify patients most likely to benefit from early WB therapy, or estimate sample size requirements for hard-to-capture endpoints. It enables rapid iteration over logistical scenarios, such as transfusion timing^102^, blood product shortages^103^, or delays in surgical hemostasis. By simulating the “what-if” conditions that are challenging to explore in prospective trials, this platform complements real-world evidence with biologically grounded projections.

In settings where clinical trial data are limited or delayed, this model can also serve as a data augmentation tool enabling hypothesis generation and mechanistic inference. For example, real-world trauma registries often lack granularity on oxygenation dynamics, the sequence of product transfusion, or intra-compartment fluid shifts and this simulation can map unmeasured parameters onto known clinical inputs. As in our simulation of the PAMPer trial, real world inclusion/exclusion criteria can be modeled allowing for data augmentation, particularly in the prehospital setting where data may be limited. Rather than serving as a direct replication of trial outcomes, our analysis demonstrates how a physiology-based digital twin can emulate clinical trial structure, interrogate mechanistic drivers of observed effects, and enable evaluation of physiologic endpoints that are not readily captured in conventional trials. Similarly, the model can be used to explore futility thresholds^104^, for example, estimating the likelihood of recovery when a patient reaches a hemoglobin of 3 g/dL with ongoing bleeding and no available platelets. This could support decision-making frameworks in resource-constrained or mass-casualty environments^105^. Extending this concept, the model has potential utility as an operational twin for trauma systems. By simulating transfusion product use across hundreds of stochastic trauma cases, blood banks can project demand under different MTP ratios, case mixes, and supply constraints. It can be used to stress-test the resilience of trauma protocols to shifts in blood availability, enabling data-driven policy development.

Despite its capabilities, our model has notable limitations. First, several key physiologic parameters, such as fibrinogen consumption rates, trauma-induced platelet dysfunction, and oxygen diffusion coefficients, were extrapolated or manually tuned due to the absence of robust empirical data. These values may vary across individuals and clinical states, introducing uncertainty into projections. Second, while we have included concentrated fibrinogen products such as freeze-dried fibrinogen concentrate or cryoprecipitate^106^, which are increasingly used in real-world resuscitation, we have not yet evaluated these products in the simulations. Third, sub-hemostatic concentrations of coagulation factors were not modeled as modifiers of bleeding rate, and the effects of anesthesia, acidosis, hypothermia, and inflammation^107^ were not included, which limited interpretations of our PAMPer trial simulation, whereby pre-hospital plasma administration is thought to limit coagulopathy in the pre-hospital setting. These could influence both the pathophysiology and response to treatment in ways not captured by the current architecture. In addition, while mortality was defined using cardiac index, hemoglobin thresholds, and fluid overload criteria, it is possible that patients classified as non-survivors in the simulation may have been salvageable with additional interventions not modeled here (e.g., vasopressors, mechanical ventilation, or surgical control). The model also assumes uniform injury mechanisms and excludes non-hemorrhagic causes of shock, such as neurogenic or septic processes, which may affect transfusion response in mixed-pathology cohorts although potentially at time points that are beyond the scope of this model. ${\text{VO}}_{2}/{\text{DO}}_{2}$ dependency may not exist globally in all critically ill patients (e.g. sepsis or ARDS)^108,109^. Similarly, blood lactate is not sufficient to confirm ${\text{VO}}_{2}/{\text{DO}}_{2}$ dependency and elevated blood lactate concentrations could reflect altered lactate clearance rather than anaerobic metabolism which requires clinical correlation^110,111^. Future directions include integrating laboratory-based platelet function assays, trauma-specific viscoelastic parameters^112^, and cryoprecipitate dosing schemes to better approximate modern trauma resuscitation protocols. Expansion to include post-resuscitation outcomes (e.g., multi-organ dysfunction, ICU length of stay) would also enable a broader evaluation of long-term therapeutic strategies.

In summary, this study introduces a versatile, multi-module, stochastic simulation platform that unifies key physiologic domains to enable *in silico* trauma research. The model functions as a starting point for the development of next-generation digital twins for patient-level precision and an operational twin for system-level planning, offering applications in trial design, resource allocation, protocol evaluation, and mechanistic discovery. By encoding biologically grounded constraints, latent state dynamics, and causal relationships, this framework provides a foundation for integrating AI into physiology-constrained digital twin frameworks, enabling a shift from associative to causally informed models in acute care settings.

## Methods

### In silico model description, assumptions, and initial parameters

Our model was based on the multicompartment dynamic deterministic model of a massively bleeding adult trauma patient^19,69^ integrated with a cardiovascular and tissue oxygenation model^71,72^ and a capillary exchange model^68^. Our model was implemented in MATLAB version r2023a^113^. ChatGPT-4^114^ was used to assist in code debugging, optimization, and conversion between MATLAB and R. We have manually reviewed all code suggestions and edited as needed for clarity.

The model consisted of several interconnected modules: a cardiovascular hemodynamic module, a capillary solute exchange module, a hemostatic module, tissue and alveolar capillary oxygenation modules, and a resuscitation module (Figure 1A, Figure S11). The cardiovascular hemodynamic module was based on previously described models^67,71^ and accounts for the distribution of blood to vital and non-vital organs, and pressure-volume relationships in the cardiovascular system. Fluid exchange in the interstitial space was modified to include a capillary model based on the Starling’s principle accounting for lymphatic flow driven by tissue pressure and protein washout by lymph^68^.

The model was divided into 6 phases: an initial equilibration phase (minutes 1–10), an initial injury phase (minutes 11–20), a prehospital resuscitation phase (minutes 21–40), an emergency department (ED) resuscitation phase (minutes 41–70), and operating room (OR) resuscitation phase (minutes 71–180), and a recovery phase (minutes 181–250). The equilibration phase allowed steady state to be achieved based on initial assumptions. The length of the initial injury phase and prehospital resuscitation phase were based on data from a randomized trial conducted at a Level 1 US civilian trauma center^115^ and clinical experience. The bleeding rate was held constant for 5 minutes post-injury during the initial bleeding phase and surgical hemostasis was set at 100 minutes after the patient was transferred to the OR, i.e. minute 170. Event-free survival was achieved if the cardiac index remained above 1.5^116^, fluid overload was less than 10% or SBP less than 150% of the starting SBP (see below), or if critical anemia was avoided and hemoglobin remained above 3.0 g/dL^117–120^.

### Cardiovascular module

#### Module Overview

Briefly, the cardiovascular module consisted of the heart, aorta, seven vascular beds (coronary, head, upper limbs, celiac and superior mesenteric, renal, inferior mesenteric and iliac), a combined systemic venous system, and a three part pulmonary circulation (pulmonary artery, pulmonary vein, and lungs). The load, flow, and cardiac output of the circulatory system^71^ as well as the pressure at the capillary beds^68^ determine the fluid transfer rate between the intravascular and interstitial compartments (see calculation of oncotic pressure below).

The heart consisted of four compliant chambers with four resistive unidirectional valves. Each heart chamber is modeled as a series RC circuit with the capacitance and the resistance representing the chamber’s time-variant compliance and resistance to blood flow. This allows for the calculation of the pressure, flow, and volume of each chamber during the cardiac cycle (example of pressure-volume curve of the left ventricle in Figure 2C). The cardiac valves were implemented as pressure-gated resistive unidirectional elements (mitral, tricuspid, aortic, pulmonary), as in Siam et al. 2014, using a valve switch and $R_{\text{valve}}$ to permit forward flow with a finite pressure drop and to suppress backflow; compared with the published implementation, we tightened the forward-only constraint to ensure numerical stability under our time-varying heart-rate and timestep scheme. The descriptions of the variables and equations used in this module are outlined in Table S8 and in the descriptions below.

#### Stochastic Chamber Volumes

To introduce stochastic variability, several modifications were applied to the cardiovascular module. Baseline cardiac chamber sizes were computed from published regression equations that incorporate sex, age, height, and weight^121^. Because right-ventricular volume is not routinely quantified by 2D echocardiography, right-ventricular end-diastolic volume was approximated using an area–length method from the apical four-chamber view, with end-diastolic right-ventricular area and long-axis length; single-diameter measures were used only as plausibility checks^121,122^. Age-related changes in arterial load were represented using slopes of systolic blood pressure (SBP) versus age from the Framingham Heart Study “optimal” cohort, stratified by sex^123^. At the end of equilibration, elastances of the aortic segments and branches (and, where specified, other compliant elements) were scaled to achieve an age-adjusted target SBP defined as:

$$SBP_{\text{corrected}}=SBP+\text{slope}\cdot (\text{Age}-30)$$

Finally, to avoid identical hemodynamic states among individuals with the same age, sex, and weight (and therefore the same calculated blood volume and initial SBP), the calculated blood volume was perturbed uniformly within ±2.5% prior to equilibration.

Additionally, to account for mobilization of venous blood during acute hemorrhage, the model assumed baroreflex-mediated recruitment of unstressed to stressed venous volume (mobilization. Venous compliance was treated as constant as a modeling simplification, although physiologically venous capacitance is reflex-controlled and can change with tone^124^. The baseline stressed venous volume $V_{0}$ was set to 31.25% of calculated blood volume (1,750 mL of 5,600 mL), in line with estimates that stressed volume is ~25–30% of total blood volume^125–127^. Depending on blood-volume randomization, initial $V_{0}$ values were capped at 30% of calculated blood volume. Because near-maximal sympathetic venoconstriction can recruit on the order of ~18 mL/kg of unstressed volume^128,129^, $V_{0}$ was updated to track the fraction of blood-volume lost during the simulation, with a floor of 10% of the starting calculated blood volume.


(1) $$R_{\text{SystemicVein}}=R_{\text{SystemicVein}}(1)\cdot \frac{\text{BloodVolume}(\text{cycle})}{\text{BloodVolume}(1)}$$

#### Chamber Pressure

Several key modifications were made to previous models^71^, which used the following equations to model systolic and diastolic pressure of the heart chambers:

##### Systolic Pressure


(2) $$P_{\text{syst},x}(t)=E_{max,x}\cdot E_{n,c}(tn)\cdot \left(V_{x}(t)-V_{x,0}\right)$$

##### Diastolic Pressure

###### Left Ventricle:


(3) $$P_{\text{dia},x}(t)=0.25\cdot 10^{-7.5}\cdot D_{2,x}\cdot e^{\left(D_{3,x}\cdot 6.1\cdot V_{x}(t)\right)}+7\cdot \text{ln}\;\left(D_{3,x}\cdot V_{x}(t)\right)$$

###### Atria and Right Ventricle:


(4) $$P_{\text{dia},x}(t)=D_{1,x}\cdot 10^{D_{2,x}}\cdot e^{\left(D_{2,x}\cdot V_{x}(t)\right)}+D_{3,x}\cdot \text{ln}\;\left(D_{4,x}\cdot V_{x}(t)\right)$$

##### Chamber Pressure


(5) $$P_{x}(t)=max\left(P_{\text{sys},x}(t),P_{\text{dia},x}(t)\right)$$

With the overall pressure of a heart chamber, ${\text{P}}_{\text{x}}(\text{t})$, given by equation (5) and with x representing a specific heart chamber.

However, these equations were modified such that unidirectional flow **must** be maintained and that a volume of a heart chamber, $V_{i,x(t)}$, **must** be greater than or equal to the unfilled volume of a particular heart chamber (i), $V_{o,i},V_{i,x(t)},V_{o,i}V_{i,x(t)}$ at a time t (given as 1/N where N is the number of iterations, dt, within 1 second). ${\text{P}}_{\text{diastolic}}$ was also updated to match as originally written along with $D_{i}$ updated (Table S8) to specifically match the values described in (Siam 2014).

##### Systolic Pressure


(2’) $$P_{\text{syst},x(t)}=max\left(E_{max,x}\cdot E_{n,c}(tn)\cdot V_{x(t)}-V_{x,0},0\right)$$

##### Diastolic Pressure


(3’) $$P_{\text{dia},x(t)}=D_{1,x}\cdot 10^{\left(D_{2,x}\right)}\cdot e^{\left(D_{2,x}\cdot V_{t}(t)\right)}+D_{3,x}\cdot \text{ln}\;\left(D_{4,x}\cdot V_{x}(t)\right)$$

(6) $$V_{i,t}=V_{i,t-1}+\left(Q_{\text{in}}-Q_{i,t-1}\right)\cdot dt$$

(7) $$V_{i,t}=max\left(V_{i,t},V_{o,i}\right)$$

Notably, the diastolic pressure of the heart chambers follows an exponential curve at higher volumes^130,131^, consistent with the model equations for ${\text{P}}_{\text{dia}}$ (equation 2 & 2’). However, following resuscitation, in some patients, heart chamber flow and heart chamber volume in the left atrium and ventricle substantially increased due to fluid pooling in the low compliance lung chambers causing increases in pulmonary flow (${\text{Q}}_{\text{pulm}}$). Small volume changes in the left ventricle led to dramatic increases in left ventricle diastolic pressure and chamber pressure inconsistent with systolic pressure. Therefore, **pulmonary blood flow was limited to a maximum of ~120mL/sec**, as the maximum cardiac output in a typical adult is in the range of 18.5 L/min and 24.1 L/min for men^132,133^, though cardiac output can be higher in elite athletes^134,135^. The expected tracings for systole, diastole, and overall chamber pressure were maintained upon implementing these changes (Figure S1B).

#### Chamber Elastance and Contractility

Variations in stroke volume are due to cardiac muscle contractility. ${\text{E}}_{\text{max}}$, the contractility index^67^ or the maximum slope of the instantaneous pressure-volume curve^136^ was not modified throughout the simulation. ${\text{E}}_{\text{max}}$ was stochastically randomized (Figure S7U) with a mean of 2.0 mmHg/mL^67^ and a standard deviation of 0.6 derived from an animal study^136^.

The ejection pressure of each heart chamber is calculated using a modified Suga-Sagawa relation^136–138^ describing the relation of pressure, flow, and volume in a heart chamber^67^. This pressure is proportional to the normalized chamber elastance function to ${\text{E}}_{\text{max}}$ in which the time variable has been normalized such that: $t_{n}=\frac{t}{t_{\text{max}}}$ where t is the current time in the heart cycle, ${\text{t}}_{\text{max}}$ is the heart cycle period, and ${\text{t}}_{\text{N}}$ is the chamber’s dimensionless elastance parameter^67,71^. Additionally, the normalized elastance curve of each heart chamber was adapted from equation (8)^71^ to equation (8’) to match what was first described^67^ from:

(8) $$E_{\text{nor}}=max\left(\left(E_{n}(3)\cdot t_{n}\right)+\left(E_{n}(2)\cdot t_{n}\right)+\left(E_{n}(1)\cdot t_{n}\right),0\right)$$

to:

(8’) $$E_{\text{nor}}=max\left(\left(E_{n}(3)\cdot t_{n}^{3}\right)+\left(E_{n}(2)\cdot t_{n}^{2}\right)+\left(E_{n}(1)\cdot t_{n}\right),0\right)$$

with En values set to what is outlined in Table S8^67^, as opposed to **all four** heart chambers having the same elastance of [0.158, 2.685, −1.841] (Figure S1A).

#### Time Varying Heart Rates

Additionally, this model introduced a time-varying heart rate (HR) and a stochastic starting HR (see below). This required modifications to several aspects of the cardiovascular module. During initial equilibration (120 cycles), the HR was set to 80 beats per minute (bpm), consistent with prior models^71^. With a static HR, the number of beats per minute is constant, and the timing of systole and diastole remains fixed with respect to the integration time step dt within one “second”/cycle (Figure S12A, S12B). Because the heart cycle was constant in wall-clock time, the original simulation advanced using a fixed number of iterations per cardiac cycle (Siam, Mandel, and Barnea 2014, 201).

In the constant-HR scheme of Siam, Mandel, and Barnea^71^, the integration step is made independent of HR and fixed as:

$$\frac{60\;\text{seconds}}{80\;\text{beat}}*\frac{1}{N}$$

i.e., N iterations for 0.75 seconds (1 beat). In the present model, the integration step is made independent of HR and fixed as dt=1/N (s per simulation second), and the beat period is handled explicitly via the cycle length

$$\text{secperbeat}=\frac{60\;sec}{1\;min}\cdot \frac{1}{HR(\text{beats})}\;\text{tmax}(\text{cycle}+1)=\text{round}\left(\frac{\text{sec}}{\text{beat}}\cdot \frac{N\;\text{iterations}}{1\;\text{sec}}\right),$$

so ${\text{t}}_{\text{max}}$ (samples per beat) varies with HR.

The heart cycle period (${\text{t}}_{\text{max}}$) and the “delay” for which a contraction signal moves from the sinoatrial node to the atrioventricular node was originally given as:

(9) $$t_{max}=\text{round}\left(\frac{400-1.8\cdot HR}{1000\cdot dt}\right)$$

(10) $$\text{delay}(1:2)=\text{round}\left(\frac{0.160}{dt}\right)=\text{round}\left(0.160\cdot \frac{80\frac{\text{beats}}{min}\cdot N}{60\frac{sec}{min}}\right)$$

with the heart rate, HR, set to a constant 80 bpm, and $dt=\frac{\frac{60}{HR}}{N}$. With changes to dt and the time varying HR, the delay between SA and AV node was changed to equation (10’).


(10’) delay(1:2)=round(-0.351⋅HR(cycle)+176.7)

The minimum allowable HR for a patient was set at 50 bpm and the maximum allowed HR followed an age-based limit^139,140^.

With a varying HR, the heart cycle period ${\text{t}}_{\text{max}}$ is no longer forced to equal N (the number of iterations within one cycle/second). Hence, ${\text{t}}_{\text{max}}$ becomes desynchronized from the fixed integration step dt (Figure S12B). For example, with N = 500: at 60 bpm, one beat spans ~1.0 cycles/s and $t_{\text{max}}=500$; at 120 bpm, one beat spans ~0.5 cycles/s and $t_{\text{max}}=250$, which is less than the number of iterations N in one second (N=500). Similarly, with N = 500, at 50 bpm, $t_{\text{max}}=600$ which is beyond the number of iterations N in one second (N=500). To manage this, a third time variable $t_{\text{heartcycle}}$ was introduced to track phase within the cardiac cycle; it advances each iteration and resets at the end of left-ventricular contraction (the final chamber to contract) (Figure S1C–E). As HR increases, reduced diastolic filling time produces the expected reduction in end-diastolic volumes and stroke volumes.

### Fluid compartment module

#### Module Overview

Similar to the multicompartment deterministic model of a massively bleeding adult^19^, our model included compartments for the total body water (TBW), intracellular fluid (ICF), RBC compartment volume (RBCF), non-RBC intracellular fluid (NRBCF), extracellular fluid (ECF), plasma volume (PV), and interstitial fluid (ISF) volume^141,142^. The initial values for these parameters, as well the equations used to derive them are shown in Table S7. A separate functional plasma compartment (PC) calculated from subtracting the PV from the infused non-oxygen carrying, non-hemostatic fluids and the free water that entered the plasma compartments from the ISF and RBC compartments was used to calculate the plasma dilution factor (PC/PV). The RBCF, PV and PC loss was determined by the bleeding rate and the consumption rate. RBC transfusion replenished the volume and osmolar content of the RBCF compartment, while crystalloid, plasma and PLT infusion replenished the volume and osmolar content of the PV compartment (for crystalloid, plasma and PLT) and PC compartment (for plasma and PLT).

This model accounted for changes in osmolarity and fluid volumes in the various compartments based on (1) the osmolarity gradients between compartments, (2) the metabolism of infused dextrose, (3) the diffusion of free water based on Starling’s law for transcapillary pressure gradient, (4) solute-solvent coupling, whereby solute transfer from the PV to the ISF was accompanied by free water movement^143^, (5) transfer of protein and fluids into the plasma compartment from the ISF via the lymphatic circulation^68^, and (6) oxygen content and partial oxygen pressure at each segment as a proxy for tissue oxygenation^72^. Additional details about the modeled changes in osmolarity and fluid volumes in the body compartments are outlined below in the oncotic pressure subsection.

#### Compartment Volumes

The volumes of the cardiovascular module were integrated with the fluid compartment module through the calculation of the total venous volume, which was calculated as

(11) $$V_{\text{vein}}=V_{\text{total}}-\left(V_{\text{heart}}+V_{\text{segment}}+V_{\text{pulm}}+V_{\text{branch}}\right)$$

where ${\text{V}}_{\text{total}}$ is the sum of the RBC compartment and total PV compartment, which included any non-oxygen carrying, nonhemostatic fluids (redistributed body water, crystalloid, CPD, and AS)^19^. Notably, a “third spacing” variable was introduced to approximate fluid transferred from the pulmonary capillaries into the ISF in which changes in pulmonary pressure (compared to the equilibration baseline) leads to a percentage change in volume according to prior studies in animals (Guyton 1965) with a corresponding decrease in the plasma compartment. The “third spacing” volume is scaled to transfer <100ml into the ISF without any resuscitation or bleeding (Figure S13, S14).

Furthermore, to account for stochastic patient characteristics, the total body water was adjusted according to the Hume equation (Hume and Weyers 1971). Patients were also considered fluid overloaded based on a calculation of their percent fluid overload, given by $\%\text{FluidOverload}=100\cdot \frac{{\text{total}}_{\text{in}}-{\text{total}}_{\text{out}}}{\text{weight}}$ or an increase in their SBP of greater than 50% of their starting SBP. Percent fluid overloads greater than 10% are correlated with an increased mortality^88^ and SBP >200 is correlated with an increased incidence of admission to the intensive care unit or death within 60 days from the modified early warning (MEWS) score^144^.

This model accounted for refilling of the PV and plasma compartments with infused fluid, which contains oncotically-active proteins and coagulation factors. The osmolarity of all body compartments was initially assumed to be 0.3 mOsm/mL before compartment equilibration. The increase in fluid compartment osmoles was calculated by multiplying the volume of the infused crystalloid or blood product multiplied by the osmolarity of the component infused. For the lymphatic system, coagulation factor activities vary from 20% to 70% of their plasma concentrations^145^, and plasma protein concentrations in lymphatic fluid are about 20–65% of their plasma concentrations^146^. However, we assumed lymphatic fluid contained an equivalent plasma protein concentration and coagulation factor activity to plasma to account for continuous hepatic production of plasma proteins and coagulation factors. Additionally, as this model was set at ~240 minute run time, the elimination half-life of infused fluids was not included in the model since this is likely to be greater than 240 minutes in trauma patients^147^ and unlikely to lead to significant contributions to compartment volumes due to renal hypoperfusion and reduced renal filtering^148^.

This module also accounted for the metabolism of the infused dextrose that is contained in the CPD (citrate phosphate dextrose) and AS-1 solutions. The calculated dextrose osmolar content for CPD was 9.0 mOsmoles in 70 mL and 12.2 mOsmoles in 110 mL of AS-1. This was based on the dextrose content in both solutions and the molecular weight of dextrose monohydrate (198 g/mol)^149^. Dextrose was metabolized according to first-order kinetics with a metabolism half-life of 16 minutes^150^ if the total PV or ISF dextrose osmolar content exceeded the initial dextrose osmolar content. This led to a corresponding decrease in the osmolar content of that compartment.

#### Calculation of Oncotic Pressure

Fluid exchange between the cardiovascular and interstitial compartments followed the Starling equation (Table S9)^151^. Throughout the length of non-fenestrated capillaries fluid is filtered to the ISF and the filtered fluid returns to the circulation as lymph^68,152,153^.

The net filtration rate is modeled as:

(12) $$NFR(t)=K\cdot \left[\left(P_{cap}-P_{ISF}\right)-\left(\pi_{cap}-\pi_{onc}\right)\right]$$

where K (mL/ms/mmHg/mm) is the endothelial hydraulic conductance, which is a measure of the capacity of the capillary membrane to filter water per unit capillary length, ${\text{P}}_{\text{cap}}$ and ${\text{P}}_{\text{ISF}}$ are the hydrostatic pressure exerted by the capillary and ISF respectively, and $\pi_{\text{cap}}$ and $\pi_{\text{ISF}}$ are the oncotic pressure exerted by the capillary and ISF respectively. K is estimated by assuming 8 mmHg of filtration pressure leads to 1/200 of plasma in flowing blood to filter out of the arterial ends of the capillary (assumed to be L/2) and into the interstitial space^68^. In contrast to previous models^68^, the Hct and velocity of the capillary blood flow (${\text{Q}}_{\text{c}}$ per Siam, Mandel, and Barnea^71^) fluctuate throughout the simulation and capillary blood velocity changes as a function of the heart module’s average branch pressure. Several methods to calculate the hydrostatic pressure across the capillary were explored, including $P_{\text{cap}}=J_{a}+J_{v}$^70,71^, and the Papenheimer-Soto-Rivera equation which uses ratios of the precapillary and postcapillary resistances to blood flow resistance to determine mean capillary pressure^154^. However, the hydrostatic pressure along the capillary (${\text{P}}_{\text{pl}}$; mmHg) was modeled as a linear function of axial distance of the ith compartment, ${\text{x}}_{\text{i}}$ (mm), along the capillary according to $P_{pl}\left(x_{i}\right)=P_{pl,a}+\left(P_{pl,v}-P_{pl,a}\right)\cdot \frac{X_{i}}{L}$ where ${\text{P}}_{\text{pl},\text{a}}$ is the plasma hydrostatic pressure at the arterial end and ${\text{P}}_{\text{pl},\text{v}}$ is the plasma hydrostatic pressure at the venous end of the capillary^68^.

Previous models^71^ assumed the concentration of colloids in the interstitial space is constant and ignored the return of lymph. Changes in the blood oncotic pressure with the concentration of colloids were modeled according to the equation $\pi =0.02274C^{2}+2.1755C$, with the change of blood protein concentration during hemorrhage and fluid resuscitation assumed to be proportional to HCT according to ${\text{Ccol}}_{B}\left(t\right)={\text{Ccol}}_{B}\text{norm}\cdot \frac{Hct\left(t\right)}{Hct_{\text{norm}}}\text{ADDIN ZOTERO\_ITEM CSL\_CITATION}\{\text{citationID}:27\text{e}9\;\text{wJ}\;2\;\text{M}$,^71^. Our model accounted for changes in the plasma compartment and ISF protein concentration largely according to the methods outlined in Himeno *et al*.^68^ with the parameters and equations outlined in Table S9. Briefly, the change in the amount of plasma protein from filtration at the capillary was modeled according to $\frac{\partial q_{pl}\left(t,x_{i}\right)}{\partial dt}=-\frac{q_{qpl}\left(t,x_{i}\right)-q_{pl}\left(t,x_{i-1}\right)}{\Delta x}*v_{pl}-j_{q,C}$ and the change in ISF protein at the capillary was modeled with $\frac{\partial q_{ISF}(t)}{\partial dt}=J_{q,C}(t)-J_{q,LF}(t)$. However, we expanded upon this model to account for colloids lost due to bleeding by including

$${Col}_{\text{loss}}(t)=J_{\text{bleed}}(t-1)*\frac{{\text{Colloid}}_{\text{Compartment}}(t-1)}{\text{RBCF}(t-1)+V_{pl}(t-1)}$$

and accounting for colloids gained from transfusions through stochastically selecting blood products with a colloid concentration between 60 and 80 mg/mL^155^.

Additionally, in this module several key assumptions were made about the boundary conditions which were not previously described (Figure S12C).

**1.** The change in the volume of the first segment of the capillary ($\delta Vpl_{\text{axial}}(1,\text{t})/\delta \text{t}$) assumes that the volume of the boundary condition ${Vpl}_{\text{axial}}(\text{x}-1,\text{t})$ is equal to the total volume of the capillary from the previous cycle divided by the number of capillary segments: $\frac{\Sigma Vpl_{\text{axial}}\left(x_{i},t-1\right)}{\Delta x}$.

**2.** The protein concentration of the first segment of the capillary (${\text{q}}_{pl}(1,\text{t})$) is equal to $q_{pl}(1,t)=v_{pl}(1,t-1)*c_{pl}(t)$, where ${\text{c}}_{\text{pl}}(\text{t})$ is the colloid concentration of plasma that changes due to bleeding and/or resuscitation.

**3.** The calculations for oncotic pressure were calculated for a single “average” capillary. However, given the global volume changes that occur during bleeding and resuscitation, we estimated the number of capillaries as a way of calculating global NFR and ${\text{J}}_{\text{lymph}}$. The number of capillaries (${\text{N}}_{\text{cap}}$) was estimated through approximations of the flow rate and area (Q=AV) between the aorta and a single capillary (equation (13)) although capillary density varies based on muscle location or tissue^156,157^.


(13) $$N_{cap}=\frac{\left(0.012m{)}^{2}*(0.4m/s\right)}{\left(0.003m/s{)}^{2}*(7e-7m\right)}$$

#### Hemostatic module

The hemostatic module simulated changes in bleeding rate as a function of systolic blood pressure that occurs with ongoing hemorrhage. Each patient is randomly assigned an Advanced Trauma Life Support (ATLS) class based on the average distribution of ATLS class at presentation to the ED categorized by shock index or heart rate^76^ such that about 74% and 26% of patients present as ATLS Class III and class IV, respectively. ATLS class I or II patients are modeled and can be selected per reported distributions, but were not included in this study due to the more limited need for blood transfusion in the resuscitation of these patients.

Following class randomization, a “hidden bleed” parameter is then stochastically chosen based on the mortality rate of each ATLS class^158^. The “hidden bleed” parameter is a modified version of the hemostatic factor^69^ that simulates hemorrhage that continues to bleed even at decreased SBPs despite efforts at tamponade. Similarly, trauma induced coagulopathy (TIC) is a possible cause of early post-injury mortality that was first described in a case series of patients with major abdominal vascular injuries with bleeding-related deaths despite mechanical control^159^. The incidence of TIC diagnosed via laboratory tests varies, but most studies converge at an incidence of about 25% of severely injured patients with an associated 35–50% mortality^82^. As such, approximately 5% of ATLS class III and 25% of ATLS class IV were patients randomly assigned to higher consumption values for RBCs, plasma, fibrinogen, and platelets to simulate these patients developing TIC with a higher time spent in critical intervals.

Following model equilibration (minutes: 1–10), injury begins at time 11 minutes with an initial bleeding rate stochastically defined based on the ATLS class of trauma (Table S3) (American College of Surgeon Committee on Trauma 2018). The reported blood volume loss is <15%, 15–30%, 30–40%, and >40% for ATLS class I, II, III, and IV^10^. Using the patient’s calculated blood volume and randomly selected ATLS class, a bleeding rate is calculated such that at a constant bleeding rate, they would lose a volume of blood equal to a randomly selected percent of blood volume within their respective ATLS class within 30 minutes of bleeding. For example, if a patient with a calculated blood volume of 5600ml was randomized to ATLS class IV with a percent blood loss of 45%, then the bleeding rate was set to ~1.4 ml/sec such that the patient would lose approximately 2500 ml in 30 minutes. We were unable to find a reference for the time frame for which these percentage of blood volumes were lost, and 30 minutes was chosen to approximately match the mortality rates when patients did not receive massive transfusion protocols.

As all patients shared the hemostatic factor correction term, to prevent convergence of the patients’ SBPs, the initial bleeding rate is held constant for the next 5 minutes in the field. Then, the bleeding rate varies with changes in the SBP for the next 160 minutes. At t=160 minutes, surgical hemostasis is achieved and the bleeding rate falls over the next 20 minutes based on an exponential equation approximating hemostasis with an updated geometric decrease in bleeding rate during the recovery phase simulating a continuous, slowly oozing bleeding rate^19^.

Similar to previous models^69^, the following assumptions were made: the bleeding was arterial, the sympathetic response did not account for the effects of pain or drugs, the bleeding rate was not affected by the platelet count, fibrinogen concentration, hematocrit, or international normalized ratio (INR), blood viscosity and capillary radius remained constant, and the vascular defect remained constant over time until surgical hemostasis is achieved.

Unlike previous models^19^, the starting SBP during this simulation is now derived from the cardiovascular module as the maximum pressure in each heart cycle of the left ventricle (as opposed to the first aortic segment^71^).

#### Resuscitation module

The composition of the blood products transfused during each phase of the simulations for the various resuscitation strategies are shown in Table S6. Several new resuscitation strategies were implemented to capture a variety of resuscitation strategies and real-world scenarios. However, the basic structure of each resuscitation protocol was kept largely the same as previously published models^19^.

The following assumptions were made about the composition of transfused blood components^19^: A unit of WB consisted of hemoglobin-containing RBCs^160^, fibrinogen- and coagulation-factor containing plasma with a defined INR, platelets and non-hemostatic, non-oxygen carrying CPD solution. A unit of red blood cells did not contain fibrinogen or platelets, while plasma did not contain hemoglobin, and platelets did not contain hemoglobin or fibrinogen. Additionally, in our model each blood product included a defined concentration of plasma proteins (mg/ml). However, to account for differences among blood products, each parameter of each blood product (WB, RBC, plasma, PLT) given was randomized with the following steps taken to ensure covariation of RBC and plasma volumes.

First, each unit was assumed to be derived from a 500ml donation of whole blood (${\text{V}}_{\text{Donor}}$). The variances in rbc volume, plasma volume, and platelet volume from a level I trauma center (unpublished data from University of Pittsburgh Medical Center) per Table S6 were used to calculate the variance in total donation volume (± ~22 ml). Then, a stochastic hematocrit for each “donor” (${\text{Hct}}_{\text{Donor}}$) was used to calculate the total red cell volume (${\text{V}}_{\text{RBC}}$) and plasma volume (${\text{V}}_{\text{Plasma}}$) per:

(15) $$V_{RBC}=V_{\text{Donor}}\cdot Hct_{\text{Donor}}$$

(16) $$V_{\text{Plasma}}=V_{\text{Donor}}*\left(1-Hct_{\text{Donor}}\right)$$

The total hematocrit of an RBC unit was then stochastically chosen (Zuck et al. 1977) and a total volume of plasma and CPD (${\text{V}}_{\text{CPD+plasma}}$) in a red cell was calculated. We assumed a fixed total volume of 70ml from the manufacturer and assumed a fixed fractionation value of 20% to calculate how much CPD constitutes ${\text{V}}_{\text{CPD+plasma}}$ per:

(17) $$Hct_{RBC}=\frac{V_{RBC}}{V_{RBC}+V_{CPD+\text{plasma}}}$$

where $V_{CPD}=0.2\cdot V_{CPD+\text{Plasma}}$ and $V_{RBC_{\text{Plasma}}}=0.8\cdot V_{CPD+\text{Plasma}}$. The total volume of a plasma unit (${\text{V}}_{\text{PlasmaUnit}}$) was then calculated from ${\text{V}}_{\text{Plasma}}$ according to:

(18) $$V_{\text{PlasmaUnit}}=V_{\text{Plasma}}-V_{RBC_{\text{Plasma}}}-V_{\text{Platelet}+CPD}$$

where ${\text{V}}_{\text{Platelet}}$ is the volume of a platelet unit derived from the plasma of a whole blood unit with a mean volume of 69.3 ml. Again, a fixed fractionation value of 20% was assumed for how much CPD is in each unit of platelets, $V_{\text{CPD}}=0.2*V_{\text{Platelet}+CPD}$ and $V_{\text{Platelet}}=0.8*V_{\text{Platelet}+CPD}$. Then the volume of CPD in a plasma unit was calculated as:

(19) $$CPD_{\text{PlasmaUnit}}=CPD_{\text{total}}-V_{CPD_{RBC}}-V_{CPD_{\text{plateler}}}=70-V_{CPD_{RBC}}-V_{CPD_{\text{placelet}}}$$

As described above, we also accounted for colloids gained from whole blood, plasma, or platelet transfusions through stochastically selecting blood products with a colloid concentration between 6 and 8 g/dL (Barrett et al. 2010) which is then diluted with CPD.

Our conventional component therapy resuscitation strategy was modified to allow a user to select a customizable resuscitation strategy. During massive transfusion, protocols known as massive transfusion protocols (MTP) are used to standardize treatment response and preemptively respond to the acidosis, hypothermia, and coagulopathy that can occur from reactive, supportive treatment of patients with massive hemorrhage (Patil and Shetmahajan 2014). As such, an “MTP Resuscitation” strategy was given in which 1L of crystalloid is initially given along with 3 units of WB in the ED. Then, 1 MTP packet consisting of RBCs, plasma, and platelets using a 6:6:1 ratio of RBC:plasma:platelets is given in the OR. This resuscitation strategy was modified such that the composition of an MTP packet, the number of WB vs. MTP packets, and the speed at which any unit can be given could be modified by a user. While rapid infusers can achieve higher flow rates and infuse a unit within ≈1–2 minutes^89,90^, infusion rates were set at specific rates depending on the phase and the overall resuscitation strategy was designed to be modifiable depending on the user’s need. Our simulation results uses the previous model's (Seheult et al. 2019) value of 12, 8, and 12 minutes to administer one blood product in the prehospital phase, ED phase, and recovery phase respectively, all with a 2 minute pause to exchange blood products. Similarly, 6 minutes are required to administer one blood product in the OR with a one minute pause to exchange blood products.

For a proof of concept comparison between WB vs. CCT blood products, a single simulated clinical trial was completed with the resuscitation strategy outlined in Figure 4, Figure S4. Briefly, 100 stochastic patients were initialized and set as either ATLS class III or ATLS IV. These patients received 1 L of saline in the prehospital setting. Then if the patients were class III they received 6 units of either WB or CCT (3 in the ED, 3 in the OR). If the patients were designated as ATLS class IV, they received 12 units of WB or CCT (3 units in the ED, 9 in the OR). Median values of the time in critical intervals (described below) or the median of a final value (i.e. SBP at the end of the simulation) were considered statistically different with p values ≤ 0.05 using Welch Hammond regression.

#### Goal Directed Therapy Module

A novel module that incorporates continuous hemodynamic and hemostatic assessment (vital signs and laboratory data) of a patient to guide clinical decisions points in resuscitation was implemented. This Goal Directed Therapy (GDT) approach approximates real-time clinical decision-making and real-world trauma resuscitation practice. In this module, two initial units (either LTOWB or RBC) are given to a patient upon arrival in the ED (LTOWB shown in Figure 6). Simulated specimens are then collected for laboratory testing and laboratory results (hemoglobin, fibrinogen, INR, platelet count) return after a predefined time threshold (set to 15 minutes for this proof-of-concept simulation). The transfusion protocol is then adjusted based on the results of SBP, Hemoglobin (RBC), INR (plasma), total platelet count (platelets) if bleeding remains brisk or uncontrollable or is controlled after surgical hemostasis is achieved. Either LTOWB or CCT can be selected for GDT resuscitation (CCT shown in Figure 6). An SBP of below 80 mmHg or a Hgb of 8 or 10 g/dL triggers one unit of RBC/LTOWB and a Hgb less than 8 g/dL triggers two units of RBC/LTOWB. Similarly, if the patient has an INR of 1.6 or 2.0 they receive one unit of plasma/LTOWB and if INR is greater than 2.0, this triggers two units of plasma/LTOWB. A platelet count of less than 50 × 10^9^/L triggers one pool of platelets/LTOWB. Each threshold can be modified for a particular simulation. The speed at which one unit is transfused was set to six minutes per unit, with a one-minute pause to change bags, which is set to be easily modifiable as needed.

#### Prehospital Air Medical Plasma (PAMPer) Module

Real world clinical trial inclusion/exclusion criteria from PAMPer trial^17^ were emulated to demonstrate the ability of our model to serve as a physiology-based digital twin in a clinical trial setting. In this module, a single simulated patient was simulated both in the control and intervention arm (as opposed to two separate simulated patients for each arm).

Patients in the PAMPer trial had varying prehospital time from the first measurement of qualifying vital signs. Both patients found at the scene (median prehospital time: 39 minutes; IQR: [31,49]) and patients transferred from outside referral emergency departments (median prehospital time: 52 minutes; IQR: [40.5,70.5]) were included in the trial. Simulated patients were randomly assigned to be “transfer” patients (n = 111 in the PAMPer trial) or “scene” patients (n = 390 in the PAMPer trial) with corresponding variation in their total prehospital time. Next, as emergency medical services (EMS) arrive, all simulated patients in the standard-care arm receive 1L of crystalloid fluid, while patients in the intervention arm are evaluated for the plasma inclusion criteria. If patients in the intervention arm had an SBP ≤ 70 mmHg, or an SBP between 71–90 mmHg and HR ≥ 108, then 2 units of plasma are given. Exclusion criteria, aside from age, were unable to be modeled (e.g. known prisoner or known pregnancy). The PAMPer module was run every 1 minute of simulated time to simulate continued assessment of prehospital patients for changes in SBP or HR. If a simulated patient met inclusion criteria, they were stochastically assigned to receive 2 units of plasma (89.1%), 1 unit of plasma (9.1%), and 0 units of plasma (1.7%) to reflect the number of plasma units patients received in the trial.

Following infusion of 1L crystalloid (standard-care arm) or 2 units of plasma (intervention arm), patients were transitioned to pre-hospital goal directed therapy (GDT) in which a 500ml saline bolus or a red blood cell transfusion was given if a patient’s SBP < 90 mmHg. RBC transfusions occurred if the injured patient met additional inclusion criteria, including tachycardia with a HR > 120 beats per minute, a lactate level ≥ 4 mmol/L, or a shock index (HR/SBP) > 0.9. Additionally, an “inclusion” parameter was included to simulate patients who met the other reported RBC inclusion criteria (ex. change in mental status) or who had an RBC transfusion initiated at a referring facility. Patients who met the inclusion criteria for an RBC were then randomly chosen to receive an RBC or a 500 mL crystalloid bolus based on an “RBCvsCrystalloid” parameter. The “inclusion” and “RBCvsCrystalloid” parameters were scaled according to the 38% (scene patients) or 54% (transfer patients) of patients in the standard-care arm who received prehospital RBC transfusion.

The expected underlying mechanism responsible for a survival benefit of plasma includes a reduction in coagulopathy, reduction in endothelial injury, and improvement in hemostatic function^161–163^. However, in our model coagulation parameters do not directly affect the bleeding rate (as controlled by the hemostatic factor). Therefore, a logical loop in the prehospital setting was implemented in which a patient with an INR ≥ 2.0 or a fibrinogen ≤ 150 mg/dL was temporarily set to have the hidden bleed hemostatic factor to account for the effect of coagulopathy on bleeding. This improved module emulation as initial implementations provided a survival benefit through reduced dilutional effects because the intervention arm was not guaranteed to infuse 1L of fluid.

Additionally, to more accurately reflect real world pre-hospital settings, the speed at which one unit is transfused was stochastically set to 3–5 minutes per unit based on 16g (~180 mL/min) or 18g (~90 mL/min) needles, with a one-minute pause to change bags. The maximum number of red blood cells infused in the prehospital setting was limited to two units to simulate inventory limitations in the pre-hospital setting.

Upon arrival at the ED, all simulated patients received 2 units of RBC and then transitioned to the GDT approach (described in the Goal Directed Therapy Module subsection) using CCT. Briefly, an SBP of below 80 mmHg or a Hgb of 8 or 10 g/dL triggers one unit of RBC/LTOWB, a Hgb less than 8 g/dL triggers two units of RBC/LTOWB, an INR of 1.6 or 2.0 triggers one unit of plasma, an INR greater than 2.0 triggers two units of plasma, and a platelet count of less than 50 × 10^9^/L triggers one pool of platelets. Surgical hemostasis was deterministically achieved 100 minutes after the start of the OR phase for all patients in both the standard-care and intervention arm.

All simulations ended at minute 300 regardless of the length of time spent in the recovery phase.

#### Dilutional Coagulopathy module

The dilutional coagulopathy module was similar to our previous models^19^. The hemoglobin concentration was calculated by dividing the total quantity of circulating hemoglobin in the RBCs by the total blood volume (RBC compartment + PV compartment). The plasma compartment models endogenous coagulopathy (activated Protein C, tissue plasminogen activator). The uncorrected PLT count was calculated by dividing the total circulating pool of PLTs by the total blood volume after applying an adjustment function^69^. This corrected PLT concentrations by up to 30% of the level predicted by dilution alone, which accounts for PLT recruitment from the spleen and other sites. The prothrombin time (PT) was calculated from the plasma compartment divided by the plasma volume (% plasma dilution) versus PT from a PT-dilution curve generated using laboratory data^19^. The fibrinogen concentration was calculated by dividing the total circulating fibrinogen by the total PV compartment, which included any non-oxygen carrying, non-hemostatic fluids.

The critical interval was defined as the duration of time spent with an INR greater than 1.5^83^, fibrinogen concentration less than 150 mg/dL^164^, and a platelet count less than 50*10^9^/L^165,166^.

As in previous models^19^, RBCs, plasma (including fibrinogen), and platelets were “consumed” during clot formation, with consumed plasma converted to intravascular colloid as it becomes non-oxygen-carrying, non-hemostatic fluid to account for consumptive coagulopathy. Each consumption factor was scaled such that during infusion with a unit of whole blood over 10 minutes (in the ED), there was a net balance between the infusion and consumption of a specific parameter. However, in the stochastic model, this consumption factor was set as the maximum consumption rate, with decreasing consumption rates for each ATLS class and approximately 5% of ATLS class III and 25% of ATLS class IV patients randomly assigned to higher consumption values for plasma, fibrinogen, and platelets to simulate these patients developing TIC with higher time in critical intervals.

### Tissue oxygenation module

#### Oxygen Consumption

The tissue oxygenation module was based on a previous model of tissue oxygenation (Siam et al. 2015; Siam 2014) with some modifications. Briefly, as oxygen travels from the arterial to the venous end of the capillary (axial direction), oxygen diffuses continuously into tissue (radial direction) according to the conservation of mass equation^167^:

(20) $$\alpha_{t}\frac{\partial P_{t}}{\partial t}=\alpha_{t}D_{t}\left(\frac{\partial^{2}P_{t}}{\partial r^{2}}+\frac{1}{r}\frac{\partial P_{t}}{\partial r}\right)+\alpha_{t}D_{t}\frac{\partial^{2}P_{t}}{\partial z^{2}}-M\left(P_{t}\right)$$

where $\text{M}\left({\text{P}}_{\text{t}}\right)$ is the oxygen consumption rate of the tissue according to:

(21) $$M\left(P_{t}(z,r,t)\right)=\frac{M_{0}*P_{t}(z,r,t)}{K_{m}+P_{t}(z,r,t)}$$

where ${\text{M}}_{0}$ is the maximum consumption rate and ${\text{K}}_{\text{m}}$ is the Michaelis-Menten coefficient. With improvements in optical technique and the ability to measure ${\text{PO}}_{2}$ in living organs, ${\text{K}}_{\text{m}}$ was updated to 10.5 mmHg^168^, while ${\text{M}}_{0}$ was set to 8.1*10^−4^ mlO_2_*ml^−1^*s^−1 72^ vs. 1.391*10^−4^ mlO_2_*ml^−1^*s^−1 168^. The boundary condition was defined according to Fick’s law^167^ such that:

(22) $$⊢\left(J_{-}\{t-\text{axial}\}(z=0,r,t)=J_{-}\{t-\text{axial}\}(z=L,r,t⊣)\right.$$

(23) $$J_{\text{t-radial}}\left(z_{0},r_{0},t\right)=J_{\text{t-axial}}\left(z_{0},r_{0},t\right)=-{\left.\alpha_{t}D_{t}\frac{\delta P_{t}(z,r,t)}{\delta r}\right|}_{r=r_{0};z=z_{0}}$$

#### Capillary Oxygenation

The capillary O2 concentration along the capillary was calculated according to:

(24) $$C_{c}(z,t)=\alpha_{t}\cdot P_{c}(z,t)+HCT\cdot Hb_{RBC}\cdot O_{2Hb}\cdot SO_{2}\left(P_{c}(z,t)\right)$$

where ${\text{C}}_{\text{c}}(\text{z},\text{t})$ is the capillary ${\text{O}}_{2}$ concentration in the capillary, $\alpha_{\text{t}}$ is the blood oxygen solubility, ${\text{P}}_{\text{c}}(\text{z},\text{t})$ is the ${\text{O}}_{2}$ pressure in the capillary at axial position z and time t, HCT is the blood hematocrit, ${\text{Hb}}_{\text{RBC}}$ is the hemoglobin density (34 g/dL), ${\text{O}}_{2\text{Hb}}$ is the maximum ${\text{O}}_{2}$ concentration in hemoglobin, and ${\text{SO}}_{2}\left({\text{P}}_{\text{c}}(\text{z},\text{t})\right)$ is the oxygen-hemoglobin dissociation relation at axial position z and time t. As described previously, oxygen pressure showed a small increase at the outlet of the capillary as there was oxygen inflow from the tissue to the capillary due to the proximity of the arterial end of the next group of capillaries. However, notably a few key differences were implemented in our model vs. the previous model.

**1.** As in prior capillary–tissue models, we prescribe a constant inlet capillary oxygen content at the capillary entrance and solve Cc(z,t) along the capillary (equation (24)). To account for changes in hemoglobin during trauma and resuscitation, we express hemoglobin content per blood volume as HCT×MCHC (g Hb/mL blood), yielding:

(24’) $$C_{c}(z,t)=\alpha_{t}\cdot P_{c}(z,t)+HCT\cdot \text{MCHC}\cdot O_{2Hb}\cdot SO_{2}\left(P_{c}(z,t)\right)$$

**2.** At each axial segment of the capillary, equation (24’) was re-written to solve for ${\text{SO}}_{2}\left({\text{P}}_{\text{c}}(\text{z},\text{t})\right)$ such that $SO_{2}\left(P_{c}(z,t)\right)=\frac{C_{c}(z,t)-\alpha_{t}\cdot P_{c}(z,t)}{HCT\cdot Hb_{RBC}\cdot \text{MCHC}}$. Then, the inverse of ${\text{SO}}_{2}\left({\text{P}}_{\text{c}}(z,\text{t})\right)$ was taken to calculate oxygen pressure along the capillary, ${\text{P}}_{\text{c}}(\text{z},\text{t})$, such that

(25) $$P_{c}(z,t)=f^{-1}\left(SO_{2}\left(P_{c}(z,t)\right)\right)=f^{-1}\left(\frac{C_{c}(z,t)-\alpha_{t}\cdot P_{c}(z,t)}{HCT\cdot Hb_{RBC}\cdot \text{MCHC}}\right)$$

To calculate the inverse of ${\text{SO}}_{2}\left({\text{P}}_{\text{c}}(\text{z},\text{t})\right),{\text{SO}}_{2}\left({\text{P}}_{\text{c}}(\text{z},\text{t})\right)$ was modeled according to the Adair equation^169^:

(26) $$SO_{2}\left(P_{c}(z,t)\right)=\frac{1}{4}*\frac{a_{1}PO_{2}+2a_{2}{\left(PO_{2}\right)}^{2}+3a_{3}{\left(PO_{2}\right)}^{3}+4a_{4}{\left(PO_{2}\right)}^{4}}{\left(1+a_{1}PO_{2}+a_{2}{\left(PO_{2}\right)}^{2}+a_{3}{\left(PO_{2}\right)}^{3}+a_{4}{\left(PO_{2}\right)}^{4}\right)}$$

Notably, ***the previous model approximated the Adair equation with a ninth order polynomial*** as:

(27) $$P_{c}(z)=7.0624\cdot C(z{)}^{9}+52.852\cdot C(z{)}^{8}+142.78\cdot C(z{)}^{7}+149.52\cdot C(z{)}^{6}+2.6376\cdot C(z{)}^{5}-81.241\cdot C(z{)}^{4}-7.5042\cdot C$$

Hence, eq. 22–24 were kept constant across an array of Hcts. ***In our model, this was adjusted such that a ninth order polynomial was approximated each iteration of the O2 module*** to account for changes in Hemoglobin and MCHC.

**3.** In previous models the calculation for ${\text{O}}_{2}$ concentration along a capillary segment was calculated by:

(28) $${Ca\;p}_{O2}(t+1)=$$

This was modified to account for the concentration of blood entering and exiting a capillary segment such that:

(29) $${Ca\;p}_{O2}\left(t+1\right)={Ca\;p}_{O2}\left(t\right)+2\cdot O2_{\text{conc.perUnitVol}}\cdot \left(Flow_{\text{In}}-Flow_{\text{Out}}\right)-\text{CapO}2_{\text{conc}.\text{Pres}}*O2_{\text{concGrad}}$$

Here, the ${\text{CapO}}_{2}$ is the concentration of ${\text{O}}_{2}$ in an axial segment of the capillary, ${\text{O}}_{2\;\text{concGrad}}$ is the oxygen concentration gradient, ${\text{Flow}}_{in}/Flow_{\text{out}}$ is the blood flow (of ${\text{O}}_{2}$) into or out of the capillary segment per unit volume (given by equation (30), ${\text{CapO}2}_{\text{concPress}}$ is the pressure exerted by the capillary ${\text{O}}_{2}$ (given by equation (31)) with the mass transfer coefficient (${\text{K}}_{\text{t}}$) defined by equation (32) (Eggleton et al. 2000).

(30) $$O2_{\text{concperUnitVol}}=Qb(\text{cycle})*\frac{\text{secperbeatpercycle}(\text{cycle})}{{\text{capillarysegmen}t}_{\text{volume}}}$$

(31) $$\text{CapO}2_{\text{conc.}\text{Pres}}=\frac{2}{r_{\text{capillary}}}\cdot dt\cdot K_{t}$$

(32) $$k_{t}=1.21-4.3\cdot Hct+\left(23.6\cdot Hct^{2}\right)\cdot 10^{-6}$$

In the previous model, Qb, the capillary blood flow was scaled to obtain a normal capillary blood flow and set to 7.7*10^−9^ ml/sec. However, upon review and for clarity, in the first iteration of the ${\text{O}}_{2}$ module, Qb (ml/s) is initially defined as (7.7*10^−9^ / 1.2e9), and then subsequent iterations Qb is equal to eq. 12

(33) $$Qb(t)=\frac{\sum \left(Q_{\text{branch}\backslash \#2}(1:N)\right)}{N}=\frac{1}{N^{*}s}\prod_{1}^{N}Q_{\text{branch}\backslash \#2}$$

In our model, Qb is defined as 7.7*10^−9^ m/s during the first step of equilibration (up to t=1 minute), and then equation (33) is used throughout the simulation with a scaling factor (s) of 1.2e9. Additionally, **the previous model assumed *a constant mass transfer coefficient across a range of Hct*. *We have implemented*** equation (32) ***such that it is calculated each time the*** $O_{2}$ ***module is initiated***. However, a limitation in this study is that this quadratic equation is derived from a fit curve which only models hematocrits in a range of 0.25 to 0.55 and hence equation (32) may not capture mass transfer at very low or high hematocrits.

#### Alveolar Module

**4.** In order to calculate the subsequent cycles starting capillary ${\text{O}}_{2}$ concentration (${\text{C}}_{\text{c}}(1,1)$ equation (24’), an “alveolar module” was implemented to model the pulmonary capillary and alveolus. Thus, a cyclic model was implemented in which blood transports oxygen to the tissue capillary (Figure 1c). There oxygen diffuses into the tissue during tissue capillary flow as blood moves through the capillary at speed, Qb, until the capillary blood flows out of the venous end of the capillary into the systemic circulation. Then this venous blood passes to the arterial end of the alveolar module and gets oxygenated from the alveolus as the blood flows through the alveolar capillary. Blood then continues from the venous end of the alveolar module to the arterial end of the tissue capillary. This module was functionally equivalent to the tissue capillary module with a few key differences. ***A*.** The alveolus was assumed to be at a fixed partial pressure of oxygen (Table S10). This allowed oxygen to flow from the alveolus into the pulmonary circulation. ***B***. To our knowledge there are no reported equations describing the pulmonary capillary mass transfer coefficient (${\text{k}}_{\text{t}}$) and hence equation (29) was used for the mass transfer coefficient of the alveolar module. ***C*.** Diffusion coefficient parameters were adjusted to reflect diffusion through air (vs. tissue) according to reported values (Table S10). For increased speed in this module, there were assumed to be 3 tissue segments between the pulmonary capillary and the alveolus.

The dynamic time dependent equations were solved numerically using the finite difference method with the boundary conditions. The initial conditions for the capillary are defined in (Table S11, Table S12) with the initial arterial ${\text{PO}}_{2}=100\;\text{mmHg}$ such that ${\text{C}}_{\text{c}}\left(1,1\right)=0.1880\;{\text{mIO}}_{2}/\text{mL}$, and venous ${\text{PO}}_{2}=40\;\text{mmHg}$ and alveolar atmospheric ${\text{O}}_{2}=104\;\text{mmHg}$.

At each iteration of the ${\text{O}}_{2}$ module, steady state results were obtained over N iterations in 1 cycle from the solution of the time dependent equations. The finite difference method was solved by modeling the capillary as a series of resistors defined in Figure 1B and Table S10. The ${\text{O}}_{2}$ module was computationally intensive, and the module was run iteratively every one minute of simulated time, but could be modified depending on the user’s needs. The ${\text{PO}}_{2}$, oxygen consumption, and capillary pressure were recorded at each iteration of this module.

#### Lactate Module

Given the simulation of tissue oxygenation ${\text{PO}}_{2}$, oxygen consumption, capillary oxygen pressure and the cardiovascular module, oxygen delivery (${\text{DO}}_{2}$) could be calculated at each iteration of the oxygenation module via:

(34) $${DO}_{2}=CO\cdot {CaO}_{2}=CO\cdot \left[1.34\cdot Hb\cdot {SaO}_{2}+\left(0.003\cdot {PaO}_{2}\right)\right]$$

(35) $$CO=HR*SV=HR*\left(max\left(V_{LV}\right)-min\left(V_{LV}\right)\right)$$

where CO is cardiac output, CaO2 is arterial oxygen content, 1.34 ml/g is the oxygen binding capacity of hemoglobin, Hb is hemoglobin in g/dL, SaO2 is the percent hemoglobin oxygen saturation, 0.003 represents the amount of dissolved oxygen in 100 ml of blood, and ${\text{PaO}}_{2}$ is the arterial partial pressure of oxygen^170^. Cardiac Output (equation 35) now accounts for variations in heart rate (HR) and stroke volume (SV) incorporates the changes to the heart cycle described above.

Using equation (34) and oxygen consumption, the lactate at specific values of ${\text{DO}}_{2}$ could be interpolated^171^. A critical DO_2_ threshold of ~165 mL/min was set based on this data. When a patient’s ${\text{DO}}_{2}$ was less than this critical ${\text{DO}}_{2}$, they were considered to be in an “oxygen deficient” state followed by an accumulation of oxygen debt. Over the course of the simulation, if a patien’s DO2 was restored above the critical ${\text{DO}}_{2}$ threshold the “oxygen debt” stopped accumulating and began to reverse as oxygen deficit returns to ≤ 0.

#### Stochastic model

Our model accounted for variations in trauma physiology by randomizing a series of patient characteristics during the initialization stage of the simulation (Figure S7) with the randomized parameters outlined in Table S4, S5, and S6. For example, the patient’s baseline weight^172^, age^173^, and height^172^ were stochastically defined. Height and weight were set as covariates according to the Pearson correlation coefficient outlined in (Table S4; Figure S7E)^174^. A patient’s sex was randomized based on distributions of patients who received RBC or LTOWB within four hours of arrival to the trauma center from the Trauma Quality Improvement Program (TQIP) database^75^ as opposed to the percentage of all fatal and nonfatal injuries in the U.S. in 2022^175^. For simplicity a correction factor was used, as opposed to using arm circumference and subcutaneous fat to predict a patient’s SBP^176^.

The “resting” heart rate was randomized according to the average HR of patients who presented to the ED^177^, a patient was randomly assigned to a “shock class” based on the distribution of patients who in a specific ATLS shock class^10,76^. The reported blood volume loss is <15%, 15–30%, 30–40%, and >40% for ATLS class I, II, III, and IV^10^. As described above, using the calculated blood volume and selected ATLS class, a bleeding rate is calculated such that at a constant bleeding rate, they would lose a volume of blood equal to a randomly selected percent of blood volume within their respective ATLS class.

Given a specific ATLS class, a subset of patients were then randomized to have a “hidden bleed” which would simulate injury which could not be directly tamponaded and which would lead to a mortality expected for a given ATLS class (ex. ~35% of ATLS Class IV patients had a “hidden bleed” which continued to bleed even at low SBPs).

Hematocrit, Fibrinogen level, INR, and Platelet count were all stochastically chosen assuming a normal distribution derived from data from a level I trauma center (unpublished data from UPMC Presbyterian Hospital). Variations in heart chamber sizes are described above in the Cardiovascular module section. Stochastic variations in blood products are described in the resuscitation, goal directed therapy, and user defined resuscitation module section of the methods.

To ensure that comparisons between different resuscitation strategies were as controlled as possible, simulations for each resuscitation strategy (WB, CCT, etc.) were run using a single stochastic patient’s characteristics (constant height, weight, starting hematocrit, etc.) and then compared across all patients. All stochastic simulations were run in parallel using MATLAB on a high-performance computing cluster with the SLURM workload manager.

## Supplementary Material

1

## Data Availability

The stochastic multi compartment model of trauma resuscitation and the additional supporting modules are accessible upon request.
