# Supplementary material for: A stochastic, physiology-based digital twin model of hemostasis and oxygenation in trauma resuscitation
Source: Res Sq. 2026 Jul 8:rs.3.rs-10273801. Preprint. [Version 1] doi: 10.21203/rs.3.rs-10273801/v1 (PMC13370647; doi:10.21203/rs.3.rs-10273801/v1)
Supplement: 1 [file NIHPPRS10273801V1-supplement-1.pdf]

Supplemental Figures

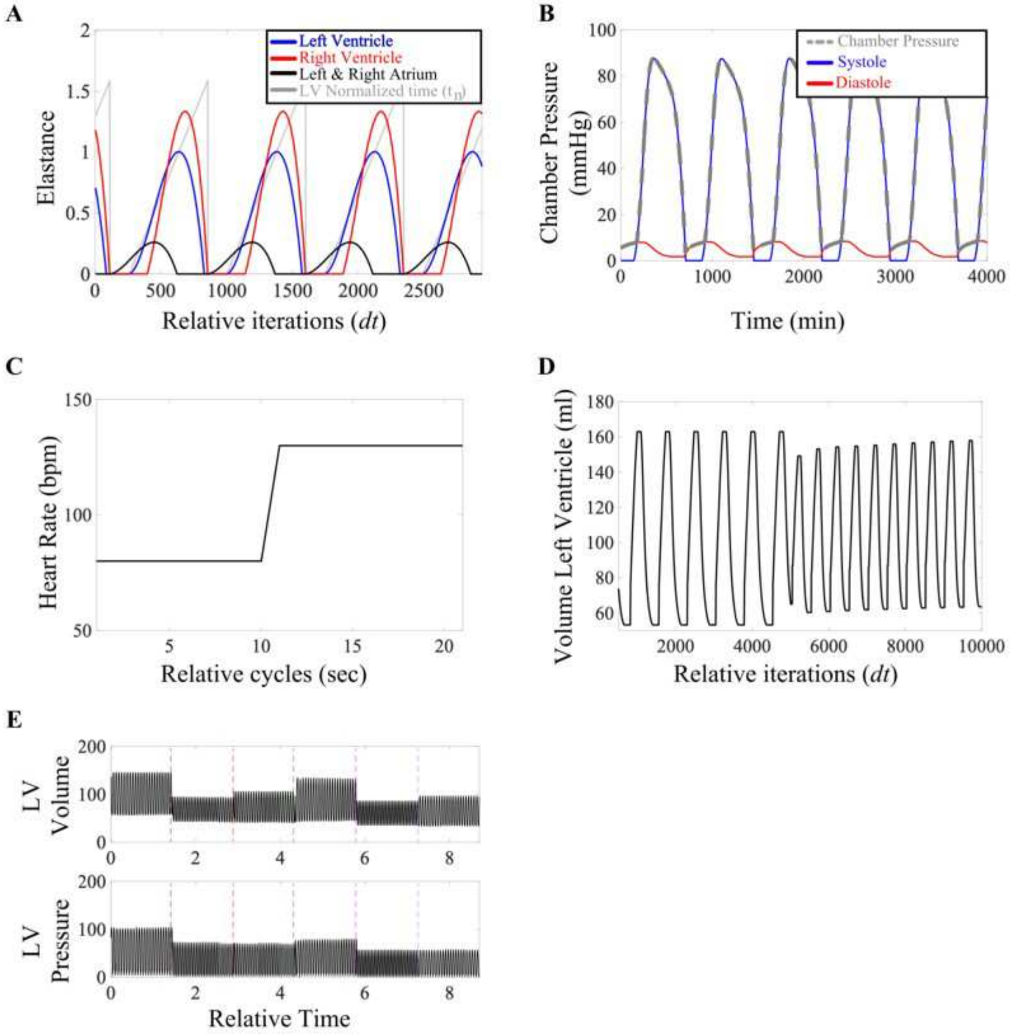

Figure S1:

Example output of various parameters of the cardiovascular module in a single deterministic patient in the comprehensive model. (A) The time normalized elastance (E_n) over the normalized time period (t_n; gray) for each heart chamber with a peak elastance (E_{max}) of 1 for the left ventricle (blue) at the moment of systole. The left and right atrium (black) reach a peak elastance earlier than the left and right ventricle to account for the contraction of the sinoatrial (SA) node before the atrioventricular (AV) node. (B) Left ventricle systole (blue) and diastole (red) pressure curves over the course of 5 heart cycles in the equilibration phase. Chamber pressure (gray dashed line) at time *t* is given as the maximum of the comparison of systolic and diastolic pressure time point *t*. (C) A time varying heart rate (HR) over several cycles of the simulation shows (D) volume contraction over the constant time step, *dt*, due to decreased chamber filling time. (E) Volume contraction and relative pressure stability observed in the left ventricle volume/pressure traces over 60 seconds of relative time at the end of each simulation phase (corresponding to Figure 2C).

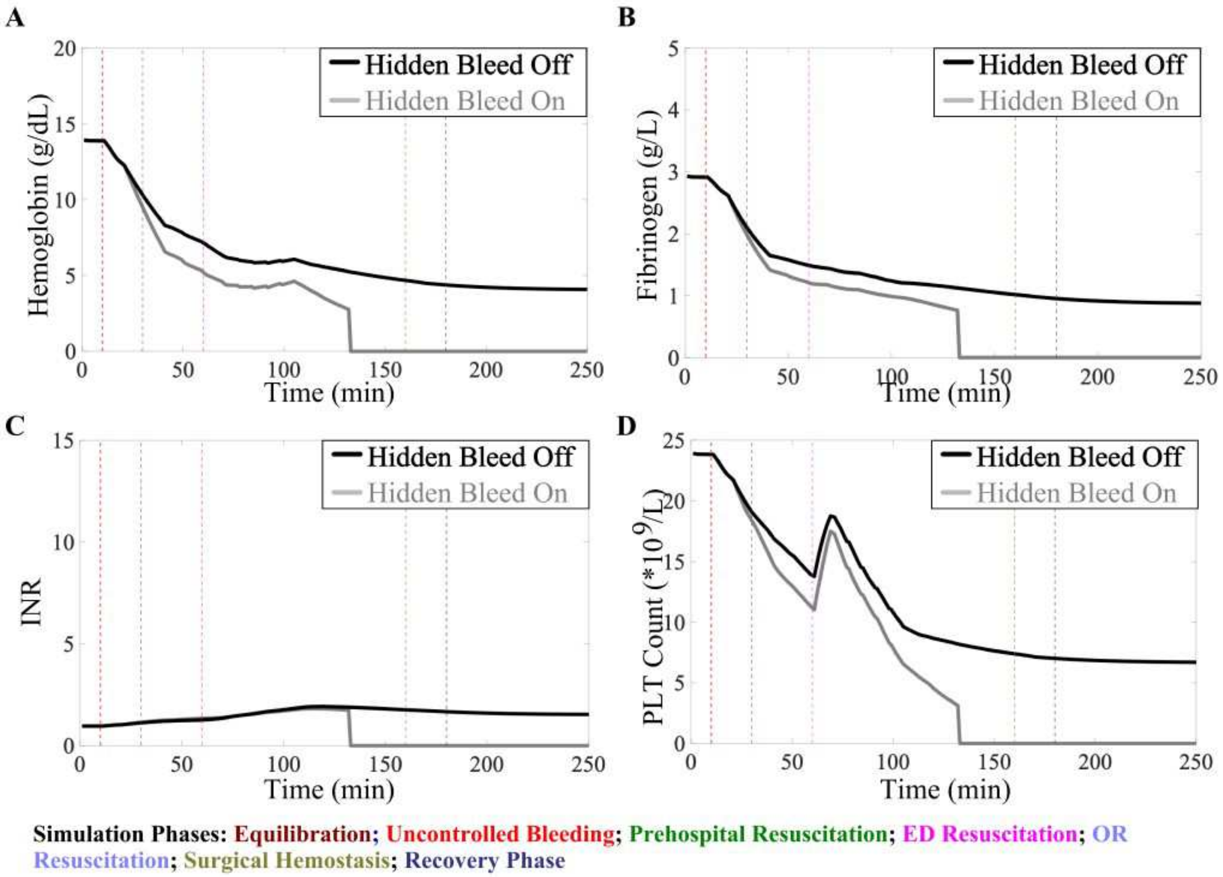

Figure S2:

Illustrative example of a simulation of a single deterministic patient in the comprehensive model comparing a patient with and without the hidden bleed parameter. Changes in (A) hemoglobin, (B) fibrinogen, (C) INR, and (D) platelet (PLT) count. In each simulation 1L of crystalloid fluid was administered in the pre-hospital resuscitation phase and then followed by 2 units of low titer group O whole blood (WB) in the emergency department (ED) and 6 units of conventional component therapy (CCT) at a ratio of 6 units of red blood cells, 6 units of plasma, and 1 unit of platelets.

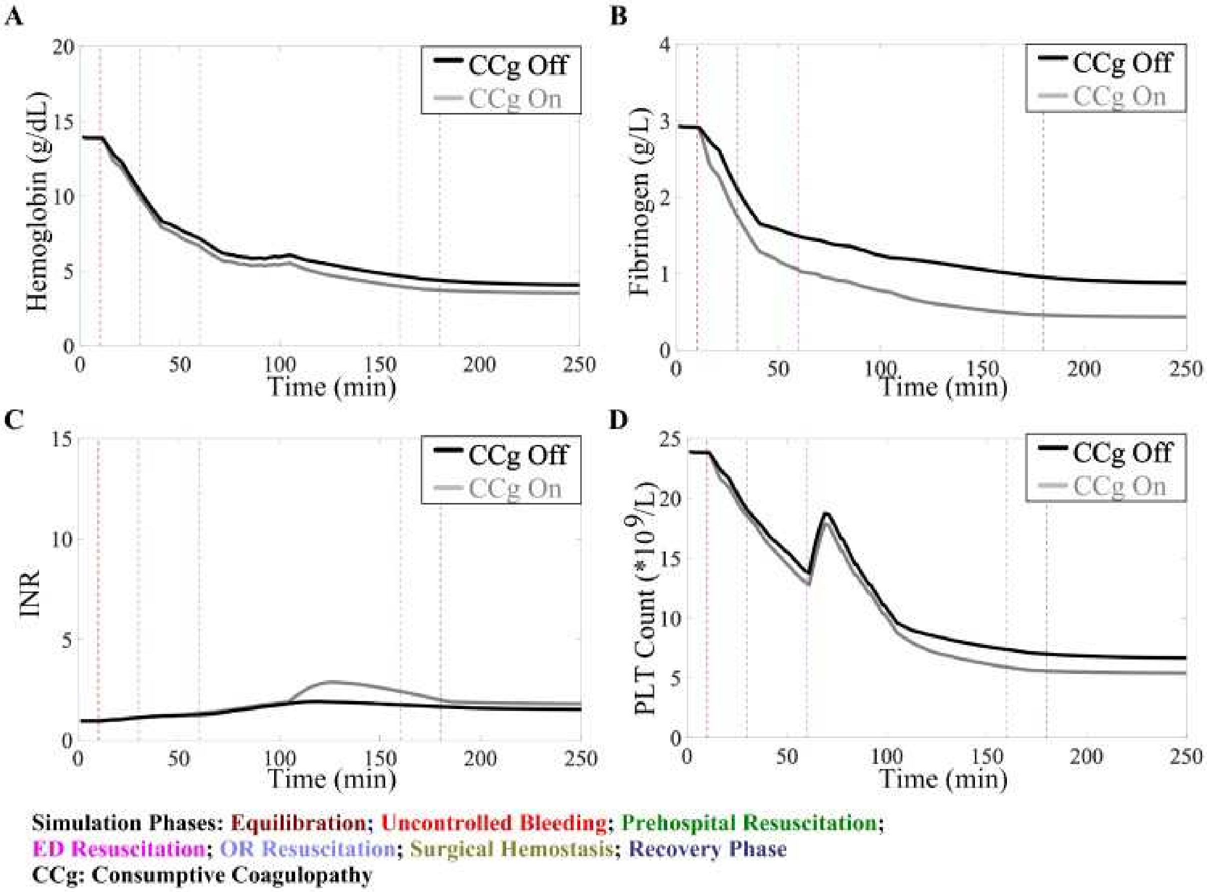

Figure S3:

Illustrative example of a simulation of a single deterministic patient in the comprehensive model comparing a patient with and without consumptive coagulopathy (CCg). Changes in (A) hemoglobin, (B) fibrinogen, (C) INR, and (D) platelet (PLT) count. In each simulation 1L of crystalloid fluid was administered in the pre-hospital resuscitation phase and then followed by 2 units of low titer group O whole blood (LTOWB) in the ED and 6 units of conventional component therapy (CCT) at a ratio of 6 units of red blood cells, 6 units of plasma, and 1 unit of platelets.

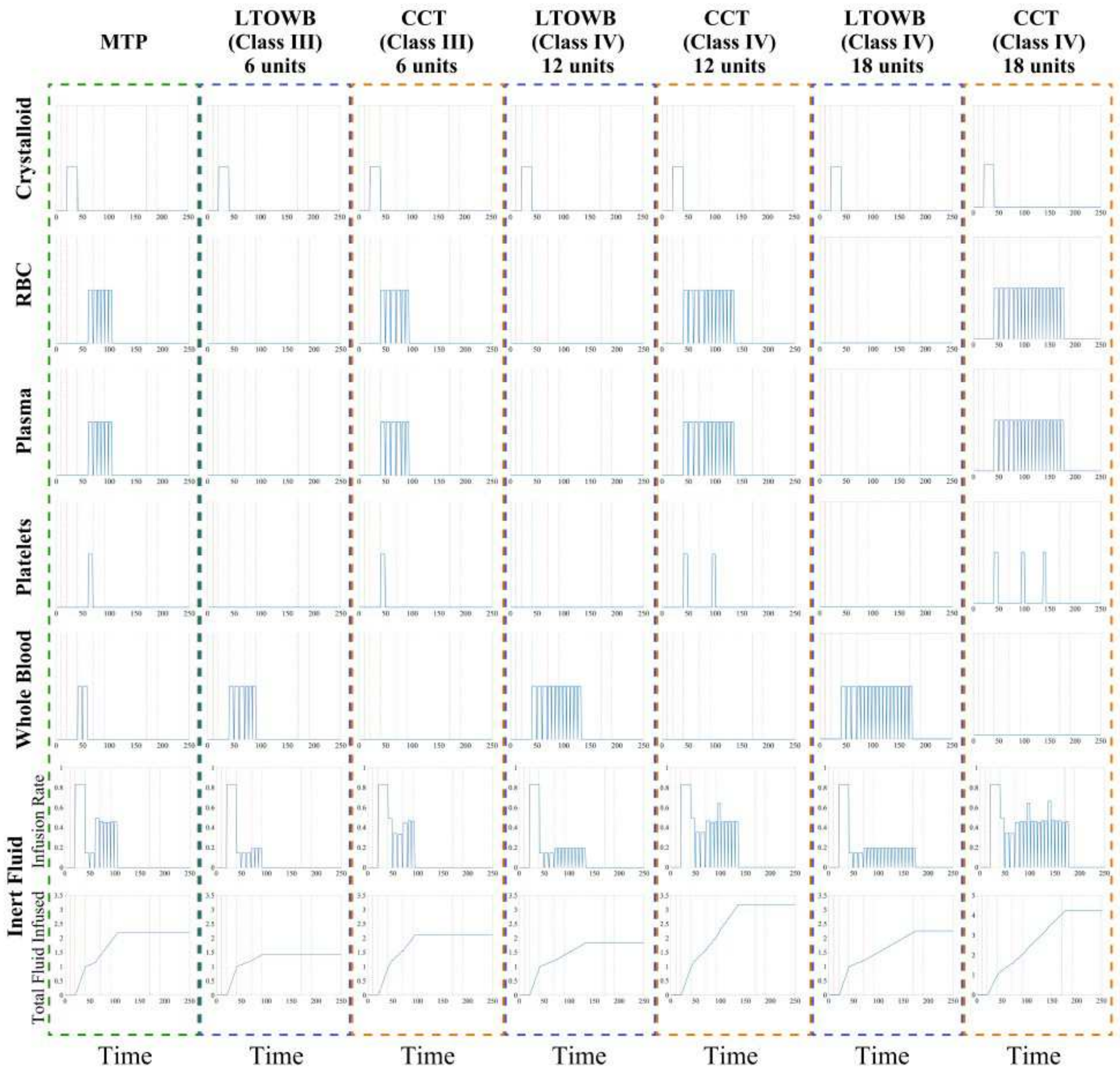

Figure S4:

Graphical representation of the resuscitation strategies used in the simulations described in this study. Resuscitation is defined according to a pulse table with either a “0” (no infusion) or “1” (infusion given) throughout each cycle of the simulation. Crystalloid, RBCs, plasma, platelets, low titer group O whole blood (LTOWB), and the inert fluid infusion rate and total fluid transfused are shown for the following resuscitation strategies: 2 WB units and 1 MTP (used in Figure 2, Figure 3, and Figure 4), 6 units of LTOWB or CCT for the class III (>30% and ≤40% blood volume lost) patients of the simulated trial, 12 units of LTOWB CCT for the class IV (>40% blood volume lost) patients in the of the simulated trial, and 18 units of LTOWB or CCT (used in Figure S5).

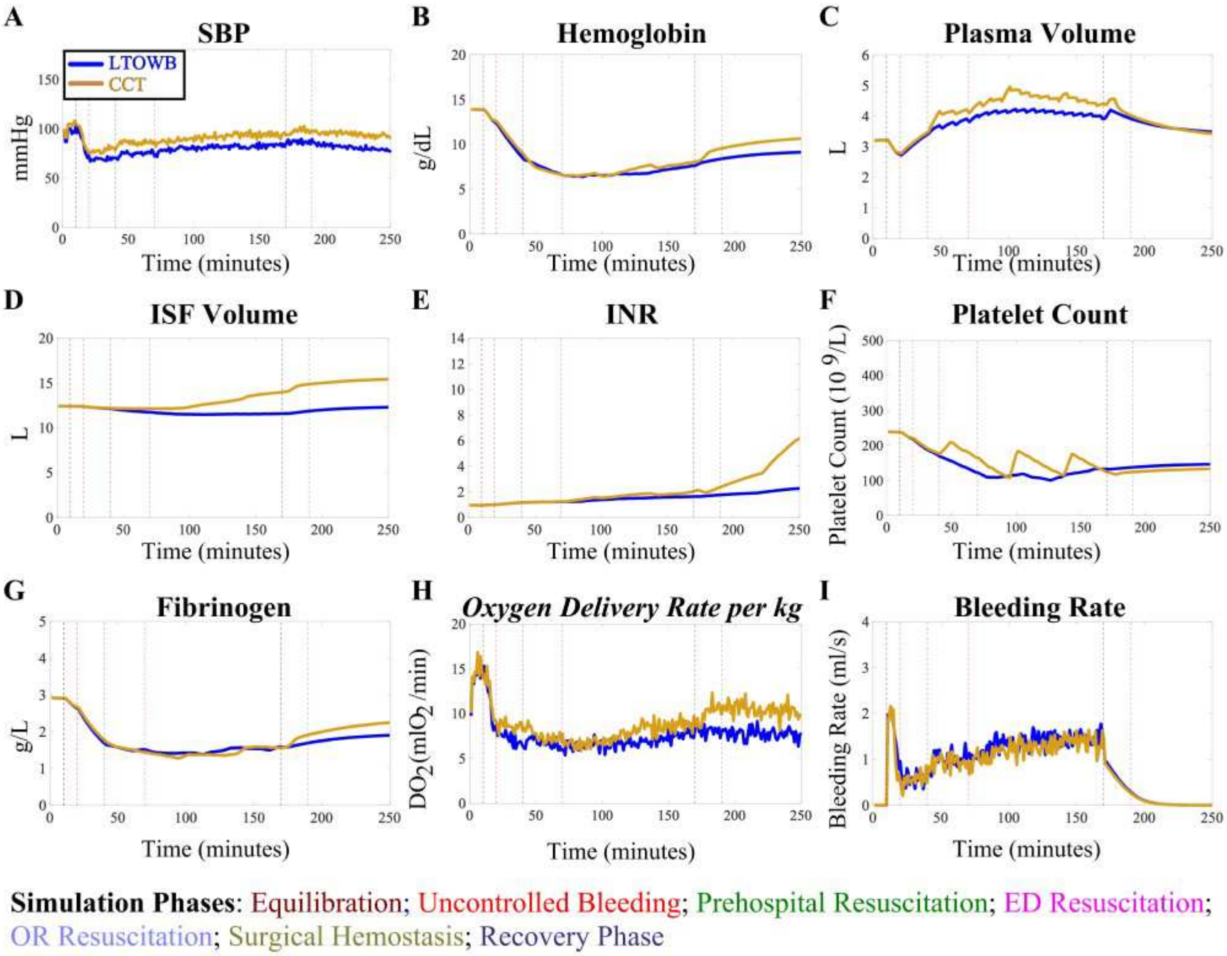

Figure S5:

Illustrative examples of a comparison between low titer group O whole blood (LTOWB; blue) and conventional component therapy (CCT; orange) for a single, ATLS class IV (>40% blood volume lost), deterministic patient receiving 18 units of the respective therapy. (A) SBP, (B) Hemoglobin, (C) Plasma volume, (D) ISF volume, (E) INR, (F) Adjusted platelet count, (G) Fibrinogen, (H) Oxygen Delivery Rate per kg, and (I) Bleeding rate.

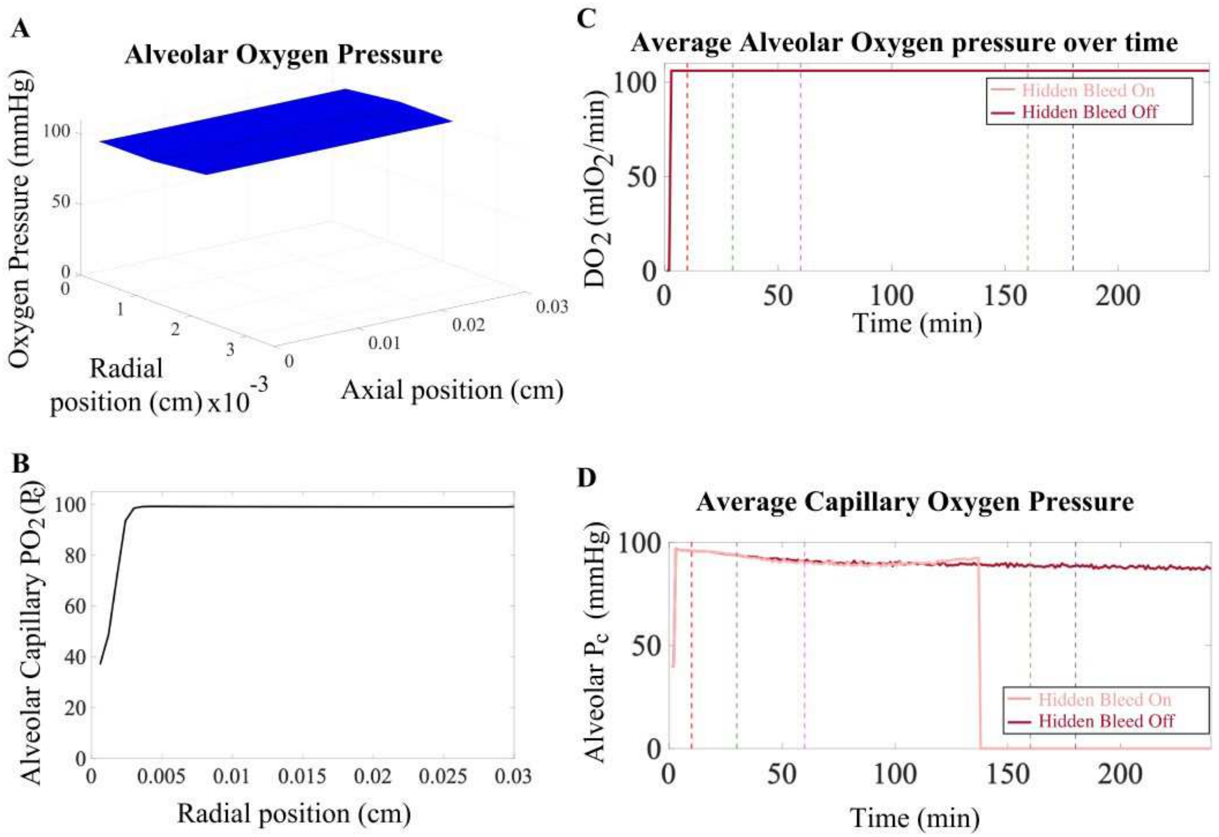

Figure S6:

Example output of various parameters of the alveolus module in a single deterministic patient. (A) Alveolar oxygen diffuses towards the lung capillary and (B) alveolar oxygen pressure in the outermost shell is at a constant pressure at approximately atmospheric pressure (~104 mmHg). (C) Lung capillary PO_2 increases from ~40 mmHg to ~104 mmHg along the radial position. (D) Average oxygen pressure across the alveolar capillary segments (N=50) decreases with decreases in hemoglobin over the length of the simulation. Where indicated the pale pink and the dark red curves correspond to a single determinist patient with the “hidden bleed” parameter turned on or off, respectively.

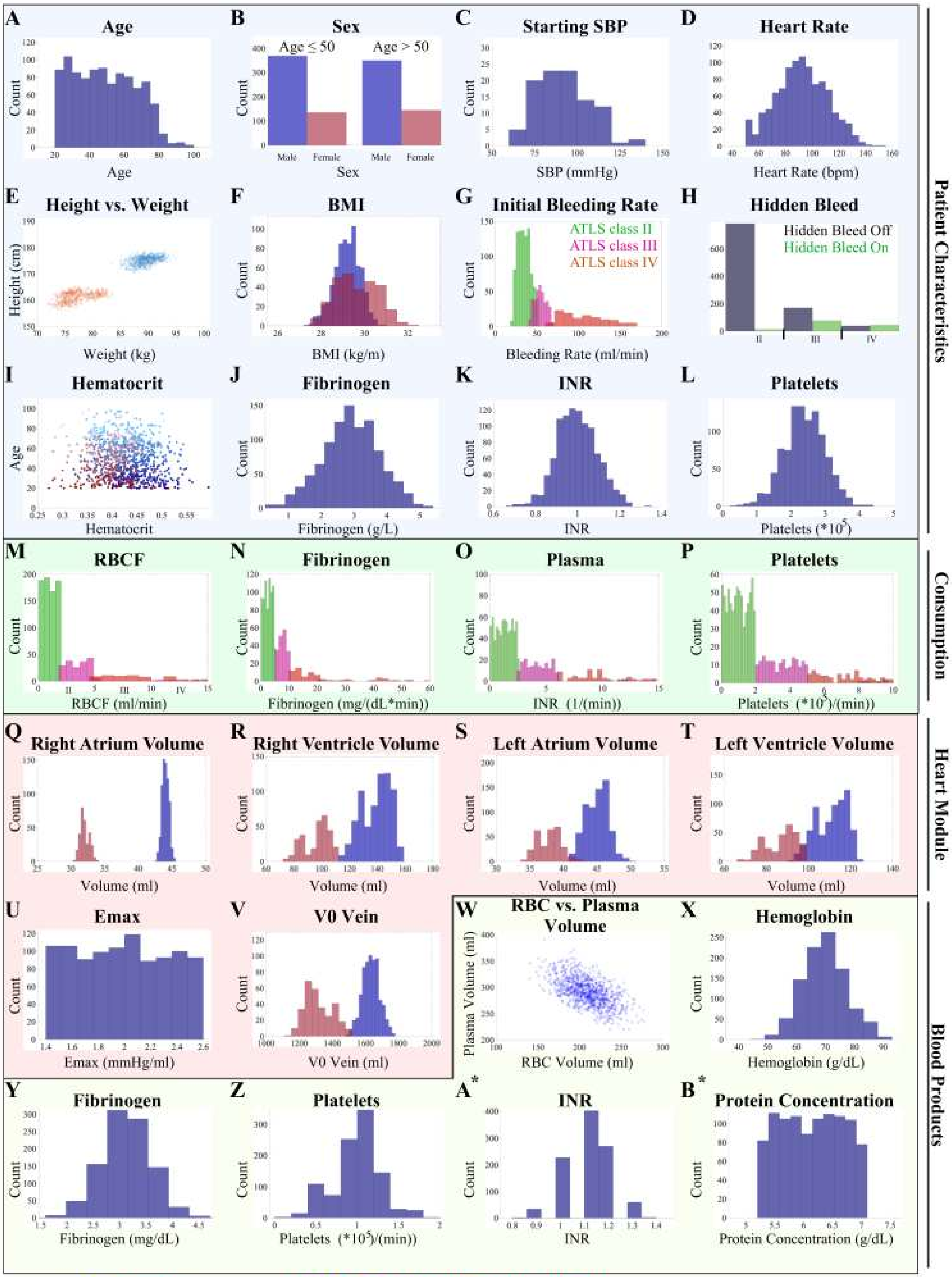

1293 N=1000; **Male; Female;** ATLS Class: **II (15-30%), III (30-40%), IV (>40%) ;** Hidden Bleed & Trauma Induced Coagulopathy: **ON**, **OFF**

Figure S7:

Distribution of values for each stochastically determined variable prior to beginning the simulation. A patient's (A) age, (B) sex, (C) SBP at the end of equilibration, (D) heart rate, (E) height and weight, (F) body mass index (BMI), (G) initial bleeding rate, (H) "hidden bleed" parameter per ATLS class, (I) Hematocrit as a function of age and sex, (J) fibrinogen, (K) INR, (L) platelet count, (M) trauma induced coagulopathy (TIC) parameter per ATLS class, (N) fibrinogen consumption factor, (O) plasma consumption factor, (P) platelet consumption factor, (Q) right atrium volume, (R) right ventricle volume, (S) left atrium volume, (T) left ventricle volume, (U) E_{max}, and (V) "unfilled" venous volume (V_0) vein. Additionally, each unit of a blood product was simulated as derived from a whole blood donation with a series of randomized parameters including the correlation of (W) RBC and plasma volume, (X) hemoglobin, (Y) fibrinogen, (Z) platelets, (A*) INR, and (B*) protein concentration.

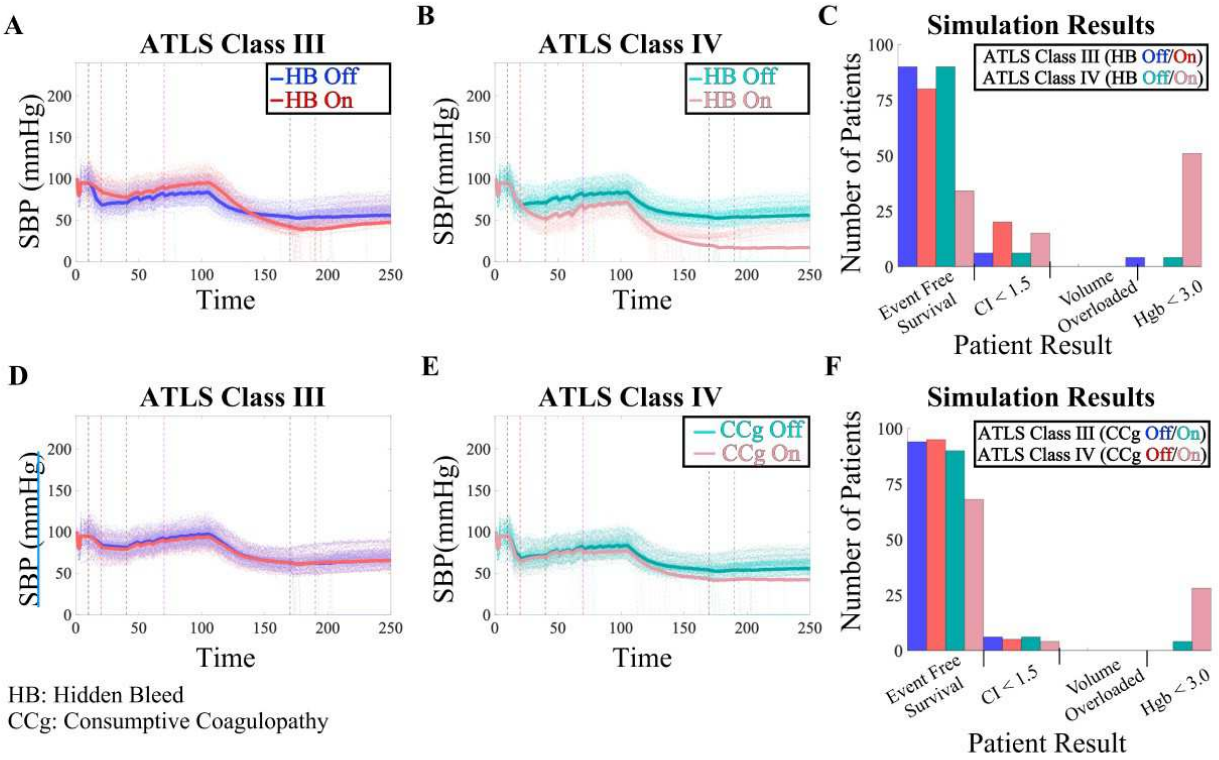

Figure S8:

Illustrative examples of the effect of the hidden bleed and consumptive coagulopathy (CCg) parameters on 100 stochastic ATLS class III (>30% and ≤40% blood volume lost) and 100 stochastic class IV (>40% blood volume lost) patients in the comprehensive model. (A) ATLS class III and (B) ATLS class IV patients with a "hidden bleed" experience lower systolic blood pressures (SBP). (C) The "hidden bleed" parameter was scaled such that the distribution of results for the simulation approximately matches the reported mortality for ATLS class III and class IV patients. (D) ATLS class III and (E) ATLS class IV patients with CCg (and the hidden bleed parameter turned *off*) experience similar SBPs over time. A greater number of class IV patients expire leading to a decreased mean SBP for class IV patients. (F) The CCg parameter was scaled to approximately match reported mortality for ATLS class IV patients. In each simulation 1 L of crystalloid fluid was administered in the pre-hospital resuscitation phase and then followed by two units of low titer group O whole blood (LTOWB) in the emergency department (ED), followed by 1 massive transfusion protocol (MTP) packet consisting of six units of conventional component therapy (CCT) at a ratio of 6 units of red blood cells, 6 units of plasma, and 1 unit of platelets.

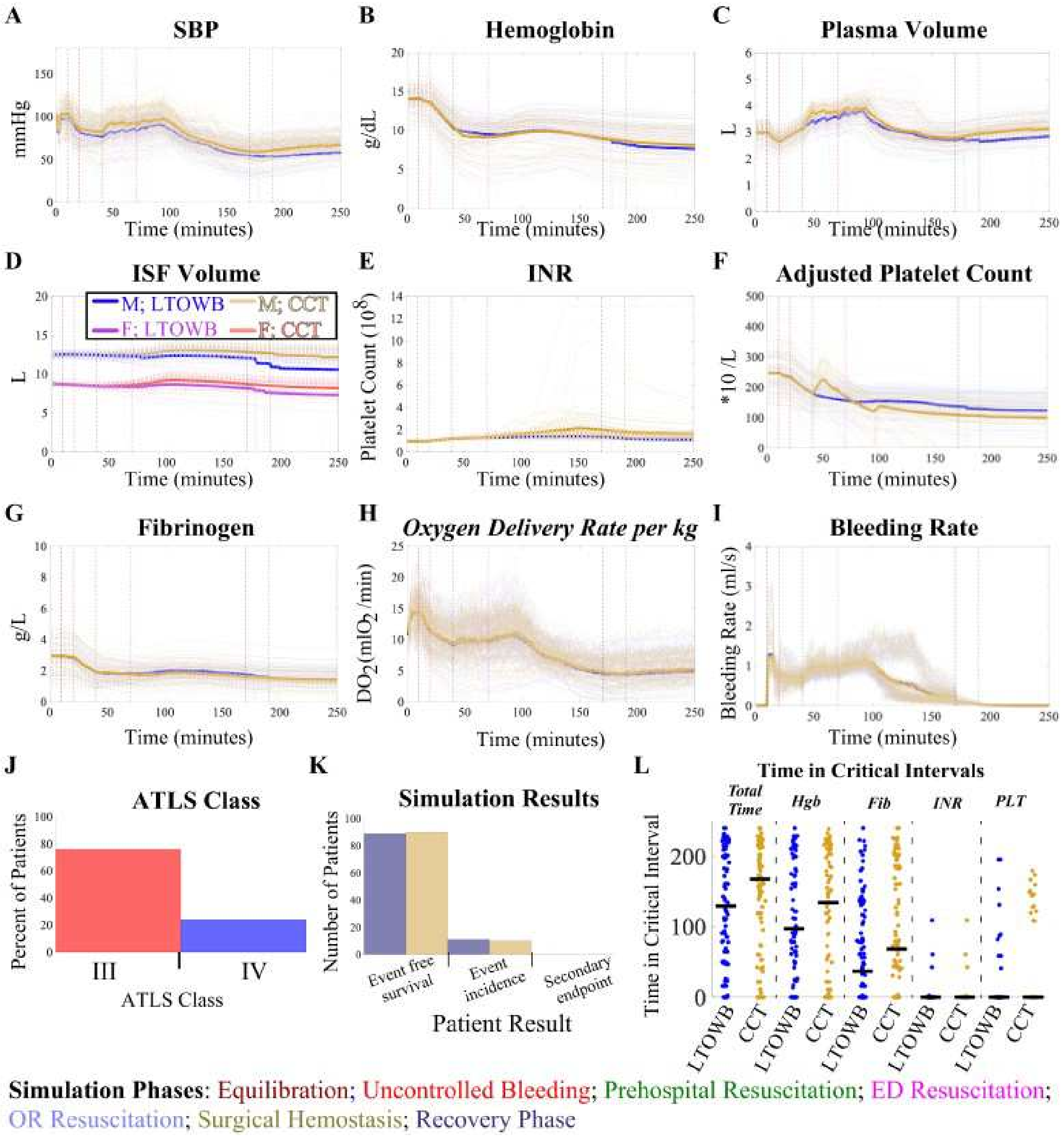

Figure S9:

Comparison of the simulations of 100 stochastically generated patients (including with a randomized ATLS class) comparing 6 units of low titer group O whole blood (LTOWB; blue line) versus 6 units of conventional component therapy (CCT; orange). The changes in (A) SBP, (B) Hemoglobin, (C) plasma volume, (D) Interstitial fluid volume (ISF), (E) international normalized ratio (INR), (F) platelet (PLT) count, (G) fibrinogen, (H) oxygen delivery rate per kg, and (I) the bleeding rate over time are shown. The value for a single patient is shown as a semi-transparent, dashed blue line (LTOWB) or orange line (CCT), with the mean value for all patients shown as a solid, opaque blue (LTOWB) or orange (CCT) line. (J) 75% and 25% of the patients randomized to ATLS class III (>30% and ≤40% blood volume lost) or IV (>40% blood volume lost) respectively. (K) 95% (LTOWB) and 92% (CCT) of patients resulted in event free survival. (L) Time in critical intervals for Hemoglobin < 8 g/dL, INR ≥1.5, fibrinogen level <150 mg/dL, or PLT count <50 × 10^9/L.

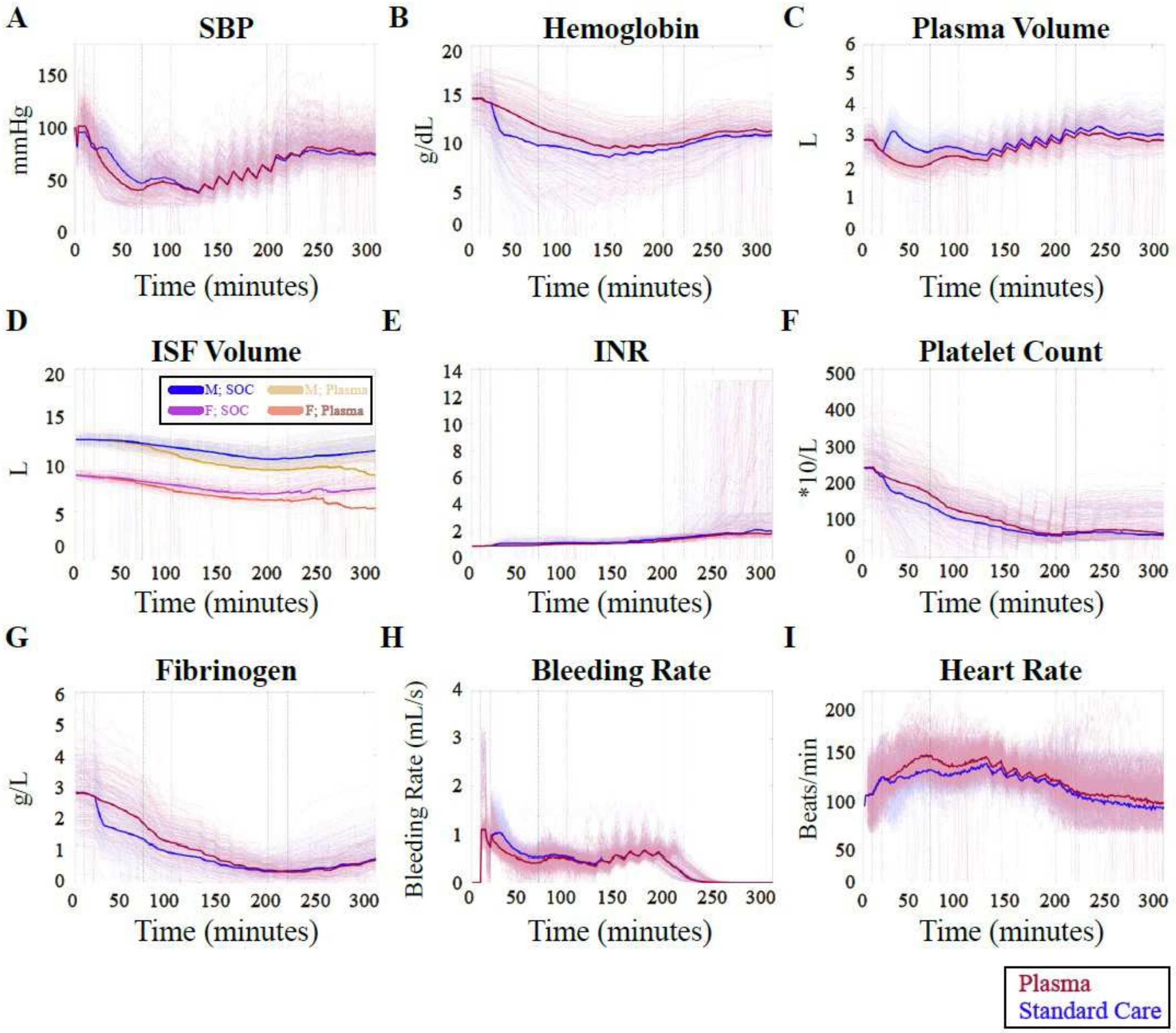

Figure S10:

Comparison of the simulations of 250 stochastically generated patients for modelling the Prehospital Air Medical Plasma (PAMPer) trial. The changes in (A) SBP, (B) Hemoglobin, (C) plasma volume, (D) Interstitial fluid volume (ISF), (E) international normalized ratio (INR), (F) platelet (PLT) count, (G) fibrinogen, (H) the bleeding rate, and (I) heart rate over time are shown. The value for a single patient is shown as a semi-transparent, dashed blue line (standard of care [SOC]; 1L of crystalloid) or red line (intervention arm; plasma [PL]), with the mean value for all patients shown as a solid, opaque blue (standard of care) or red (intervention arm) line. All simulated patients were simulated in both the SOC and PL arm, and the prehospital times were stochastically selected based on criteria reported in the PAMPer trial. The vertical lines correspond to approximate time points for the end of equilibration (maroon; t = 10 minutes), the time at which emergency medical services (EMS) arrives (red; t = 20 minutes), the end of prehospital resuscitation and start of the emergency department (ED) phase (green; t = 60), the end of the ED and start of the operating room (OR) phase (pink; t = 91 minutes), time

of surgical hemostasis (black; t = 191 minutes), and the start of the recovery phase (blue; t = 211 minutes). The recovery phase was truncated for all patients at minute 300.

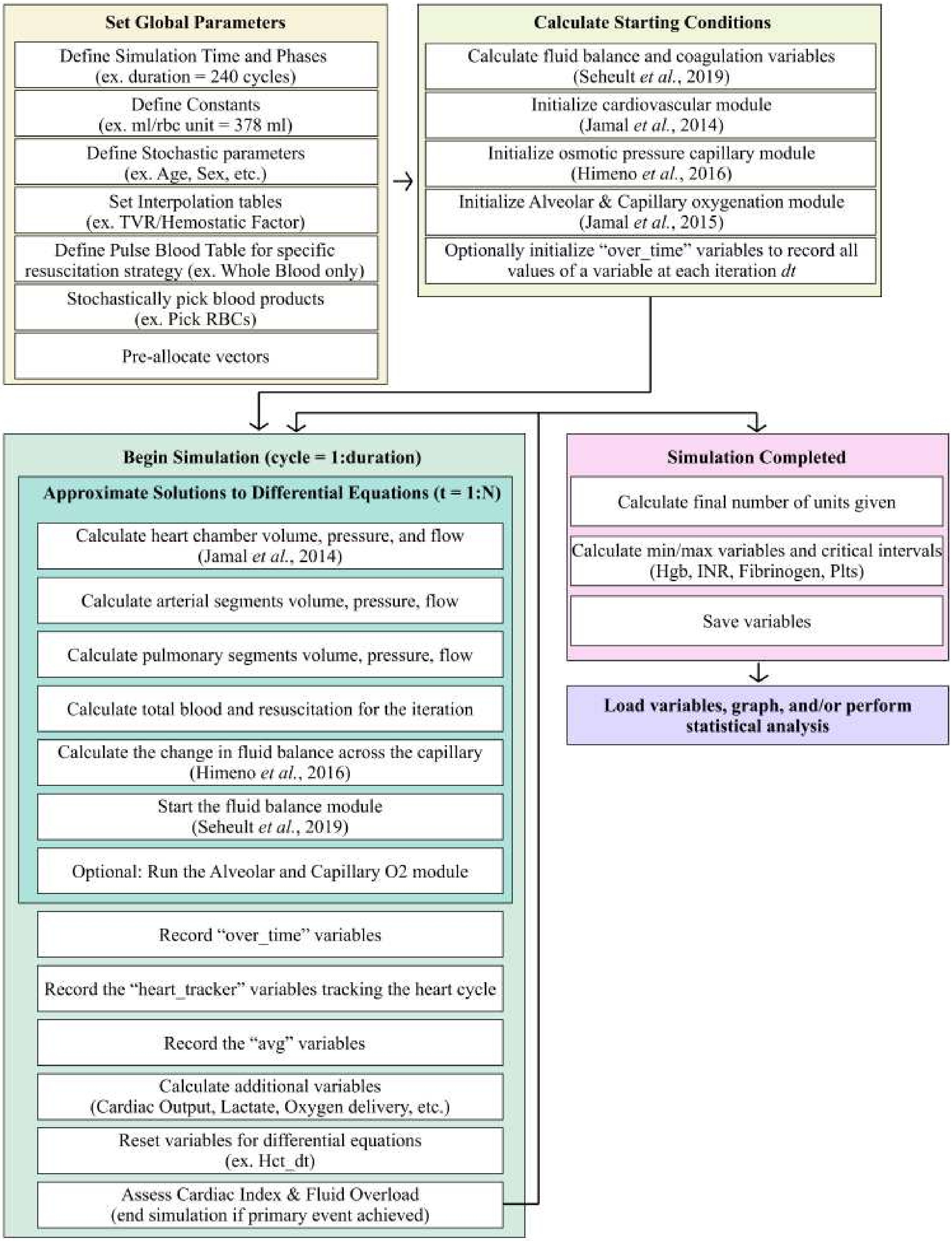
Set Global Parameters
Define Simulation Time and Phases
(ex. duration = 240 cycles)
Define Constants
(ex. ml/rbc unit = 378 ml)
Define Stochastic parameters
(ex. Age, Sex, etc.)
Set Interpolation tables
(ex. TVR/Hemostatic Factor)
Define Pulse Blood Table for specific resuscitation strategy (ex. Whole Blood only)
Stochastically pick blood products
(ex. Pick RBCs)
Pre-allocate vectors
Calculate Starting Conditions
Calculate fluid balance and coagulation variables
(Scheult et al., 2019)
Initialize cardiovascular module
(Jamal et al., 2014)
Initialize osmotic pressure capillary module
(Himeno et al., 2016)
Initialize Alveolar & Capillary oxygenation module
(Jamal et al., 2015)
Optionally initialize "over_time" variables to record all values of a variable at each iteration dt
Begin Simulation (cycle = 1:duration)
Approximate Solutions to Differential Equations (t = 1:N)
Calculate heart chamber volume, pressure, and flow
(Jamal et al., 2014)
Calculate arterial segments volume, pressure, flow
Calculate pulmonary segments volume, pressure, flow
Calculate total blood and resuscitation for the iteration
Calculate the change in fluid balance across the capillary
(Himeno et al., 2016)
Start the fluid balance module
(Scheult et al., 2019)
Optional: Run the Alveolar and Capillary O2 module
Record "over_time" variables
Record the "heart_tracker" variables tracking the heart cycle
Record the "avg" variables
Calculate additional variables
(Cardiac Output, Lactate, Oxygen delivery, etc.)
Reset variables for differential equations
(ex. Hct_dt)
Assess Cardiac Index & Fluid Overload
(end simulation if primary event achieved)
Simulation Completed
Calculate final number of units given
Calculate min/max variables and critical intervals
(Hgb, INR, Fibrinogen, Plts)
Save variables
Load variables, graph, and/or perform statistical analysis

Figure S11:

Overall schematic representation of the code for the multicompartment model of hemostasis and oxygenation. First, global parameters defining constants, stochastic parameters, and resuscitation parameters are defined. Then, vectors are pre-allocated and starting conditions are calculated from initial parameters. The simulation begins with loop through a specific “cycle” equal to 1 second of simulation time with differential equations approximated in a second loop through time steps *dt* from 1:N where equals 500. Each cycle the patient is assessed for the primary endpoint, and upon meeting the primary endpoint or reaching the duration of the simulation (cycle = 15,000) the simulation is completed. Variables are then saved and can be loaded into secondary scripts for visualization or statistical analysis.

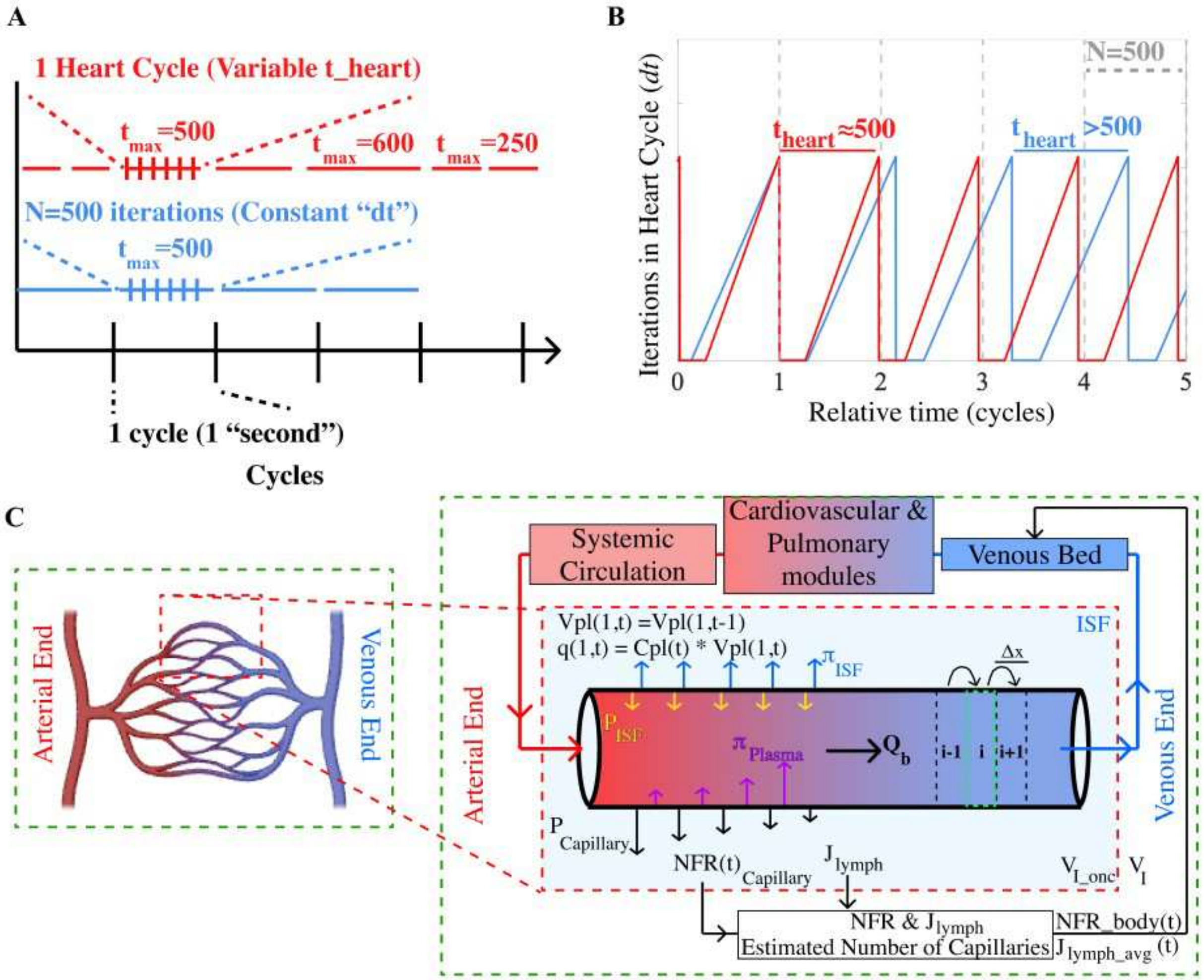

Figure S12:

Schematic representation of the modifications to the cardiac module and oncotic pressure/net filtration module implemented in the multicompartment model. (A) The cardiac cycle as described in (Siam, Mandel, and Barnea 2014, 2) was modified (see methods) such that 1 cycle, which corresponds to 1 "second" of simulation time, is then broken down into a constant, defined number of N time steps (*dt*; blue curve*)* ***and*** a variable number of steps tracking the relative time point in the heart cycle (t_{heart}; red curve). (B) Example snapshots from a simulation show the relative time of the heart cycle (t_{heart}) at a heart rate (HR) of ~60 bpm (red) where t_{heart} is ≈ N. When HR is >>60 (blue), t_{heart} is > 500 and becomes out of sync with the "cycle time" over the course of the simulation. Both curves were manually aligned to peak at a relative time of 1 for this illustration. (C) The oncotic pressure module was modified such that the arterial system feeds into a capillary network which is continuous with the venous system. Filtration at the capillary, $NFR(t)_{Capillary}$, occurs via the Starling equation through hydrostatic (P_{cap} and P_{ISF}) and oncotic pressure (π_{Plasma} and π_{ISF}) and leads to lymphatic flow at the capillary, J_{lymph} per Himeno *et al*. (2016)[68]. In contrast to Himeno *et al*., in which only a single capillary ($NFR_{Capillary}$ and J_{lymph}) was modeled, a global filtration value was estimated from the total number of capillaries to calculate the total filtration of the plasma into the ISF (NFR_{Body}) and of lymphatic flow (J_{lymph_avg})

across the entire vasculature. Lymphatic flow empties into the venous bed which is cycled through cardiovascular and pulmonary modules, and the systemic circulation.

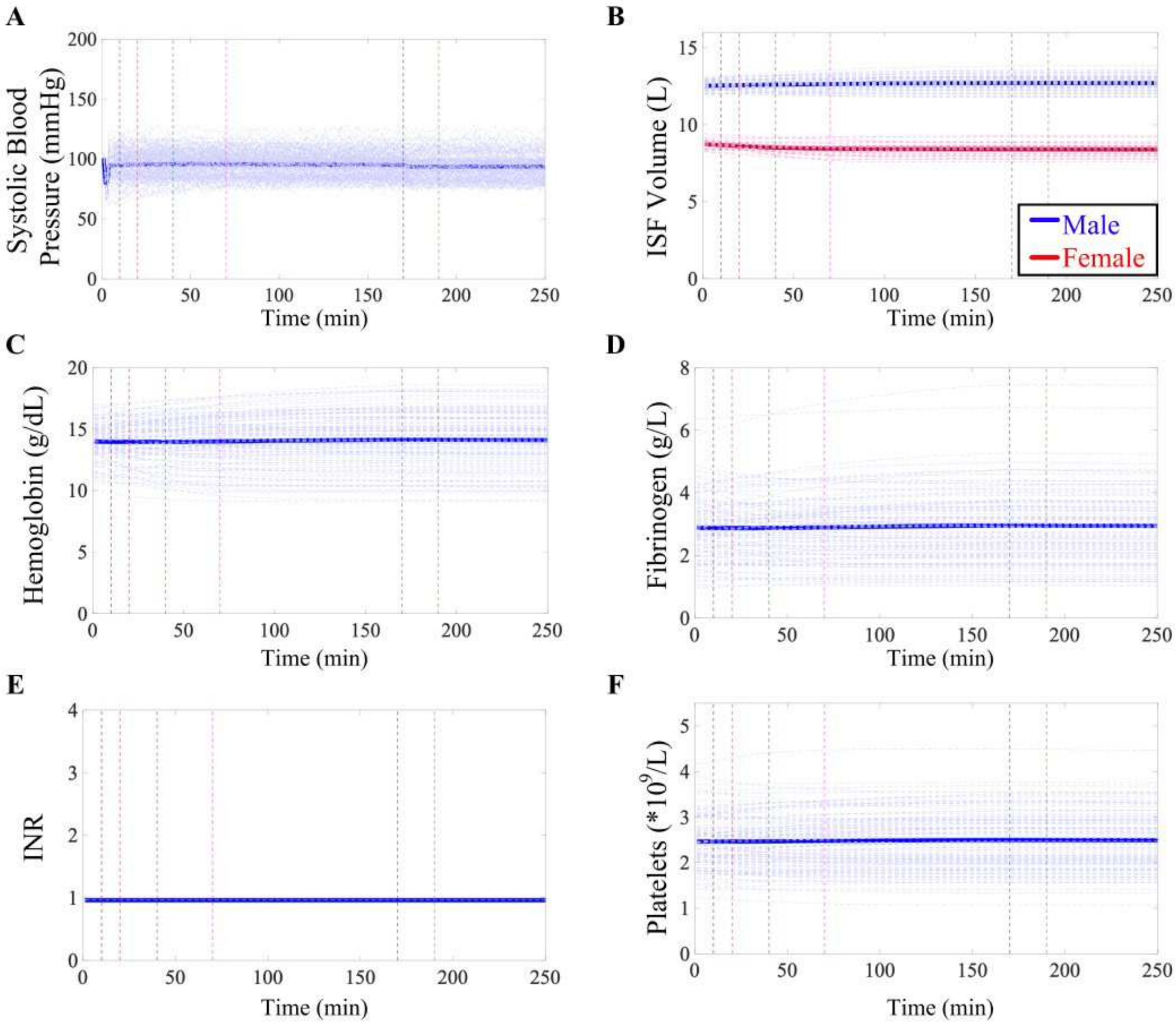

Figure S13:

The simulations of 100 stochastically generated patients who *did not* receive blood products and *did not* experience bleeding. On average the (A) SBP, (B) ISF, (C) Hemoglobin, (D) Fibrinogen, (E) INR, and the (F) platelet count are stable over the course of the simulation. In each graph, a simulation for a single patient is shown as a semi-transparent, dashed line, with the mean value for all patients shown as a solid, opaque line.

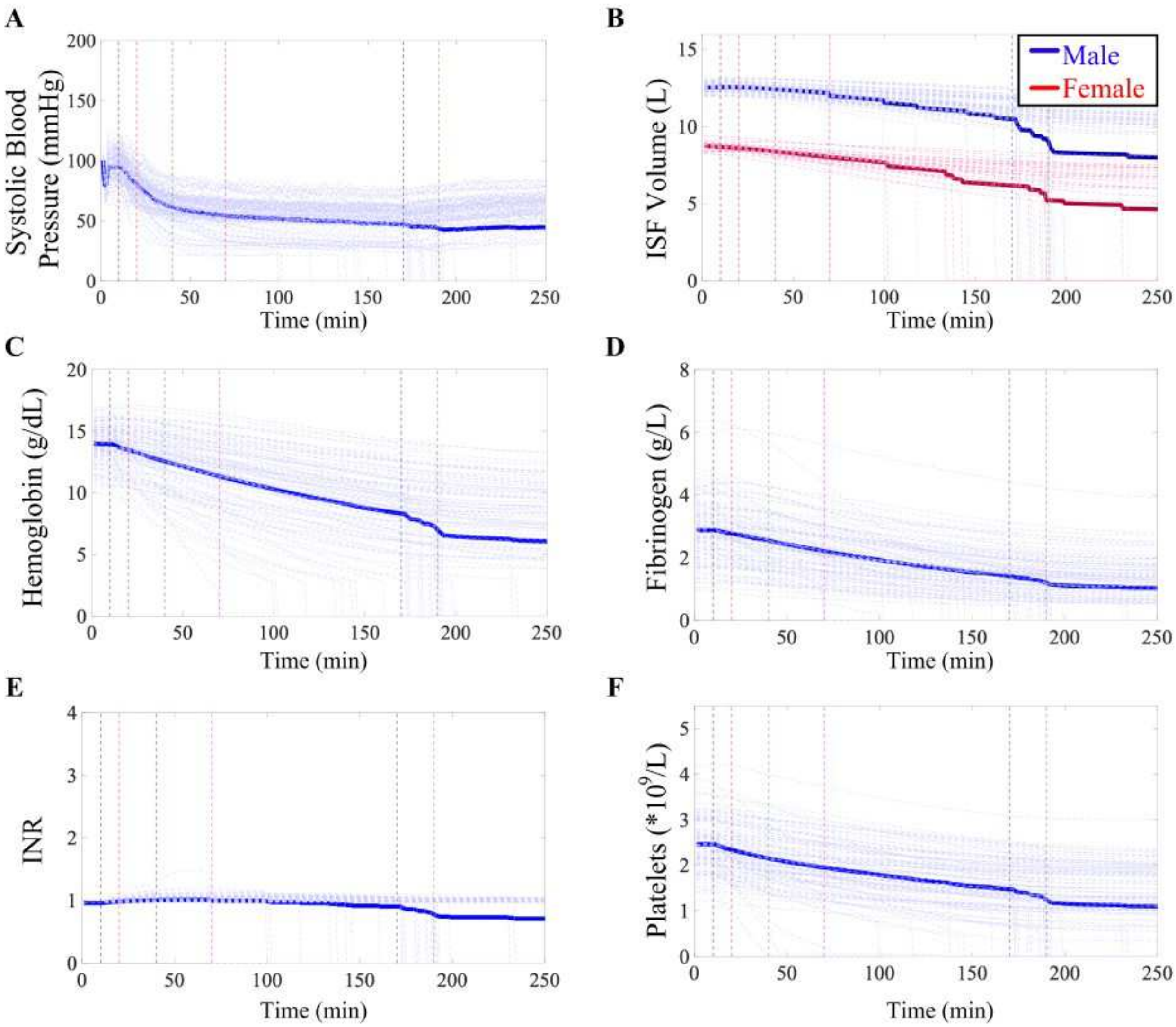

Figure S14:

The simulations of 100 stochastically generated patients who *did not* receive blood products and *did* experience bleeding. The (A) SBP, (B) ISF, (C) Hemoglobin, (D) Fibrinogen, (E) INR, and the (F) platelet count are shown over the course of the simulation. In each graph, a simulation for a single patient is shown as a semi-transparent, dashed line, with the mean value for all patients shown as a solid, opaque line.

Table S1: Simulation results and number of blood products utilized for comparisons of low titer whole blood (LTOWB) *versus* conventional component therapy (CCT) resuscitation and goal-directed therapy (GDT) resuscitation.

Table S1: Simulation results and number of blood products utilized for comparisons of low titer whole blood (LTOWB) versus conventional component therapy (CCT) resuscitation and goal-directed therapy (GDT) resuscitation.

Parameters	Deterministic		ATLS Class III		ATLS Class IV		Goal Directed Therapy (Class III & IV)
	LTOWB	CCT	LTOWB	CCT	LTOWB	CCT	
**A:** Patient Results							
Event free survival	1	1	92	92	78	75	92
Incidence of event*	--	--	8	8	22	25	8
Cardiac Index < 1.5 (L/min/m²)	--	--	8	8	9	4	0
Hemoglobin < 3 g/dL	--	--	0	0	0	0	0
Volume overloaded	--	--	0	0	13	21	8
**B**: Hemostatic level during simulation (IQR)							
Hemoglobin nadir (g/dL)	6.38	6.45	9.21 (2.21)	8.91 (2.63)	5.76 (3.59)	6.12 (2.81)	9.76 (2.89)
Maximum INR	2.27	6.25	1.3 (0.17)	1.64 (0.35)	1.75 (0.25)	3.1 (3.54)	3.59 (4.38)
Fibrinogen nadir (mg/dL)	138.68	128.05	149.94 (68.75)	139.39 (66.28)	107.95 (43.41)	100.71 (65.27)	88.22 (42.54)
PLT count nadir (x 10^9/L)	100.58	107.61	147.66 (50.25)	122.37 (42.17)	73 (78.47)	17.1 (46.38)	79.19 (53.95)
Maximum Tissue Lactate (mmol/L)	2.2	2.2	2.3 (0.85)	2.41 (0.94)	6.32 (7.84)	4.73 (6.92)	2.32 (2.35)
**C:** Critical interval (minutes; IQR)							
Total time in Critical Intervals	219.32	217.12	90.59 (176.76)	147.67 (124.53)	217.57 (44.92)	219.05 (20.69)	160.28 (103.33)
Hgb < 8 g/dL	219.32	172.53	75.09 (182.25)	110.83 (171.11)	212.93 (58.86)	214.63 (15.15)	42.65 (140.23)
INR ≥ 1.5	26.65	78.78	0	0	0	81.85 (113.1)	45.53 (29.3)
Fibrinogen < 150 mg/dL	63.03	79.18	1.34 (78.28)	46.27 (78.28)	131.38 (148.31)	178.24 (148.31)	120.73 (123.46)
PLT count < 50 *10^9/L	0	0	0	0	0	0.12 (0.15)	0
Time in O2 debt	0	0	0	0	0 (77.52)	0 (18.4)	0
**D:** End of simulation (minute 250)							
SBP (mmHg)	77.74	77.74	65.66 (14.9)	65.66 (14.9)	60.22 (23.31)	60.22 (23.31)	96.05 (20.53)
Hemoglobin (g/dL)	9.14	10.66	9.21 (2.07)	9.21 (2.53)	5.81 (3.48)	6.24 (3.28)	12.45 (3.07)
INR	2.27	6.25	1.21 (0.06)	1.25 (0.25)	1.49 (0.4)	1.85 (1.15)	3.45 (4.44)
Fibrinogen concentration (mg/dL)	1.91	2.25	1.61 (0.56)	1.42 (0.66)	1.3 (0.53)	1.12 (0.54)	1.71 (0.88)
PLT count (x 10^9/L)	146.53	133.24	149.07 (48.45)	122.37 (42.17)	76.1 (76.28)	18.35 (47.93)	96.42 (45.84)
Plasma Volume (ml)	3483	3424	3145 (543)	3200 (453)	3368 (628)	3463 (542)	3119 (660)
ISF Volume (ml)	12294	15422	11748 (3336)	12393 (3668)	11100 (3309)	11579 (3714)	12821 (3046)
Tissue Lactate (mmol/L)	2.2	2.2	2.2 (0)	2.2 (0)	2.2 (0.1)	2.2 (0.1)	2.2 (0)
**E:** Number of blood product units given							
Whole Blood	18	0	6	0	12	0	2
Red Blood Cells	0	18	0	6	0	12	8 (2)
Plasma	0	18	0	6	0	12	5 (4)
Platelets	0	3	0	1	0	2	0

*Event defined as: 10 minute average of cardiac index < 1.5 or hemoglobin < 3 g/dL (see Fig. S10 for complete distribution); CCT: Conventional component therapy; LTOWB: low titer group O whole blood; SBP: Systolic blood pressure; ISF: Interstitial fluid; INR: International normalized ratio; IQR: Interquartile range

Table S2: Simulation results and number of blood products utilized in simulations comparing hidden bleed and consumptive coagulopathy parameters and fully stochastic parameters.

Table S2: Simulation results and number of blood products utilized in simulations comparing hidden bleed and consumptive coagulopathy parameters and fully stochastic parameters.

Parameters	Deterministic				Stochastic	
	Hidden Bleed Off	Hidden Bleed On	Consumptive Coagulopathy Off	Consumptive Coagulopathy On	LTOWB	CCT
**A:** Patient Results						
Event free survival	1	--	1	1	89	90
Incidence of event*	--	1	--	--	11	10
Cardiac Index < 1.5 (L/min/m²)	--	--	--	--	8	5
Hemoglobin < 3 g/dL	--	1	--	--	0	0
Volume overloaded	--	--	--	--	3	5
**B**: Lowest hemostatic level during simulation						
Hemoglobin nadir (g/dL)	4.08	2.71	4.08	3.53	8.56 (3.43)	8.47 (3.35)
Maximum INR	1.92	1.82	1.92	2.89	1.34 (0.31)	1.68 (0.67)
Fibrinogen nadir (mg/dL)	87.87	75.67	87.87	43.23	140.44 (72.55)	127.36 (79.08)
PLT count nadir (x 10^9/L)	66.95	30.39	66.95	54.35	141.65 (70.63)	111.73 (55.64)
Maximum Tissue Lactate (mmol/L)	2.86	10.64	2.2	2.2	5.95 (5.99)	3.21 (6.38)
**C:** Critical interval (minutes)						
Total time in Critical Intervals	219.32	104.63	219.32	221.4	129.61 (163.49)	167.73 (131.22)
Hgb < 8 g/dL	219.32	104.63	219.32	221.4	97.34 (199.51)	134.24 (198.98)
INR ≥ 1.5	0	0	0	74.55	0	0 (27.42)
Fibrinogen < 150 mg/dL	192.47	93.77	192.47	215.5	36.87 (120.2)	68.33 (120.2)
PLT count < 50 *10^9/L	0	0.02	0	0	0	0
Time in O2 debt	76.52	18.95	76.52	0	0 (0.52)	0
**D:** End of simulation (minute 250)						
SBP (mmHg)	61.39	33.66	61.39	64.25	64.21 (16.81)	64.21 (16.81)
Hemoglobin (g/dL)	4.08	2.71	4.08	3.53	8.56 (3.51)	8.59 (3.19)
INR	1.53	1.74	1.53	1.82	1.22 (0.11)	1.37 (0.35)
Fibrinogen concentration (mg/dL)	0.88	0.76	0.88	0.43	1.52 (0.65)	1.34 (0.69)
PLT count (x 10^9/L)	66.95	30.39	66.95	54.35	142.5 (70.97)	112.07 (55.64)
Plasma Volume (ml)	4004.37	3088.4	4004.37	4214.02	3179 (552)	3231 (439)
ISF Volume (ml)	10903.63	10555.8	10903.63	11486.92	11569 (3379)	12198 (3816)
Tissue Lactate (mmol/L)	2.43	2.2	2.2	2.2	2.2 (0)	2.2 (0)
**E:** Number of blood product units given						
Whole Blood	2	2	2	2	6 or 18	0
Red Blood Cells	6	6	6	6	0	6 or 18
Plasma	6	6	6	6	0	6 or 18
Platelets	1	1	1	1	0	1 or 3

*Event defined as: 10 minute average of cardiac index < 1.5 or hemoglobin < 3 g/dL (see Fig. S10 for complete distribution); CCT: Conventional component therapy; LTOWB: ow titer group O whole blood; SBP: Systolic blood pressure; ISF: Interstitial fluid; INR: International normalized ratio

Table S3: Time points used in this study's multicompartment stochastic model of a massively bleeding adult patient.

Table S3: Time points used in this study's multicompartment stochastic model of a massively bleeding adult patient.

Phase of Simulation	Length of time (minutes)	Start of phase (minutes)	End of phase (minutes)
Script Initialization	2	0	2
Equilibration B	3	3	5
Equilibration C	5	6	10
Start of Bleeding	10	11	20
Length of Constant Bleeding	5	11	15
Prehospital	20	21	40
Emergency Room (ER)	30	41	70
Operating Room (OR)	120	71	190
Surgical Hemostasis		171	170
Recovery phase	60	191	250
End of simulation			250
Number of timesteps (dt) per cycle of simulation	500		
Frequency of O2 module	0.5		

Table S4: Stochastic patient parameters for patients' physical characteristics and laboratory values.

Table S4: Stochastic patient parameters for patients' physical characteristics and laboratory values.

Parameter	Value	Standard Deviation	Initial Assumption	Reference
**Age Range**	*Percent Chance of Selection (%)*		NA	U.S. Census Bureau, 2020
20-24	6.5			
25-29	7.1			
30-34	6.8			
35-39	6.6			
40-44	6.1			
45-49	6			
50-54	6.1			
55-59	6.5			
60-64	6.5			
65-69	5.4			
70-74	4.5			
75-79	3.1			
80-84	2			
≥85*	1.8			
**Sex**	*Percent Chance of Selection (%)*		Male*,†	
≤50 years old				Clayton *et al.*, 2025
Male	79.7			
Female	20.3			
>50 years old				
Male	72.4			
Female	27.6			
**Weight (kgs)**			70*	
Male ≤39	89.3	1.4		Fryar *et al.*, 2018
40-59	91.1	0.9		
≥60	88.3	0.8		
Female ≤39	76	0.8		
40-59	80	1.4		
≥60	75.5	1.2		
**Minimum weight**	36 kg		–	This study
**Height**			–	
Male ≤39	176.1	0.3		Fryar *et al.*, 2018
40-59	175.8	0.4		
≥60	173.4	0.5		
Female ≤39	162.7	0.4		
40-59	162.1	0.4		
≥60	159.3	0.5		
Correlation Coefficient for Height and Weight (ρ)				Silverman, 2022
Male	0.4716			
Female	0.5387			
**Systolic Blood Pressure (SBP)**	Randomized through a normal distribution of 2.5% of CBV		–	This study
**Laboratory Values**				
Hematocrit (%)			44*	
Male		4.3		Cheng *et al.*, 2004
18-25	45.8			
26-35	44.1			
36-45	44			
46-55	44			
56-65	43.7			
66-75	43.3			
>75	42.1			
Female		4.3		
18-25	38.8			
26-35	39.4			
36-45	39.8			
46-55	39.7			
56-65	39.7			
66-75	40.2			
>75	39.7			
Fibrinogen (g/dL)	2.93	0.88	3†	Unpublished data from UPMC Presbyterian Hospital
INR	1	0.1	1†	
Platelet Count (x 10^9/L)	239180	61811	230†	
ISF Protein Concentration (g/dL)	Random value between 1.4-2.6		2.0	Mayo Clinic Test Catalog; Killingsworth, 1979; Tietz textbook of Clinical Chemistry, 2018
Serum Protein Concentration (g/dL)	Random value between 6.3-7.9		7.3	Smith & Staples, 1982
**Heart Rate**			80*	
Initialization	80	0		Jamal *et al.*, 2014
Equilibration & after surgical hemostsis	91.4	19.2		Brasel *et al.*, 2007
Frequency of fluctuations	30 seconds			User Defined
Max allowed HR	220-Age			CDC, 2022
Change in Heart Rate as interpolation of:				
Percent of Blood Loss (% loss)	[15, 30, 40]			ATLS Guidelines, 10th edition
Heart Rate	[100, 120, 140]			ATLS Guidelines, 10th edition
Percent of Blood Loss > 40%	HR = 1.579*(%loss) + 75.26			Cooke *et al.*, 2006

*: Original assumption from Jamal et al., 2015

†: Original assumption from Scheult *et al.*, 2019

CBV: Calculated Blood Volume

Table S5: Stochastic values for the parameters for ATLS class, bleeding rate, the "hidden bleed" parameter, and consumptive coagulopathy parameters.

Table S5: Stochastic values for the parameters for ATLS class, bleeding rate, the "hidden bleed" parameter, and consumptive coagulopathy parameters.

Parameter	Value	Initial Assumption	Reference
**Trauma Severity (ATLS class) & Blood Volume Loss (%)**	Randomly Select Value between:	--	ATLS Guidelines, 10th edition & Mutschler *et al.*, 2013
ATLS Class I (62.4% of patients)†	0-15	--	
ATLS Class II (18.7% of patients)†	15-30	--	
ATLS Class III (6.0% of patients)	30-40	--	
ATLS Class IV (1.8% of patients)	≥40	--	
**Bleeding Rate**	CBV(1) * % Blood Volume loss (during defined constant bleeding rate)		User Defined
Initial Bleeding Rate		135 ml/min	Seheult *et al.*, 2019
Bleeding Rate as function of Hemostatic Factor	J_bleed = (SBP/(SBP_initial)) * HemostaticFactor		Seheult *et al.*, 2019
Bleeding Rate as function of R_bleed‡	J_bleed(t)=(Pbnch_tot(t))/R_bleed	50ml/min	Jamal *et al.*, 2014
R_bleed (mmHg*sec*ml^-1)	--	24	Jamal *et al.*, 2014
Time of Physilogically Uncontrolled Bleeding (minutes)	5	--	This study
**Hidden Bleed**	Percent chance of having hidden bleed (%)	--	ATLS Guidelines, 10th edition & Mutschler *et al.*, 2013
ATLS Class I	0.9	--	
ATLS Class II	1.3	--	
ATLS Class III	22.6	--	
ATLS Class IV	34.9	--	
**Consumptive Coagulopathy (CCg)**	Percent chance of having coagulopathy (%)		
ATLS Class II	1	--	This study
ATLS Class III	5	--	
ATLS Class IV	25	--	
**Fibrinogen Consumption Rate (mg/min)**		60	Seheult *et al.*, 2019
ATLS Class II	0-5	--	This study
ATLS Class III	5-10	--	
ATLS Class IV	10-20	--	
Coagulopathy	20-60	--	
**Plasma Consumption Factor (ml/min)**		45	Seheult *et al.*, 2019
ATLS Class II	0-2.5	--	This study
ATLS Class III	2.5-6	--	
ATLS Class IV	6-10	--	
Coagulopathy	10-15	--	
**Platelet Consumption Factor (platelets/min)**		2*10^10	Seheult *et al.*, 2019
ATLS Class II	0-2 * 10^9	--	This study
ATLS Class III	2-5 * 10^9	--	
ATLS Class IV	5-8 * 10^9	--	
Coagulopathy	8-10 * 10^9	--	
**Red Blood Cell Consumption Factor (ml/min)**		15	Seheult *et al.*, 2019
ATLS Class II	0-2	--	This study
ATLS Class III	2-5	--	
ATLS Class IV	5-10	--	
Coagulopathy	10-15	--	

†Not included in stochastic calculation, but can be selected; ‡: Not included in current implementation

CBV: Calculated blood volume

Table S6: Stochastic parameters and key equations for simulating transfused blood products.

Table S6: Stochastic parameters and key equations for simulating transfused blood products.

Parameter	Value	Standard Deviation	Initial Assumption*	Reference
Low Titer Group O Whole Blood (LTOWB)				
Product Volume (V_WB)	500 + Volume Variance		570	This study
Total Volume Variance	22.4		NA	This study
Hematocrit of donor (Hct_donor)	41.8	4.3	45	This study
RBC volume (ml)	V_WB * Hct		228	This study
Plasma volume (ml; Plasma_WB)	V_WB * (1-Hct)		272	This study
Hemoglobin	V_WB * (Hct/3)		75	This study
CPD (ml; CPD_WB)	70		70	
Fibrinogen	3.11	0.47	3.11	Seheult et al., 2019
Platelets (*10^11)	0.99	0.3	1.00	Seheult et al., 2019
INR	1.11	0.09	1.11	Seheult et al., 2019
Prothrombin Time (PT)	INR^(1/1.07))*10.8		10.4	Seheult et al., 2019
Plasma Protein Concentration (g/dL)	Random value between 6 and 8 * (V_WB/(W)WB + V_CPD))		--	Barrett et al., 2010
Red Blood Cells				
Product Volume	--		378	
Total Volume Variance	22.4		--	
Final Hematocrit of RBC unit (Hct_rbc)	75.0	4.0		Zuck *et al.*, 1977
RBC Volume (ml; V_RBC)	(Hct_donor * V_WB) / Hct_rbc		228	
CPD Percent of Total Plasma Volume of in Final Unit (%)	20		20	
Plasma Volume in RBC (ml; Plasma_RBC)	0.8* (V_WB -V_RBC)		32	
Hemoglobin in RBC	V_RBC * (Hct/3)		75	
Additive Solution	110		110	
Protein Concentration of Plasma ***in RBC unit*** (g/dL)	Random value between 6 and 8 * (V_plasma/(V_plasma + V_CPD))		--	Barrett, et al., 2010
Plasma				
Product Volume (V_Plasma)	--		234	
Total Volume Variance	22.4		--	
Final Hematocrit of RBC unit (Hct_rbc)	75.0	4.0		Zuck *et al.*, 1977
RBC Volume (ml; V_RBC)	(Hct_donor * V_WB) / Hct_rbc		--	
CPD Percent of Total Plasma Volume of in Final Unit (%)	20		20	
Plasma Volume in RBC (ml; Plasma_RBC)	0.8* (V_WB -V_RBC)		--	
Plasma Volume *in Plasma Unit*	Plasma_WB - Plasma_RBC			
Platelet Volume *in Plasma Unit* (ml; Plasma_PLT)	69.3	2.5	68	
CPD Percent of Total Platelet Volume of in Final Unit (%)	20		20	
CPD volume in Platelets (ml; CPD_PLT)	0.2 * Plasma_PLT		--	
CPD volume *in Plasma Unit* (ml; CPD_Plasma)	CPD_WB - CPD_in_RBC - CPD_PLT			
Fibrinogen	3.11	0.47	PlasmaTransfusionRate * 3	
INR	1.11	0.09		
Prothrombin Time (PT)	INR^(1/ISD))*10.8			
Protein Concentration of Plasma ***in Plasma unit*** (g/dL)	Random value between 6 and 8 * (V_plasma/(V_plasma + V_CPD))		--	Barrett, et al., 2010
Platelets				
Total Platelet Unit Volume (ml; V_PLT)	69.3	2.5	68	
Total Volume Variance	22.4		--	
CPD Percent of Total Plasma Volume of in Final Unit (%)	20		20	
Plasma Volume in RBC (ml; Plasma_RBC)	0.8* (V_WB -V_RBC)		32	
Plasma Volume *in Plasma Unit (Plasma_Plasma)*	Plasma_WB - Plasma_RBC			
Platelet Volume in Plasma Unit (ml; Plasma_PLT)	69.3	2.5	68	
CPD Percent of Total Platelet Volume of in Final Unit (%)	20		20	
CPD volume in Platelets (ml; CPD_PLT)	0.2 * Plasma_PLT		--	
Platelet Count	0.99	0.3	1.0*10^11	
Protein Concentration of Plasma ***in Plasma*** (g/dL)	Random value between 6 and 8 * (V_plasma/(V_plasma + V_CPD))		--	Barrett, et al., 2010

CPD: Citrate-Phosphate-Dextrose; RBC: Red Blood Cell; INR: International Normalized Ratio

* From Seheult et al., 2019 unless otherwise specified

Table S7: Key equations governing patients' physical and laboratory characteristics.

Table S7: Key equations governing patients' physical and laboratory characteristics.

Parameter	Symbol	Unit	Equation	Initial Assumptions	Reference
Patient Characteristics					
Body Mass Index	BMI	$\frac{kg}{m^2}$	–	++	Nadler *et al.*, 1962
BMI $\leq$ 30 (Male)	–	–	$0.3669 \cdot (\text{height})^3 + 0.03219 \cdot \text{weight} + 0.6041$	++	–
BMI $\leq$ 30 (Female)	–	–	$0.3561 \cdot (\text{height})^3 + 0.03308 \cdot \text{weight} + 0.1833$	++	–
BMI $\geq$ 30	–	–	$\frac{70}{\sqrt{\frac{\text{BMI}}{22}}} \cdot \text{weight}$	++	Lemmens *et al.*, 2006
Body Surface Area	BSA	m^2	$0.007184 \cdot \text{weight}^{0.425} \cdot \text{height}^{0.725}$	++	Dubois, 1916
Total Body Water	TBW	L		$\text{TBW} = 0.6 \cdot \text{weight} \cdot 1000$	Hume & Weyes, 1971
Male	–	–	$0.194786 \cdot \text{height} + 0.296785 \cdot \text{weight} - 14.012934$		
Female	–	–	$0.344546 \cdot \text{height} + 0.183809 \cdot \text{weight} - 35.270121$		
Intracellular Fluid Volume	ICF	mL	**	$\frac{2}{3} \cdot \text{TBW}$	–
Red Blood Cell Fluid	RBCF	mL	**	$70 \cdot \text{weight} \cdot \text{hematocrit}$	–
Non-RBC Intracellular Fluid	NRBCF	mL	**	ICF − RBCF	–
Extracellular Fluid Volume	ECF	mL	**	$\frac{1}{3} \cdot \text{TBW}$	–
Plasma Volume	Vpl	mL	**	$70 \cdot \text{weight} \cdot (1 - \text{hematocrit})$	–
Plasma Compartment	–	mL	**	$70 \cdot \text{weight} \cdot (1 - \text{hematocrit})$	–
Interstitial Fluid Volume	ISF	mL	**	ECF − Vpl	–
Osmolarity	–	$\frac{\text{mOsm}}{\text{ml}}$	**	0.3	–
PV dextrose osmoles	–	mOsm	$80 \frac{mg}{dL} \cdot \frac{V_{pl}}{100 \cdot \text{MW of dextrose}}$	13.0	–
Hemoglobin	Hb	g/dL	**	$\frac{\text{Hct}}{3}$	–
MCHC	MCHC	g/dL	$\frac{\text{Hb}_{\text{level}}}{\text{Hct}}$	++	–

**: Current model is the same as previous model's assumptions
++: Not implemented in original model

Table S8: Key equations describing the heart module and vessel parameters.

Parameter	Symbol	Unit	Equation/Value	Initial Assumption	Reference
Heart Module					
Seconds per Beat per Heart Cycle	dt	–	$\frac{1}{N}$	$\frac{1}{N} \cdot \frac{60\,\text{sec}}{\text{min}} \cdot \frac{1}{\text{HR}}$	
Maximum time of the heart cycle period	t_{max}	–	$\text{round}(\frac{60sec/min}{\text{HR}} \cdot N)$	$\frac{400-1.8 \cdot \text{HR}}{\text{SecPerBeatPerCycle}}$	
Delay between Atrial Activation and SA Node Firing (PR interval)	delay	sec			
LV & RV	–	–	–	$\frac{0.160 * N}{\text{sectomin}/\text{HR}}$	
Male	–	–	$\text{round}(-0.52 \cdot \text{HR} + 209)$		Lee *et al.*, 1995
Female	–	–	$\text{round}(-0.43 \cdot \text{HR} + 207)$		Lee *et al.*, 1995
LA & RA	–	–	0	0	
Heart Chamber Size					Pfaffenberger *et al.*, 2013
Left Ventricle End Diastolic Diameter	$\text{LV}_{\text{EDD-Vol}}$	ml	$-39.37731+0.73703 \cdot \text{height}-9.47838 \cdot \text{sex}-0.33895 \cdot \text{age}+0.42808 \cdot \text{weight}$	120	
RV End Diastolic Diameter	RV_{EDD}	mm	$15.94580 + 0.07014 \cdot \text{height} - 2.35620 \cdot \text{sex} + 0.04375 \cdot \text{weight}$	++	
RV Area	RV_{Area}	cm^2	$-0.97314+0.1004 \cdot \text{height}-2.62433 \cdot \text{sex}-0.03906 \cdot \text{age}+0.09270 \cdot \text{weight}$	++	
RV Volume*	RV_{Vol}	ml	$0.85 \cdot \frac{\text{RV}_{\text{Area}}^2}{\text{RVEDD}}$	20	
LA Volume	LA_{Vol}	ml	$-34.73810 + 0.31554 \cdot \text{height} + 0.10538 \cdot \text{age} + 0.21533 \cdot \text{weight}$	20	
RA Volume	RA_{Vol}	ml	$18.97912 - 8.55635 \cdot \text{sex} + 0.04375 \cdot \text{weight}$	120	
Regression Line of SBP with Age	AgeElastanceModifier	–		–	Franklin *et al.*, 1997
Male	–	–	0.47	++	
Female	–	–	0.62	++	
Age Corrected Elastance (E)	$\frac{mmHg}{ml}$	–	$\text{E}_{branch} \cdot \frac{\text{SBP}+\text{SBP}_{\text{Regression Line}} \cdot (\text{Age}-30)}{\text{SBP}}$	++	
Chamber Volume Correction Term	$\beta_{\text{heart ID}}$	–	$\frac{\text{Original Chamber Vol}}{\text{Stochastic Chamber Vol}}$	++	
Unfilled Heart Chamber Volume	V_0	ml			Barnea & Sheffer, 1993
LV	–	–	$\frac{15}{\beta_{heartID}}$	15	
RV	–	–	$\frac{50}{\beta_{heartID}}$	25	Barnea & Sheffer, 1993
LA & RA	–	–	$\frac{5}{\beta_{heartID}}$	0	
"Unstressed" Venous Volume	$\text{V}_{0,\text{vein}}$	ml	$\text{weight} \cdot (20.2 \pm 1.0)$	1750	Rothe *et al.*, 1983 & Sheldon *et al.*, 1998

Continued on the next page

Parameter	Symbol	Unit	Equation	Initial Assumption	Reference
(Continued from the previous page)					
Normalized Elastance	E_N	–	$E_{nor} = E_N[3] \cdot t_N^3 + E_N[2] \cdot t_N^2 + E_N[1] \cdot t_N$	$E_{nor} = E_N[3] \cdot t_N + E_N[2] \cdot t_N + E_N[1] \cdot t_N$	Barnea & Sheffer, 1993
LA, RA	–	–	[0.158, 2.685, -1.841]	[0.158, 2.685, -1.841]	
LV	–	–	[0.158, 2.685, -1.841]	[0.158, 2.685, -1.841]	
RV	–	–	[-1.934, 6.568, -3.374]	[0.158, 2.685, -1.841]	
Valve Resistances	R_v	$mmHg \cdot sec \cdot ml^{-1}$			
Aortic Valve	–	–	**	0.0004	
Tricuspid Valve	–	–	**	0.0004	
Pulmonic Valve	–	–	**	0.0004	
Mitral Valve	–	–	**	0.004	
Pulmonary Segment Resistances	R_{Pulm}	$\frac{mmHg \cdot sec}{ml}$	[0.01, 0.055, 0.025]	[0.267, 0.11, 0.004]	Barnea & Sheffer, 1993
Diastolic function	D	–			Barnea & Sheffer, 1993
LV	–	–	**	[0.0375, 0.1525, 7, 0.025]	
RV	–	–	**	[0.06, 0.025, 7, 0.025]	
LA & RA	–	–	**	[0.15, 0.09, 7, 0.09]	
Scaling factor for heart valve resistance as a function of chamber pressure	Kv	–	**	[0.0003, 0.0003, 0, 0]	Jamal *et al.*, 2014
Lymph Flow Scaling Factor		–	$9.7922 \cdot 10^{-11}$	$9.7922 \cdot 10^{-12}$	Himeno *et al.*, 2015
Relative viscosity factor (μ_r)		–	$\mu_r = 0.31855 \cdot \exp(2.6 \cdot Hct)$		Barnea & Sheffer, 1993

**:Current model is the same as previous model's assumptions
++: Not implemented in original model
HR: Heart Rate; LV: Left Ventricle; RV: Right Ventricle; LA: Left Atrium; RA: Right Atrium; LVEDD: LV End Diastolic Diameter; RVEDD: RV End Diastolic Diameter (in mm).
*RV volume is not routinely calculated; area-length methods are used to calculate RV volume. See Ostenfeld & Flachskampf, 2015 for further discussion.

Table S9: Equations describing the capillary filtration module.

Parameter	Symbol	Unit	Equation	Initial Assumptions
*Filtration Across the capillary parameters**				
Net Filtration Rate ‡	$j_{v,C}$	$\frac{\mu L}{ms \cdot mm}$	$NFR = K * ((P_{pl} - P_{isf}) - (\Pi_{pl} - \Pi_{isf})$	$NFR = \frac{1}{R_{bleed}} * ((P_{art} - P_{vein}) - (\Pi_{pl} - \Pi_{isf})$
Resistance to Filtration†	R_{bleed}	$\frac{mmHg \cdot min}{ml}$	++	25
Serum conductivity	K	$\frac{\mu L}{ms \cdot mmHg \cdot mm}$	$K = \frac{V_C*(1-H_C)*\frac{1}{200}}{\frac{L}{2\pi r}*8} * \frac{2}{L}$	
Interstitial fluid pressure	P^0_{isf}	mmHg	$P^0_{isf} = f_1(V_{isf}(t)) = -3.139 \cdot e^{\frac{1-\frac{V_{isf}(t)}{V_{0_{isf}}}}{0.12}} + 3.61 \cdot \frac{V_{isf}(t)}{V_{0_{isf}}}$	
Plasma Axial Hydrostatic Pressure	P_{pl}	mmHg	$p_{pl}(x_i) = P_{A,branchavg} + (P_{V_{avg}} - P_{A,branchavg}) \cdot \frac{x_i}{L}$	**
Axial Plasma Volume	v_{pl}	ml	$\frac{\partial v_{pl}}{\partial t} = \frac{v_{pl}(t-1,x_i)-v_{pl}(t-1,x_{i-1})}{\Delta z} \cdot v_{flow} - j_{v,C}(t,x_i)$	**
Integrated Volume fluxes	$J_{v,C}$	$\frac{ml \cdot cm}{sec}$	$J_{v,C}(t) = \frac{1}{L} \times \sum_{i=1}^{N}(j_{v,C}(t,x_i) \times \Delta x)$	**
Quantity of Protein in Plasma	q_{pl}	g	$\frac{\partial q_{pl}}{\partial t} = -\frac{q_{pl}(t-1,x_i)-q_{pl}(t-1,x_{i-1})}{\Delta z} \cdot v_{flow} - j_{q,C}(t,x_i)$	**
Initial Plasma Colloids Concentration §	C_{pl}	g/dL	6.9 - 7.9	7.3‡
Initial Interstitial colloids concentration §	C_{pl}	g/dL	1.4 - 2.6	2.0‡
Concentration of Protein in Plasma §	C_{pl}	g/dL	$c_{pl}(t,x_i) = \frac{q_{pl}(t,x_i)}{v_{pl}(t,x_i)}$	$C_{pl}(t) = \frac{Q_{pl-inf}(t)+Q_{pl-bld}(t)}{V_{pl}(t)/100}$
Colloidal Osmotic Pressure of Plasma §	π_{pl}	mmHg	$\pi_{pl}(t,x_i) = 0.157 \times \frac{q_{pl}(t,x_i)}{v_{pl}(t,x_i)} + 3.2 \cdot 10^{-3} \times \left(\frac{q_{pl}(t,x_i)}{v_{pl}(t,x_i)}\right)^2$	$0.2274 * C_{pl}(t)^2 + 2.1755 * C_{pl}(t)$
Colloidal Osmotic Pressure of ISF	Π_{ISF}	mmHg	$\Pi_{ISF}(t) = 0.157 \times \frac{Q_{ISF}(t)}{V_{ISF}(t)} + 3.2 \cdot 10^{-3} \times \left(\frac{Q_{ISF}(t)}{V_{ISF}(t)}\right)^2$	$0.2274 \cdot C_I(t)^2 + 2.1755 * C_I(t)$
Protein flux through large pore system	$j_{q,C}$	$\frac{\mu g}{ms \cdot mm}$		**
Filtration			$j_{q,C}(t,x_i) = G_p \times (c_{pl}(t,x_i) - C_{ISF}(t)) + j_{v,C}(t,x_i) \times c_{pl}(t,x_i)$	
Reabsorption			$j_{q,C}(t,x_i) = G_p \times (c_{pl}(t,x_i) - C_{ISF}(t)) + j_{v,C}(t,x_i) \times c_{pl}(t,x_i)$	
Integrated Protein Flux	$J_{q,C}$	$\frac{g \cdot cm}{sec}$	$J_{q,C}(t) = \frac{1}{L} \times \sum_{i=1}^{N}(j_{c,C}(t,x_i) \times \Delta x)$	**
Integrated Lymph Protein Flux	$J_{q,LF}$	$\frac{g \cdot cm}{sec}$	$J_{q,LF}(t) = J_{v,LF}(t) \times C_{ISF}(t)$	**
Quantity of Protein in ISF	Q_{ISF}	g	$\frac{\partial Q_{ISF}(t)}{\partial t} = J_{q,C}(t) - J_{q,LF}(t)$	**
Quantity of Protein Lost during Bleeding	Q_{bleed}	g	$Q_{bleed} = c_{pl} * (V_{Bleed}(i) - V_{Bleed}(i-1))$	**
Quantity of Protein Gained during Infusion††	Q_{resus}	g	$Q_{resus} = Q_{CCT} + Q_{WB}$	++
Total Blood Protein (step 1)††	Q_{blood}	g	$Q_{blood} = Q_{blood} + JqC_{avg}(t) \cdot numberofCapillaries \cdot L$	++
Total Blood Protein (step 2)††	$Cpl_{numerator}$	g	$Cpl_{numerator} = Q_{blood} + Q_{resus} - Q_{bleed}$	++
Total Blood Protein (step 3)††	Cpl_{body}	g	$\frac{Cpl_{numerator}}{V_{plasma}}$	++

*: Himeno, *et al.*, 2016 unless otherwise specified;
†: Jamal *et al.*, 2014;
‡: Starling, 1896;
§: See Table S2 for reference
††: This study
**: Current model is the same as previous model's assumptions
++: Not implemented in corresponding model

Table S10: Equations describing the capillary and tissue resistances from the oxygenation module.

Table S10: Equations describing the capillary and tissue resistances from the oxygenation module.

Parameter	Symbol	Unit	Equation	Initial Assumptions
*Oxygenation Module**				
Hemoglobin Oxygen Saturation Curve ‡	$SO_2(P_c(z,t))$		$SO_2(P_c(z,t)) = \frac{PO_2+2a_2(PO_2)^2+3a_3(PO_2)^3+4a_4(PO_2)^4}{4*(1+a_1 PO_2+a_2(PO_2)^2+a_3(PO_2)^3+a_4(PO_2)^4)}$	**
Oxygen content of arterial blood	CaO_2	$CaO_2 ml\ O_2/dL$	$CaO_2 = \alpha_c \cdot PO_{2A} \cdot Hct \cdot MCHC \cdot O_{2Hb} \cdot SaO_2$	**
Cardiac Output	CO	L/min	$CO = HR \cdot SV$	**
Oxygen Delivery	DaO_2	ml/min	$DaO_2 = CO \cdot CaO_2$	**
Tissue capillary O_2 concentration	$Cc(z,t)$	mlO_2/ml	$Cc(z,t) = \alpha_c \cdot Pc(z,t) + Hct \cdot MCHC \cdot SaO_2(Pc(z,t))$	
Tissue capillary O_2 concentration	$Cc(z,t)$	mlO_2/ml	$Cc(z,t) = \alpha_c \cdot Pc(z,t) + Hct \cdot MCHC \cdot SaO_2(Pc(z,t))$	
Capillary O_2 pressure	cap_{press}	mmHg	$Pc(z,t) = f^{-1}(SaO_2(Pc(z,t)) = f^{-1}(\frac{Cc(z,t)-\alpha_t \cdot Pc(z,t)}{Hct \cdot MCHC \cdot O_{2,Hb}})$	§
Capillary†				
Capillary Capacitance	C^i_{cap}		$C^i_{cap} = \pi R_c^2$	**
Resistance of **tissue** segment	$R_t^{i,j-1}$		$R_t^{i,j-1} = R_t^{i,j+1} = \frac{dz}{2Q_c}$	**
Resistance of **capillary** axial segment	R^i_{c-t}		$R^i_{c-t} = \frac{1}{k_t}$	**
Tissue and Alveolus†				
Width of axial segment	W_{Ax}	cm	$W_{Ax} = L/Ax$	
Change in Volume of tissue segment (i,j)	$\Delta V^{i,j}$	ml	$\Delta V^{i,j} = \pi \cdot (r_{pos}(j+1)^2 - r_{pos}(j)^2) \cdot W_{Ax}$	**
Tissue Capacitance	$C_t^{i,j}$		$C_t^{i,j} = \alpha_t \Delta V^{i,j}$	**
Resistance of previous/next **axial** segment	$R_t^{i-1,j}$		$R_t^{i-1,j} = R_t^{i+1,j} = \left(\frac{dz}{2}\right)^2 \cdot \frac{1}{\alpha_c D_c \Delta V^{i,j}}$	**
Resistance of previous **tissue** segment	$R_t^{i,j-1}$		$R_t^{i,j-1} = \left(\frac{dr}{2}\right)^2 \cdot \frac{1}{\alpha_c D_c \Delta V^{i,j}}$	**
Resistance of next **tissue** segment	$R_t^{i,j+1}$		$R_t^{i,j+1} = \left(\left(\frac{dr}{2}\right)^2 + r^{i,j}\left(\frac{dr}{2}\right)\right) \cdot \frac{1}{\alpha_c D_c \Delta V^{i,j}}$	**
Resistance of axial $i+1$ tissue segment	$R^{Ls}_{t_{ij}}$		$R^{Ls}_{t_{ij}} = R^{i-1,j}_{t_i} + R^{i,j}_{t_{i-1}}$	**
Resistance of axial $i-1$ tissue segment	$R^{Rs}_{t_{ij}}$		$R^{Rs}_{t_{ij}} = R^{i+1,j}_{t_i} + R^{i,j}_{t_{i+1}}$	**
Resistance of radial $j+1$ tissue segment	$R^{B}_{t_{ij}}$		$R^{B}_{t_{ij}} = R^{i,j-1}_{t_j} + R^{i,j}_{t_{j-1}}$	**
Resistance of radial $j-1$ tissue segment	$R^{U}_{t_{ij}}$		$R^{U}_{t_{ij}} = R^{i,j+1}_{t_j} + R^{i,j}_{t_{j+1}}$	**
Oxygen Consumption	$M^{i,j,n}$		$M^{i,j,n} = M_{max} \cdot \frac{P_t^{i,j,n}}{k_m + P_t^{i,j,n}} \cdot \Delta V^{i,j}$	**

**: Initial equations/assumptions are the same as current model's equation/assumptions
*: Siam, *et al.*, 2015 unless otherwise specified;
‡ : *Adairet al.*, 1925;
§: The inverse Adair equation was originally modeled per the following polynomial:
$Pc(z,t)) = 7.0624 \cdot C(z)^9 + 52.852 \cdot C(z)^8 + 142.78 \cdot C(z)^7 + 149.52 \cdot C(z)^6 + 2.6376 \cdot C(z)^5 - 81.241 \cdot C(z)^4 - 7.5042 \cdot C(z)^3 + 36.224 \cdot C(z)^2 + 27.711 \cdot C(z) + 37.752$

Table S11: Equations describing the physical parameters underlying the tissue oxygenation module.

Table S11: Equations describing the physical parameters underlying the tissue oxygenation module.

Parameter	Symbol	Unit	Value	Initial Assumption	Reference
Max O2 used in Adair equation	max_{O2}	mmHg	150	150	
Adair Equation Coefficient	–	unitless			Li *et al.*, 1997
a1	–	–	0.01524		
a2	–	–	$7.10 \cdot 10^{-5}$		
a3	–	–	0		
a4	–	–	$2.70 \cdot 10^{-6}$		
Starting capillary blood flow	Q_b	ml/sec	$7.70 \cdot 10^{-9}$	**	Jamal *et al.*, 2015
		mm/s	0.2 – 1.8		Ivanov & Kalinina, 1981
Hemoglobin density	$Hgb_{density}$	g/mL	0.34	**	Jamal *et al.*, 2015; Guyton & Hall, 2006
Hemoglobin O_2 content	$O2_{Hb}$	mlO_2/g	1.34	**	Jamal *et al.*, 2015; Guyton & Hall, 2006
Blood oxygen solubility	α_c	$\frac{mLO_2}{ml \cdot mmHg}$	$3.00 \cdot 10^{-5}$	$3.00 \cdot 10^{-5}$	Sharan *et al.*, 1989
<u>Tissue Module</u>					
Capillary and tissue axial divisions		–	50	**	Jamal *et al.*, 2015
Tissue radial elements	$tissue_{layers}$	–	10	**	Jamal *et al.*, 2015
Capillary radius	R_c	cm	$3.24 \cdot 10^{-4}$	**	Jamal *et al.*, 2015; Sharan *et al.*, 1997; Reneau *et al.*, 1967
Capillary, tissue cylinder length	L	cm	0.03	**	Jamal *et al.*, 2015; Middleman, 1972
Tissue cylinder radius	r_{tissue}	cm	$3.25 \cdot 10^{-3}$	**	Jamal *et al.*, 2015; Sharan *et al.*, 1997
Tissue O_2 diffusivity		cm^2/sec	$2.1 - 2.9 \cdot 10^{-5}$	$1.5 \cdot 10^{-5}$‡	Androjna *et al.*, 2008
Tissue O_2 diffusivity	$PO2_{t-D}$	cm^2/sec	$1.7 \cdot 10^{-5}$	**	Jamal *et al.*, 2015
Tissue O_2 diffusivity	$PO2_{t-D}$	cm^2/sec	$1.5 \cdot 10^{-5}$	**	Sharan *et al.*, 1989
Tissue oxygen solubility	α_t	$\frac{mLO_2}{ml \cdot mmHg}$	$3.00 \cdot 10^{-5}$	**	Sharan *et al.*, 1989
Tissue oxygen diffusion		cm^2/sec	$1.70 \cdot 10^{-3}$	**	Sharan *et al.*, 1989
Mass transfer coefficient	k_t	$\frac{mLO_2}{cm^2 \cdot s \cdot mmHg}$		$4.40 \cdot 10^{-6}$	Eggleton *et al.*, 2000
			$K_t = 1.21 - 4.3{*}Hct{+}23.{*}(Hct)^2 * 10^{-6}$		
Arterial pO2 at start of sim	$PO2_{Astrt}$	mmHg	99.7	100	Jamal *et al.*, 2015; Sharma & Hashmi, 2018
Tissue pO2	$tissue_{PO2}$	mmHg	40	**	Jamal *et al.*, 2015; Otiz-Prado *et al.*, 2015; Beerthuizen *et al.*, 1989; Carreau *et al.* 2011
Tissue capillary O_2 concentration	$Cc(z,t)$	mlO_2/ml	Stochastic‡	0.22	

**: Current model is the same as previous model's assumptions.

‡ Cc(z,t) calculated as $Cc(z,t) = \alpha_c \cdot Pc(z,t) + Hct \cdot MCHC \cdot SaO_2(Pc(z,t))$

Table S12: Equations describing the physical parameters underlying the alveolar oxygenation module and tissue oxygen consumption.

Table S12: Equations describing the physical parameters underlying the alveolar oxygenation module and tissue oxygen consumption.

Parameter	Symbol	Unit	Value	Initial Assumption	Reference
Alveolar Module					
Capillary and alveolus axial divisions	Ax	–	50	**	
Alveolar radial elements	–	–	3	**	
Capillary radius	R_c	cm	$3.24 \cdot 10^{-4}$	**	Jamal *et al.*, 2015; Sharan *et al.*, 1997; Reneau *et al.*, 1967
Capillary/tissue cylinder length	L	cm	0.03	**	Jamal *et al.*, 2015; Middleman, 1972
Alveolar cylinder radius		cm	$3.25 \cdot 10^{-3}$	**	
Alveolar O_2 diffusivity		cm^2/s	$1.98 \cdot 10^{-1}$	**	Schmitz *et al.*, 2013
Alveolar oxygen solubility		mL O_2/(mL·mmHg)	1	**	
Mixed venous pO_2 (initial)		mmHg	40	**	Ortiz-Prado *et al.*, 2019; Guyton & Hall, 2006; Mah & Cheng, 2015
Alveolar pO_2		mmHg	104	**	Ortiz-Prado *et al.*, 2019; Guyton & Hall, 2006
Pulmonary vein pO_2		mmHg	99.7	**	Sharma & Hashmi, 2018
Consumption Parameters					
Maximum O_2 consumption rate per unit volume	M_{total}	mL O_2/mL	$1.80 \cdot 10^{-10}$	**	
Michaelis-Menten coefficient	K_m	mmHg	10.5 ± 0.8	0.5	Golub & Pittman, 2012
Max consumption rate per segment	M_0	$\frac{\text{mL } O_2}{\text{mL} \cdot \text{s}}$	$8 \cdot 10^{-10}$	$5 \cdot 8 \cdot 10^{-10}$	Jamal *et al.*, 2015; Sakles *et al.*, 1997
Max consumption rate per segment equation		$M_0 = \frac{M_{\text{total}}}{L_{\text{capillary}} \cdot \pi \cdot \left(r^2_{\text{tissue}} - r^2_{\text{capillary}}\right)}$			Jamal *et al.*, 2015

**: Not implemented in previous model
Arrows (→) indicate referenced citations as sourced in prior literature.
